# Supplementary material for: Early mobilisation and rehabilitation in the PICU: a UK survey
Source: BMJ Paediatr Open. 2022 Jun 9;6(1):e001300. doi: 10.1136/bmjpo-2021-001300 (PMC9185558; doi:10.1136/bmjpo-2021-001300)
Supplement: Supplementary data [file bmjpo-2021-001300supp001.pdf]

Supplementary Material File 1

PERMIT Online Survey

Paediatric Early Rehabilitation and Mobilisation during InTensive care (PERMIT) Survey

1. Introduction

Thank you for your interest in completing a questionnaire as part of the PERMIT Study.

Why have I been invited to take part?
You have been invited to take part because you are a health care practitioner responsible for the delivery of care to children in a PICU. We would like the views of a wide range of health care professionals, and you have been identified either through the collaborating Paediatric Intensive Care Society, Physiotherapy and Occupational therapy membership lists or recommended by lead clinicians in your institutions.

Study Overview
We are conducting research into Early Rehabilitation and Mobilisation (ERM) during paediatric critical illness in the UK and assessing the feasibility and design of a future clinical trial. ERM can encompass patient-tailored interventions or bundles of care provided within the critical care unit. Rehabilitation has been shown to improve quality of life and patient outcomes in the adult ICU; however, there is limited evidence in the PIC patient population and setting.

The whole PERMIT programme aims to assess key questions regarding the acceptability and feasibility of ERM in PIC and form recommendations for a future clinical trial to establish its effectiveness.

In this questionnaire, we would like to find out:
- What forms of ERM are provided in your PICs and to which patients;
- What resources are available (or could be available) to deliver ERM;
- Your views on evaluating ERM interventions;
- Whether you would be interested in a future clinical trial to assess ERM.

Thank you for your time (which we calculated should be less than 20 minutes) in completing this questionnaire.

Barney Scholefield & Jacqueline Thompson

On behalf of the PERMIT study team and the Paediatric Intensive Care Society -Study Group (PICS-SG)

2. A little demographic information about you please

1. Which hospital do you work at? *

Other (if not listed)

2. Which best reflects your current professional position? *

- ☐ Medical
- ☐ Nurse
- ☐ Physiotherapist
- ☐ Occupational therapist
- ☐ Speech and Language therapist
- ☐ Dietician
- ☐ Play therapist
- ☐ Psychologist
- ☐ Other (please specify):

3. Number of years experience working in PICU? *

- ☐ <1 year
- ☐ 1 to <5 years
- ☐ 5 to <10 years
- ☐ 10 to <15 years
- ☐ 15 to <20 years

- ☐ More than 20 years
- ☐ I do not work in PICU

Comments:

3. What ERM do you currently provide in your PICU?

In the following questions, we hope to explore your current practice related to early rehabilitation and mobilisation (ERM). We understand that the term ERM may mean different things to different professional groups; however, we are keen to capture the current diverse views and how ERM is defined by you and your PICU team.

4. Please describe in the box below the current ERM practice within your PICU. (We are especially interested in whether you provide anything that you feel fits within the term ERM, who receives ERM, what specific actions are involved in ERM and who delivers the ERM).

5. Which of the following best describes when you would consider ERM for a patient? *

- ☐ Within 24 hours of admission to PICU
- ☐ About 2 to 3 days after PICU admission
- ☐ Around the end of the first week of PICU admission
- ☐ Before discharge from PICU
- ☐ As soon as the patient is stable enough
- ☐ As soon as the patient is off mechanical ventilatory support
- ☐ To start earlier than we historically considered starting rehabilitation and mobilisation
- ☐ We do not consider ERM for our PICU patients
- ☐ I don't know
- ☐ Other (please specify):

Comments:

6. Which team members are involved in ERM in your PICU? (Please select how frequently they are involved. If the team member is not available in your PICU please select 'Not applicable')

	Always	Very often	Sometimes	Seldom	Never	Not applicable (as team member is not available in my PICU)	Don't know
Medical staff	☐	☐	☐	☐	☐	☐	☐
Nursing staff	☐	☐	☐	☐	☐	☐	☐
Speech and language therapist	☐	☐	☐	☐	☐	☐	☐
Physiotherapist	☐	☐	☐	☐	☐	☐	☐
Occupational therapist	☐	☐	☐	☐	☐	☐	☐
Psychologist	☐	☐	☐	☐	☐	☐	☐
Play therapist	☐	☐	☐	☐	☐	☐	☐
Dietician	☐	☐	☐	☐	☐	☐	☐
Patients family or carers	☐	☐	☐	☐	☐	☐	☐
Other	☐	☐	☐	☐	☐	☐	☐

Please define 'other' or add any comments

7. How often do patients, in general, receive ERM in your PICU?

	Always	Very often	Sometimes	Seldom	Never	Don't know
All PICU patients	☐	☐	☐	☐	☐	☐

Comments:

8. How often do patients in the following age groups receive ERM in your PICU? (Please select an option for each)

	Always	Very often	Sometimes	Seldom	Never	Don't know
Infants & <1 year old	☐	☐	☐	☐	☐	☐
1-4 years old	☐	☐	☐	☐	☐	☐
> 4-10 years old	☐	☐	☐	☐	☐	☐
> 10-18 years old	☐	☐	☐	☐	☐	☐

Comments:

9. How often do patients within the following diagnostic categories receive ERM in your PICU? (Please select an option for each)

	Always	Very often	Sometimes	Seldom	Never	Don't know
Acquired brain injury patients (e.g. TBI, meningitis)	☐	☐	☐	☐	☐	☐
Sepsis	☐	☐	☐	☐	☐	☐
Congenital heart disease	☐	☐	☐	☐	☐	☐
Cancer	☐	☐	☐	☐	☐	☐
Mechanically ventilated	☐	☐	☐	☐	☐	☐

Thompson JY, *et al. BMJ Paediatrics Open* 2022; 6:e001300. doi: 10.1136/bmjpo-2021-001300

	Always	Very often	Sometimes	Seldom	Never	Don't know
Mechanically supported (e.g. ECMO)	☐	☐	☐	☐	☐	☐
Respiratory illness (bronchiolitis or pneumonia)	☐	☐	☐	☐	☐	☐
Multi-organ failure	☐	☐	☐	☐	☐	☐
With pre-existing physical co-morbidity	☐	☐	☐	☐	☐	☐
With pre-existing severe developmental delay	☐	☐	☐	☐	☐	☐

Comments:

10. How often do patients, with the following length of stay, receive ERM in your PICU? (Please select an option for each)

	Always	Very often	Sometimes	Seldom	Never	Don't know
Patients in PICU <3 days	☐	☐	☐	☐	☐	☐
Patients in PICU 3-7 days	☐	☐	☐	☐	☐	☐
Patients in PICU >7-28 days	☐	☐	☐	☐	☐	☐
Patients in PICU >28 days	☐	☐	☐	☐	☐	☐

Comments:

11. Who decides if the patient can start to receive ERM? (Please select all that apply)

☐ Medical consultant

☐ Other member of the medical team (e.g. PICU trainee/ advanced nurse practitioner)

☐ Bedside nurse

☐ Senior nurse on shift

☐ Physiotherapist

☐ Occupational therapist

☐ Parent(s)

☐ Don't know

☐ Not applicable

☐ Other (please specify):

Comments:

12. How does your PICU assess whether patients tolerate and cope with the therapy provided? (eg monitor safety)

13. Do you currently use or have an established guideline or protocol regarding management of ERM in your PICU?

☐ No

☐ Yes - if you are able to share, please email to b.scholefield@bham.ac.uk

☐ Don't know

Comments:

4. Question regarding your established ERM programme or protocol

14. What do you have within your protocolised ERM treatment? (Please select all that apply)

- ☐ Physical therapy not requiring additional equipment
- ☐ Physical therapy requiring additional equipment
- ☐ Occupational therapy
- ☐ Speech and language therapy
- ☐ Psychology
- ☐ Delirium screening
- ☐ We do not have a programme or protocol related to ERM activities
- ☐ Other (please specify):

Comments:

15. What are the key factors in successfully implementing your ERM protocol?

5. ERM related processes and equipment in your PICU

The following three questions are related to additional processes or equipment in your PICU related to ERM activities.

16. Does your PICU have a protocol or guidelines for the management of the following?

	Yes	No	Don't know
Sedation	☐	☐	☐
Analgesia	☐	☐	☐
Drug/ opioid withdrawal	☐	☐	☐
Delirium	☐	☐	☐

	Yes	No	Don't know
Mobilisation (at any time point)	☐	☐	☐

17. What types of therapies or activities are available in your PICU? (Please select all that apply)

- ☐ Physical therapy - not requiring additional equipment (e.g. passive or active movements)
- ☐ Physical therapy - requiring additional equipment (e.g. in bed cycling)
- ☐ Occupational therapy
- ☐ Speech and language therapy
- ☐ Clinical psychology
- ☐ Delirium screening
- ☐ Patient diaries
- ☐ Play therapy
- ☐ Pet therapy
- ☐ Music therapy
- ☐ Other (please specify):

18. What equipment does your PICU have available for physical therapy/mobilisation? (Please select all that apply)

- ☐ Tilt table
- ☐ Bed with Trendelenburg features
- ☐ Bed with full chair position
- ☐ Bed with chair egress - exit out the foot of the bed
- ☐ Speciality bed with continuous side to side rotation
- ☐ Bed with retractable footboard i.e. flex a foot feature

- [ ] Mobile lifts
- [ ] Ceiling lifts
- [ ] Portable ventilators
- [ ] Patient rolling walker
- [ ] Bedside cycle (patient can remain in bed)
- [ ] Transcutaneous electrical stimulation of muscles
- [ ] Specialist wheelchair
- [ ] Specialist static seating i.e. tumble forms
- [ ] Other (please specify):

6. Preferences, barriers and outcomes

For the following questions, we would explore your strength of opinion on ERM as a therapy, potential barriers to using ERM in practice and which are the most important clinical outcomes to consider when assessing if ERM is beneficial.

19. Please select one option regarding your current views of ERM in your PICU? *

- [ ] Crucial, should be the top priority in the care of PICU patients
- [ ] Very important, should be a priority in the care of PICU patients
- [ ] Important, should be a priority in the care of PICU patients
- [ ] Somewhat important, should be considered in the care of PICU patients
- [ ] Not of great importance, clinicians should bear it in mind in the care of PICU patients
- [ ] Of minimal importance to the care of PICU patients
- [ ] Of no importance to the care of PICU patients

Comments:

20. Below are potential institutional barriers to the delivery of ERM. Please select to what extent you agree/disagree that each are barriers to delivery of ERM in your PICU. *

	Strongly Agree	Agree	Neutral	Disagree	Strongly Disagree
Instructions that patients should not move in their bed	☐	☐	☐	☐	☐
Consultant's permission or prescription is required prior to mobilisation	☐	☐	☐	☐	☐
Insufficient equipment/ resources	☐	☐	☐	☐	☐
Inadequate funding	☐	☐	☐	☐	☐
Lack of written guidelines/ protocol	☐	☐	☐	☐	☐
Lack of physical space	☐	☐	☐	☐	☐
Absence of champion/ advocate to promote ERM	☐	☐	☐	☐	☐
Absence of frequent (e.g. daily) ERM patient screening	☐	☐	☐	☐	☐

Comments:

21. Below are potential patient barriers to the delivery of ERM. Please select to what extent you agree/disagree that each are barriers to delivery of ERM in your PICU. *

	Strongly Agree	Agree	Neutral	Disagree	Strongly Disagree
Physiological instability ('the patient is just too sick')	☐	☐	☐	☐	☐

	Strongly Agree	Agree	Neutral	Disagree	Strongly Disagree
Sedation level	☐	☐	☐	☐	☐
Presence of endotracheal intubation	☐	☐	☐	☐	☐
Risk of device/ line/ catheter dislodgement	☐	☐	☐	☐	☐
Cognitive impairment/ age	☐	☐	☐	☐	☐
Obesity	☐	☐	☐	☐	☐
Patient motivation	☐	☐	☐	☐	☐
Family concerns	☐	☐	☐	☐	☐

Comments:

22. Below are potential provider barriers to the delivery of ERM. Please select to what extent you agree/disagree that each are barriers to delivery of ERM in your PICU. *

	Strongly Agree	Agree	Neutral	Disagree	Strongly Disagree
Lack of prioritisation of ERM within patient care plans	☐	☐	☐	☐	☐
Limited staffing to deliver ERM	☐	☐	☐	☐	☐
Lack of coordination within and between clinician groups	☐	☐	☐	☐	☐
Slow recognition of patient readiness for ERM	☐	☐	☐	☐	☐
Conflicting perceptions concerning patient suitability	☐	☐	☐	☐	☐

	Strongly Agree	Agree	Neutral	Disagree	Strongly Disagree
Safety concerns	☐	☐	☐	☐	☐
Lack of specific decision-making authority for ERM initiation	☐	☐	☐	☐	☐
Inadequate training	☐	☐	☐	☐	☐
Prolonging the current working day	☐	☐	☐	☐	☐

Comments:

23. Please rank what you perceive to be the top 5 most important benefits that ERM could provide in PICU. (Please select, in order, the top perceived benefits by selecting a box). *

	1 (most important)	2	3	4	5 (least important)
Reduction in treatment cost	☐	☐	☐	☐	☐
Reduction in patient delirium	☐	☐	☐	☐	☐
Reduction in length of ICU stay	☐	☐	☐	☐	☐
Reduction in days requiring mechanical ventilation	☐	☐	☐	☐	☐
Reduction in the rate of pulmonary complications	☐	☐	☐	☐	☐
Reduction in readmission (hospital and PICU)	☐	☐	☐	☐	☐
Increase in number of patients discharged home	☐	☐	☐	☐	☐
Improved patient satisfaction	☐	☐	☐	☐	☐

Thompson JY, *et al. BMJ Paediatrics Open* 2022; 6:e001300. doi: 10.1136/bmjpo-2021-001300

	1 (most important)	2	3	4	5 (least important)
Improved staff satisfaction	☐	☐	☐	☐	☐
Improved family satisfaction	☐	☐	☐	☐	☐
Improvement in patient sleep quality	☐	☐	☐	☐	☐
Improvement in psychological impact of PICU care	☐	☐	☐	☐	☐
Improvement in daily life participation following discharge	☐	☐	☐	☐	☐

Comments:

7. Final thoughts

24. Would you be willing to support a future clinical trial in the UK and Ireland based on evaluating ERM practice?

☐ Yes

☐ No

☐ Don't Know

Comments:

25. Would you allow us to contact you in the future with information regarding workshops we will be running to discuss ERM practice and trial feasibility, please? *

☐ Yes (please click the link on the next page to provide best contact information, thank you!)

☐ No

Thompson JY, *et al. BMJ Paediatrics Open* 2022; 6:e001300. doi: 10.1136/bmjpo-2021-001300

Funding statement: This study/project is funded by the National Institute for Health Research (NIHR) [Health Technology Assessment Programme (project reference: 17/21)/ HTA]. The views expressed are those of the author(s) and not necessarily those of the NIHR or the Department of Health and Social Care.
