# Supplementary material for: Early mobilisation and rehabilitation in the PICU: a UK survey
Source: BMJ Paediatr Open. 2022 Jun 9;6(1):e001300. doi: 10.1136/bmjpo-2021-001300 (PMC9185558; doi:10.1136/bmjpo-2021-001300)
Supplement: Supplementary data [file bmjpo-2021-001300supp002.pdf]

Supplementary Material File 2

Table 1: Descriptions of ERM

Category	Sub-categories	Examples:
**Activity-focused**<br>ERM is activity-focused, with consideration of seating and equipment, primarily delivered by nursing and allied health care professionals with optimised sedation management to promote patient engagement.<br>There is a lack of consensus on what is routine care compared with purposeful ERM activity.	Activity – mobilising, positioning, stretching	"ERM provided includes - passive movements of limbs, periodic change of position, use of splints on extremities and if stable in the long term patients - sitting up in tumble form chair and mobilising out of bed." (Doctor, 67)<br><br>"We aim to get the children sitting upright, either over the edge of the bed or inappropriate seating as soon as possible." (Occupational Therapist, 001)<br><br>"Nursing staff are taught the importance of regular position changes for patient ... and positioning to maintain ranges of movement and prevent foot drop." (Nurse, 017)
	Core health care professional involvement – physiotherapist, nurse, occupational therapist	"We try and mobilise patients as soon as able when not invasively ventilated - physio led." (Doctor, 058)<br><br>"As physiotherapists, we work with the occupational therapist and nursing staff to either re-position, sit up in bed, sit on the edge of the bed or stand whilst on the ventilators."(Physiotherapist, 069)
	Additional health care professional involvement – psychology, dietician, play therapist, speech and language therapists	"Speech and language therapy become involved usually when nursing or medical staff identify a need for referral." (Doctor, 003)<br><br>"We have a Play Specialist on PICU, who assists with communication tools/toys." (Nurse, 082)
	Seating and equipment	"Specialist seating need – linking in with OT to ensure appropriate seating available." (Physiotherapist, 019)

Thompson JY, *et al. BMJ Paediatrics Open* 2022; 6:e001300. doi: 10.1136/bmjpo-2021-001300

		"We have also in the past asked adult [services] to use their moto-med bike and used this with teenagers, but this can be a challenge as it is very far from PICU and it is often in use." (Physiotherapist, 014)
	Sedation management	"Working with the medics to wean sedation as quickly as possible. This promotes faster ability to mobilise and progress in their rehabilitation." (Nurse, 034)
	'Routine care' vs 'purposeful ERM package'	Some centres define ERM as passive range of movement, repositioning or providing splints. We would deem this as essential core cares rather than ERM; the large majority of our patients will have positioning and movement plan from day 1 of admission." (Physiotherapist, 111)
**Tailored**<br>Following the assessment of needs and patient and family preference, ERM activities are personalised to the individual patient.	Assessment of need	"We have a programme where patients are categorised to one of 3 levels. Each level provides nursing and therapy staff with activities aligned to the acuity of the patient." (Nurse, 040)
	Individual preference	"Parents will tell us what their child enjoys doing and make suggestions, often provide toys/games from home." (Nurse, 076)<br><br>"Discuss with parents what patient enjoys doing, watching etc. Discuss options regarding taking patients 'out' where possible if long term patients." (Nurse, 015)
**Promote recovery**<br>The purpose of ERM is to normalise the PICU environment, to create an environment that addresses holistic needs, sustains or promotes development<br>and supports recovery from critical illness.	Normalising PICU environment	"Provide activities they would use for their enjoyment. This enables them to be more relaxed and less aware of what is going on with / around them." (Play specialist, 102)<br><br>"Being in PICU can be a frightening experience. Not only is a child/young person away from home, but also away from their usual environment, family and friends. There are unfamiliar sounds, smells, equipment and people. The play specialist can help to make the stay much more enjoyable and children/young people to cope and understand." (Play specialist, 055)
	Sustain / promote child development	"Encouraging the patient to regain or further their development in a therapeutic fun manner." (Play therapist,102)

	Restoration / recovery	"Interventions aim to promote physical recovery – movement, and ability to engage in activities and psychological recovery – orientation, speech, ability to play and attend school. " (Nurse, 021)
**Timing of ERM**<br>The optimal timing of ERM initiation is challenging for health care professionals to define, although likelihood perceived to increase with increased length of stay. Influenced by the perceived stability of patients and the balance of risk to benefit.	'Early' poorly defined	"I like to think we consider it within 2-3 days we have a better idea of the patients PICU journey and how long they are going to stay. The reality is it's usually longer... usually week 2 of admission if they are still on the unit." (Physiotherapist, 090)<br><br>"There is no formal plan for who decides if a patient should start ERM and when… Currently the delivery of ERM is fairly ad hoc, based on what is highlighted from daily handover each morning." (Physiotherapist, 083)<br><br>"We provide ERM as a structured programme… every patient after 24 hours of admission is considered for ERM." (Doctor, 012)
	Patient stability, risk and benefit	"Members of the PICU team with less experience of early rehab can deem ERM 'unsafe' which can occasionally act as a barrier."(Physiotherapist, 016)<br><br>"Usually ERM activity is not considered until patients can physiologically tolerate movement and are stable with observations." (Nurse, 008)
	'Long-stay'	"ERM is often thought about as a patient comes up to extubation or has failed extubation, and we feel that the reason for failure may be because of critical care weakness." (Physiotherapist, 031)<br><br>"We provide very little ERM on PICU, other than for long term patients, which is often, in my opinion, delayed." (Nurse, 007)

Table 2: Reported frequency of receiving or being involved in Early Rehabilitation and Mobilisation by age groups, length of PICU stay, diagnostic category, and health care professional or parental/family role (n=124 respondents)*

By age group	Always (n, %)	Very often (n, %)	Sometimes (n, %)	Seldom (n, %)	Never (n, %)	Don't know (n, %)	Not Applicable# n
All PICU Patients (any age)	6 (5)	45 (36)	48 (39)	14 (11)	0 (0)	11 (9)	0
>10-18 years old	18 (15)	40 (32)	43 (35)	8 (6)	1 (1)	14 (11)	0
>4-10 years old	16 (13)	42 (34)	41 (33)	10 (8)	1 (1)	14 (11)	0
1-4 years old	13 (10)	42 (34)	39 (31)	13 (10)	1 (1)	16 (13)	0
Infants & <1 year old	6 (5)	47 (38)	32 (26)	20 (16)	3 (2)	16 (13)	0
**By length of stay in PICU**							
PICU >28 days	43 (35)	48 (39)	14 (11)	4 (3)	0 (0)	15 (12)	0
PICU >7-28 days	27 (22)	46 (37)	26 (21)	8 (6)	0 (0)	17 (14)	0
PICU 3-7 days	12 (10)	32 (26)	40 (32)	19 (15)	5 (4)	16 (13)	0
PICU <3 days	3 (2)	14 (11)	39 (31)	33 (27)	17 (14)	18 (15)	0
**By diagnosistic category**							
Acquired brain injury	36 (29)	39 (31)	16 (13)	11 (9)	4 (3)	18 (15)	0
Severe developmental delay	11 (9)	43 (35)	39 (31)	12 (10)	3 (2)	16 (13)	0
Cancer	11 (9)	24 (19)	36 (29)	14 (11)	4 (3)	35 (28)	0
Pre-existing physical morbidity	9 (7)	38 (31)	44 (35)	12 (10)	4 (3)	17 (14)	0
Mechanically ventilated	9 (7)	38 (31)	39 (31)	18 (15)	5 (4)	15 (12)	0
Congenital heart disease	9 (7)	24 (19)	39 (31)	15 (12)	6 (5)	31 (25)	0
Respiratory illness	7 (6)	35 (28)	39 (31)	22 (18)	3 (2)	18 (15)	0
Sepsis	7 (6)	28 (23)	44 (35)	20 (16)	5 (4)	20 (16)	0
Multi-organ failure	5 (4)	14 (11)	32 (26)	35 (28)	12 (10)	26 (21)	0
Mechanically supported (e.g. extracorporeal life support)	4 (3)	8 (6)	14 (11)	13 (10)	47 (38)	38 (31)	0
**Health Care Professional team members & parent or family member(s) involvement in ERM (when applicable#)**							
Physiotherapist	86 (70)	27 (22)	4 (3)	2 (2)	0 (0)	4 (3)	1

4

Nurse	54 (44)	49 (40)	9 (7)	5 (4)	0 (0)	6 (5)	1
Parent or family member	36 (29)	56 (46)	19 (15)	7 (6)	1 (1)	4 (3)	1
Occupational therapist	23 (19)	34 (29)	29 (24)	20 (17)	6 (5)	7 (6)	5
Doctor	22 (18)	20 (16)	26 (21)	30 (24)	13 (11)	12 (10)	1
Play therapist	12 (10)	24 (21)	39 (34)	26 (23)	9 (8)	5 (4)	9
Dietician	10 (8)	20 (17)	12 (10)	29 (24)	37 (31)	13 (11)	3
Speech and Language therapist	4 (3)	11 (9)	40 (33)	35 (29)	20 (17)	10 (8)	4
Psychologist	3 (3)	11 (10)	24 (23)	32 (30)	30 (28)	6 (6)	18

***** Percentages may not total 100 due to rounding

[#] Not applicable (e.g. not available in PICU) responses excluded from percent calculation.

Thompson JY, *et al. BMJ Paediatrics Open* 2022; 6:e001300. doi: 10.1136/bmjpo-2021-001300

Table 3: Barriers of ERM delivery within Paediatric Intensive Care Units (n=124 respondents)*

**Institutional barriers**	**Strongly Agree n (%)**	**Agree n (%)**	**Neutral n (%)**	**Disagree n (%)**	**Strongly Disagree n (%)**	**Skipped responses[#]** (n)
Insufficient equipment/resources	31 (26)	52 (43)	22 (18)	13 (11)	3 (2)	3
Lack of written guidelines/protocol	31 (26)	38 (31)	35 (29)	11 (9)	6 (5)	3
Inadequate funding	30 (25)	43 (36)	28 (23)	16 (13)	4 (3)	3
Absence of champion/advocate to promote ERM	25 (21)	43 (36)	23 (19)	21 (17)	9 (7)	3
Absence of frequent ERM patient screening	25 (23)	38 (35)	24 (22)	14 (13)	8 (7)	14
Lack of physical space	17 (14)	51 (42)	17 (14)	32 (26)	4 (3)	3
Instructions that patients should not move in their bed	10 (8)	25 (21)	24 (20)	48 (40)	14 (12)	3
Consultant's permission or prescription is required prior to mobilisation	5 (4)	39 (32)	32 (26)	33 (27)	12 (10)	3
**Provider barriers**						
Slow recognition of patient readiness for ERM	19 (16)	46 (38)	19 (16)	35 (29)	2 (2)	3
Limited staffing to deliver ERM	42 (35)	54 (45)	11 (9)	13 (11)	1 (1)	3
Lack of prioritisation of ERM within patient care plans	27 (22)	45 (37)	22 (18)	24 (20)	3 (2)	3
Inadequate training	20 (17)	54 (45)	18 (15)	26 (21)	3 (2)	3
Conflicting perceptions concerning patient suitability	17 (14)	59 (49)	22 (18)	21 (17)	2 (2)	3
Lack of specific decision-making authority for ERM initiation	16 (13)	49 (40)	28 (23)	24 (20)	4 (3)	3
Safety concerns	15 (12)	55 (45)	32 (26)	18 (15)	1 (1)	3
Lack of coordination within and between clinician groups	14 (12)	44 (37)	30 (25)	27 (23)	5 (4)	4
Prolonging the current working day	4 (3)	21 (17)	43 (36)	41 (34)	12 (10)	3
**Patients barriers**						
Levels of physiological instability	48 (40)	53 (44)	12 (10)	7 (6)	1 (1)	3
Sedation level	27 (22)	64 (53)	15 (12)	13 (11)	2 (2)	3
Presence of endotracheal intubation levels	8 (7)	36 (30)	32 (26)	39 (32)	6 (5)	3
Risk of device/line/catheter dislodgement levels	8 (7)	42 (35)	32 (26)	35 (29)	4 (3)	3
Cognitive impairment/age levels	1 (1)	32 (26)	29 (24)	46 (38)	13 (11)	3
Obesity levels	6 (5)	14 (12)	35 (29)	52 (43)	14 (12)	3

Thompson JY, *et al. BMJ Paediatrics Open* 2022; 6:e001300. doi: 10.1136/bmjpo-2021-001300

Patient motivation level	2 (2)	38 (31)	31 (26)	43 (36)	7 (6)	3
Family concerns levels	2 (2)	42 (35)	35 (29)	38 (31)	4 (3)	3

* Percentages may not total 100 due to rounding # Skipped responses excluded from percent calculation.

Thompson JY, *et al. BMJ Paediatrics Open* 2022; 6:e001300. doi: 10.1136/bmjpo-2021-001300
