# Supplementary material for: Early mobilisation and rehabilitation in the PICU: a UK survey
Source: BMJ Paediatr Open. 2022 Jun 9;6(1):e001300. doi: 10.1136/bmjpo-2021-001300 (PMC9185558; doi:10.1136/bmjpo-2021-001300)
Supplement: Author's manuscript [file bmjpo-2021-001300.draft_revisions.pdf]

BMJ Paediatrics Open

BMJ Paediatrics Open is committed to open peer review. As part of this commitment we make the peer review history of every article we publish publicly available.

When an article is published we post the peer reviewers' comments and the authors' responses online. We also post the versions of the paper that were used during peer review. These are the versions that the peer review comments apply to.

The versions of the paper that follow are the versions that were submitted during the peer review process. They are not the versions of record or the final published versions. They should not be cited or distributed as the published version of this manuscript.

BMJ Paediatrics Open is an open access journal and the full, final, typeset and author-corrected version of record of the manuscript is available on our site with no access controls, subscription charges or pay-per-view fees (http://bmjpaedsopen.bmj.com).

If you have any questions on BMJ Paediatrics Open's open peer review process please email info.bmjpo@bmj.com

BMJ Paediatrics Open

Early Mobilisation and Rehabilitation in the PICU: A UK survey

Journal:	*BMJ Paediatrics Open*
Manuscript ID	bmjpo-2021-001300
Article Type:	Original research
Date Submitted by the Author:	21-Sep-2021
Complete List of Authors:	Thompson, Jacqueline; University of Birmingham, ; University of Birmingham<br>Menzies, Julie; Birmingham Women's and Children's NHS Foundation Trust, Paediatric Intensive Care<br>Manning, Joseph ; Nottingham University Hospitals NHS Trust, Nottingham Children's Hospital; University of Nottingham, Children and Young people Health Research, School of Health Sciences<br>McAnuff, Jennifer; Newcastle University, Population Health Sciences Institute, Newcastle University; Northumbria University, Department of Social Work, Education and Community Wellbeing<br>Brush, E; University of Birmingham<br>Ryde, F; University of Birmingham<br>Rapley, Tim; Northumbria University<br>Pathan, Nazima; University of Cambridge, Paediatrics<br>Brett, S; Imperial College of Science Technology and Medicine, Department of surgery and cancer<br>Moore, D; University of Birmingham<br>Geary, M; University Hospital Southampton NHS Foundation Trust, Child Health<br>Colville, Gillian; St George's University Hospitals NHS Foundation Trust, Paediatric Psychology Service<br>Morris, KP; Birmingham Women's & Children's NHS Foundation Trust<br>PARSLOW, ROGER; UNIVERSITY OF LEEDS, PAEDIATRIC EPIDEMIOLOGY GROUP<br>Feltbower, Richard; UNIVERSITY OF LEEDS<br>Lockley, S<br>Kirkham, Fenella J.; Neurosciences Unit, UCL Institute of Child Health<br>Forsyth, Rob; Newcastle University, Institute of Neuroscience<br>Scholefield, Barney; University of Birmingham
Keywords:	Rehabilitation, Epidemiology

SCHOLARONE™
Manuscripts

I, the Submitting Author has the right to grant and does grant on behalf of all authors of the Work (as defined in the below author licence), an exclusive licence and/or a non-exclusive licence for contributions from authors who are: i) UK Crown employees; ii) where BMJ has agreed a CC-BY licence shall apply, and/or iii) in accordance with the terms applicable for US Federal Government officers or employees acting as part of their official duties; on a worldwide, perpetual, irrevocable, royalty-free basis to BMJ Publishing Group Ltd ("BMJ") its licensees and where the relevant Journal is co-owned by BMJ to the co-owners of the Journal, to publish the Work in this journal and any other BMJ products and to exploit all rights, as set out in our licence.

The Submitting Author accepts and understands that any supply made under these terms is made by BMJ to the Submitting Author unless you are acting as an employee on behalf of your employer or a postgraduate student of an affiliated institution which is paying any applicable article publishing charge ("APC") for Open Access articles. Where the Submitting Author wishes to make the Work available on an Open Access basis (and intends to pay the relevant APC), the terms of reuse of such Open Access shall be governed by a Creative Commons licence – details of these licences and which Creative Commons licence will apply to this Work are set out in our licence referred to above.

Other than as permitted in any relevant BMJ Author's Self Archiving Policies, I confirm this Work has not been accepted for publication elsewhere, is not being considered for publication elsewhere and does not duplicate material already published. I confirm all authors consent to publication of this Work and authorise the granting of this licence.

Confidential: For Review Only

Early Mobilisation and Rehabilitation in the PICU: A UK survey

Manuscript type: Original Research

Authors:

Thompson JY[1], Menzies JC[2], Manning JC[3,4], McAnuff J[5, 6], Brush E[1], Ryde F[1], Rapley TR[6], Pathan N[7], Brett S[8], Moore D[9], Geary M[10], Colville G[11], Morris KP[2], Parslow RC[12], Feltbower RG[12], Lockley S[13], Kirkham F[10], Forsyth R[14], Scholefield B R[1,2], On behalf of the PERMIT Collaborators & Paediatric Intensive Care Society Study-Group (PICS-SG)

Author affiliations:

1. Birmingham Acute Care Research Group, Institute of Inflammation and Ageing, University of Birmingham
2. Paediatric Intensive Care, Birmingham Women's & Children's NHS Foundation Trust
3. Nottingham Children's Hospital, Nottingham University Hospitals NHS Trust
4. Children and Young People Health Research, School of Health Sciences, The University of Nottingham
5. Population Health Sciences Institute, Newcastle University
6. Department of Social Work, Education and Community Wellbeing, Northumbria University
7. Department of Paediatrics, The Chancellor, Masters, and Scholars of the University of Cambridge
8. Department of surgery and cancer; Imperial College of Science, Technology and Medicine

Confidential: For Review Only

9. Institute of Applied Health, University of Birmingham

10. Child Health, University Hospital Southampton NHS Foundation Trust

11. Paediatric Psychology Service; St George's University Hospitals, NHS Foundation Trust

12. Leeds Institute for Data Analytics, School of Medicine, University of Leeds, Leeds

13. PPI Representative

14. Translational and Clinical Research Institute, Newcastle University

Corresponding author:

Barnaby R Scholefield; B.Scholefield@bham.ac.uk

Twitter handle: @BarneyUoB

Abstract word count: 248 words now

Main text word count (excluding abstract, reference list, tables and figures): 2507

References: 25

Tables: 3

Figures: 2

Supplementary files: 2 (1 survey, 6 supplemental tables)

Confidential: For Review Only

Abstract

Objective: To understand the context and professional perspectives of delivering early rehabilitation and mobilisation (ERM) within UK Paediatric Intensive Care Units (PICU).

Design: A web-based survey administered May-August 2019.

Setting: United Kingdom PICUs.

Participants: A total of 124 staff from 26 PICUs participated, including 22 (18%) Doctors, 34 (27%) Nurses, 28 (23%) Physiotherapists and 40 (32%) other professionals.

Results: A strong multi-disciplinary involvement in initiating ERM was reported. All age groups were considered for ERM. Over half of respondents favoured delivering ERM after physiological stability had been achieved (n=69, 56%). ERM was defined by participants as consisting of tailored, multi-disciplinary rehabilitation packages, focused on promoting recovery. ERM was reported to be more likely to be delivered to patients when PICU length of stay exceeded 28 days and among patients with acquired brain injury or severe developmental delay. The most commonly identified barriers were: insufficient resources and equipment (69%), inadequate funding (60%), limited staffing (79%), lack of recognition of patient readiness (67%), patient suitability (63%), inadequate training (61%), physiological instability (81%) and sedation requirement(73%). Respondents ranked 'reduction in PICU length of stay' 74% and 'improvement in psychological outcomes' 73% as the most important benefits of ERM.

Confidential: For Review Only

Conclusion: ERM is gaining familiarity and endorsement in UK PICUs, but significant barriers to implementation due to limited resources and variation in content and delivery of ERM persist. A standardised protocol that sets out defined ERM interventions, along with implementation support to tackle modifiable barriers is required to ensure delivery of high-quality ERM.

Confidential: For Review Only

INTRODUCTION

In the UK, approximately 20,000 children are admitted to Paediatric Intensive Care Units (PICU) yearly[1], and although most recover, some develop longer-term physical, psychological and cognitive impairment[2]. These significant morbidities have been termed Post Intensive Care Syndrome in Paediatrics (PICS-p)[3]. Early rehabilitation and mobilisation (ERM) encompasses patient-tailored interventions, delivered individually[4] or in a bundled package[5] to patients within intensive care settings. It is provided by health professionals from multiple disciplines and care-givers and may promote physical (e.g. functional activities)[4] and non-physical (e.g. psychological, cognitive)[6] recovery.

Within adult ICUs, ERM has been demonstrated to be safe, feasible and cost-effective[7 8]. It can shorten the length of ventilation, duration of intensive care and hospital stay with economic benefit, improve long term physical functioning and return to independence, and is recommended by National Institute for Health Care Excellence (NICE)[8]. Whilst ERM has been reported to be safe and feasible in PICUs [9 10], the patient population is different, and ERM is less well defined, leading to variability in practice. At present, no national standardised care pathway for ERM exists within UK PICUs.

This study forms part of the National Institute for Health Research (NIHR) funded PERMIT study (NIHR HTA: 17/21/06) investigating ERM in children. This work set out to understand reported practices and perceptions of ERM within the UK PICU context.

Objectives

We aimed to:

Confidential: For Review Only

I. Explore how health care professionals describe ERM

II. Identify current ERM practice

III. Understand perceived barriers and facilitators of ERM

METHODS

A web-based survey (administered through www.smartsurvey.co.uk) was developed that included 25 questions (Supplementary material file 1). A patient representative was involved in the design and development of the survey. A pilot survey was conducted among multi-disciplinary teams of health professionals (n=40) within two PICUs to assess acceptability and comprehensiveness. Only minor changes to improve question clarity were made. Pilot responses were excluded from the main survey analysis.

A UK Paediatric Intensive Care Society Study Group (PICS-SG) member of each UK PICU (n=29) was contacted via email and requested to identify and cascade to members of their local multi-disciplinary team (including at least one physiotherapist, doctor and nurse) to complete the survey. Participating PICUs were sent a survey link between May and August 2019 to distribute. Three follow up reminders were sent at weekly intervals to non-responders.

Categorical responses were expressed as numbers (percentage), Likert scales (median IQR) were used to express the frequency of practice or level of agreement. Ranking of perceived ERM benefits was calculated using the sum of ranked scores of respondents top 5 important benefits (5 points for 1st, reducing to 1 point for 5th placed). Statistical analysis was

Confidential: For Review Only

performed using R version x64 3.5.1 (R Foundation for Statistical Computing, Vienna, Austria).

Open-ended responses were analysed using a qualitative content analysis approach[11]. Two researchers independently familiarised themselves with the data and conducted open-coding, utilising NVIVO™ software for data management. Codes were then discussed, summarised and organised[12 13]. In relevant sections of the paper, free text quotes from respondents are reported to add context and clarity[14]. The University of Birmingham granted institutional ethical approval on 05/02/2019 (reference ERN_18-1134). Consent was implied through survey completion.

Patient and Public Involvement

We recruited a patient representative as part of the study team to ensure the perspective, experiences and preferences of children admitted to PICU were incorporated into the design and development of the survey. The patient representative was a parent of a child who had been admitted to PICU. They both contributed to the nature of questions considered in the survey to ensure the study outcome would be relevant to parents and members of the UK Paediatric Intensive Care Society Study Group (PICS-SG) on dissemination.

RESULTS

Demographics

Confidential: For Review Only

We received responses from PICS-SG link members in 26/29 (90%) UK PICUs. A total of 191 healthcare professionals opened the survey link, with 124 (65%) submitting responses, with a median of 4.5 participants (IQR 3 to 6) per PICU.

As shown in Supplemental Table 1, the majority of respondents were nurses (n=34, 27%), physiotherapists (n=28, 23%) and doctors (n=22, 18%). There were also responses from occupational therapists (n=19, 15%), play therapists (n=7, 6%), psychologists (n=7, 6%), dieticians (n=6, 5%), and speech and language therapists (n=1, 1%). Almost three-quarters of health professionals had ≥5 years experience, with 48 (39%) ≥15 years. The majority of respondents considered ERM to be a priority, either crucial 15 (12%), very important 67 (55%) or important 35 (29%) in the care of PICU patients (see Supplemental Table 2).

Description of ERM

We invited respondents to describe ERM on their own terms. Descriptions were provided by 104 (84%), which were summarised into four categories, 'Activity-focused', 'Tailored', 'Promote recovery', and 'Timing of ERM' (see Table 1). Overall, ERM was considered to be an individualised package of graded interventions, based on an activity-focused programme, to reduce the sequelae of critical illness or injury. However, responses differed for when ERM should be initiated, often emphasising the need for individualisation.

Availability of established ERM Protocols

Respondents were asked to describe the content of established ERM protocols within their PICU. Only 12 participants (10%) reported working in a PICU with an established ERM protocol (5/26 (19%) of PICUs). The most common components of ERM protocols were

Confidential: For Review Only

'physical therapy not requiring additional equipment' (9/12, 75%) and 'occupational therapy interventions' (8/12, 67%). Only 4/12 (33%) referred to play therapy or speech and language therapy, and no ERM protocol specified input from psychologists or psychiatrists. All participants were asked about the content of non-ERM protocols in their PICU. Only 18/124 (15%) reported that guidance for physical or occupational therapy activities existed in other, non-ERM protocols within PICU (Table 2).

Recipients of ERM

51 (41%) of respondents reported that *All PICU patients* 'always' or 'very often' received ERM (Table 3). Overall, 14 (11%) respondents reported 'seldom' or 'never' delivering ERM.

ERM was reported to be more likely to be delivered to patients when PICU length of stay exceeded 28 days. Patients admitted for 28 days or more were more likely (91, 75%) to 'always' or 'very often' receive ERM in comparison to only 17 (13%) of those reported to stay fewer than three days. Participants reported that patients with acquired brain injury (75, 60%) and severe developmental delay (54, 44%) were 'always' or 'very often' likely to receive ERM.

Perceived benefits of ERM

Participants ranked the five most important potential benefits of ERM out of thirteen options (Figure 1 and Supplemental Table 3). The most important outcomes identified were: 1) reduced PICU length of stay, 2) improved psychological outcomes for patients after PICU, 3) reduced days of mechanical ventilation, 4) improved participation in activities of daily living and 5) improved patient satisfaction.

Confidential: For Review Only

Initiation and delivery of ERM

The decision for ERM initiation was perceived by 96 (77%) to be primarily led by physiotherapists, doctors (92, 74%), and bedside nurses (64, 52%). Parents were felt to initiate ERM by only (24, 19%) of respondents (Supplemental Table 4).

The most influential factor in ERM initiation was reported to be patient stability (69, 56%). Other influential factors were length of stay; 15 (12%) reported ERM was initiated within 24 hours, and 16 (13%) within 2-3 days of PICU admission. Only 5 (4%) of respondents would not consider ERM at all on PICU. The influence of perceived clinical stability is demonstrated in respondents' free-text comments:

> "We are involved as early as required depending on the child/young person medical stability and their rehabilitation needs." (Occupational Therapist, 033)

> "Usually ERM activity is not considered until patients can physiologically tolerate movement and are cardiovascularly stable." (Nurse, 008)

Assessment of patient stability and tolerance of ERM was less well described. Most respondents (98, 79%) provided subjective cues or informal clinical criteria. These included monitoring vital signs, physiological changes, observation of behavioural changes and documentation of adverse events.

Physiotherapists (113, 92%), nurses (103, 84%), and parents or family members (92, 75%) were 'always' or 'very often' involved in the ongoing delivery of ERM with less frequent input from other members of the multi-disciplinary teams (Table 3).

Confidential: For Review Only

Barriers to ERM Implementation

Figure 2 (and Supplemental Table 5) presents the perceived barriers of ERM. The most significant factors identified as barriers at the institutional levels were insufficient resources and equipment (‘strongly agree’ or ‘agree’: 83, 69%) and inadequate funding (73, 61%). Participants provided examples of resources having to be shared across organisations or having to be specially ordered to deliver ERM to patients.

> “All equipment shared with the whole therapy department at present, therefore dependent on availability.” (OT, 010)

A lack of established protocols (69, 57%), ERM champions (68, 57%), space (68, 56%), and robust patient screening processes (63, 58%) were also issues identified by respondents. Most PICUs had access to standard lifting 22/26 (85%) and specialist static seating equipment 25/26 (96%); however, bedside or in-bed cycling machines were only available in 10 (38%) of PICUs (see Supplemental Table 6).

Limited staffing was the most frequently reported barrier to ERM being delivered by providers, with 101 (79%) ‘agreeing’ or ‘strongly agreeing’. Approximately half of the respondents agreed that issues such as training, patient safety, lack of decision-making authority, and delays in recognising patients' ERM needs were barriers to ERM initiation. However, only 25 (21%) ‘agreed’ or ‘strongly agreed’ that the impact of ERM potentially prolonging the working day was a barrier.

Confidential: For Review Only

The two most frequently reported barriers to deliver ERM at the patient level were physiological instability (101, 81% agreeing or strongly agreeing) and sedation (91, 73%). Over 50% (n=64) 'disagreed' or 'strongly disagreed' that obesity was a barrier.

Confidential: For Review Only

DISCUSSION

This national survey of health care practitioners from UK PICU's identified the importance of ERM as an intervention which participants believe can improve the physical, psychological and cognitive recovery of critically ill or injured infants and children across all ages. Our findings indicate support for ERM, but highlight uncertainty with suitability, variability with the definition of this complex intervention, variation in timing of initiating and which patient groups should receive ERM. Key barriers to ERM delivery were identified (e.g. funding and staffing) and potential clinical (e.g. improved psychological outcomes) and economic (e.g. reduced PICU length of stay) benefits to patients and PICUs.

How early is 'early' in ERM has been challenging to define for health care professionals. A time-based definition (e.g. within the first 2-5 days of ICU admission) has been proposed[15]; however, this can conflict with the patient stability approach (e.g. start as early as the patient is able to receive it). Our results indicate uncertainty and wide variation in time to start ERM (24hrs to over 7 days), increasing agreement for ERM to be considered after longer periods on PICU, and support for the concept of 'as early as the patient's clinical condition allows' which may be much longer. In the UK, only 43% of patients remain in PICU for 3 days or more[1]. The balance of delivering a programme of ERM to a large number of

patients who may only receive intervention within a very short period versus targeting patients at higher risk of prolonged PICU stay needs to be considered. However, it has been proposed that for ERM to become embedded in clinical practice, it should become a standard of care within 48hrs of ICU admission[16].

The uncertainty of the content of ERM also adds to the challenge for health care professionals to appreciate when ERM could be delivered. Understandably, normal bedside nursing care (e.g. functional positioning) may be considered acceptable earlier than more advanced physical therapies requiring multiple staff (e.g. sitting a ventilated child out of bed or in-bed cycling). Our survey identified that clinical stability as the most influential patient factor for initiation. The reported lack of ERM protocols in most (21/26) UK PICUs reinforces a strong requirement for evidence-based standardised protocols with optimal timing, intensity, frequency and duration of ERM. There is a need for flexible protocols to allow for tailoring rather than prescription.

ERM was more likely to be delivered to patients admitted for greater than 28 days, among patients with acquired brain injury or severe developmental delay across all age ranges. This reflects the cross-over between ERM and established rehabilitation programmes following acquired brain injury[17] or for patients admitted to PICU with a pre-existing package of rehabilitation. PICUs may be able to build on the success of established programmes to implement ERM to a wider critical care population and to use the existing multi-disciplinary expertise, and evolving rehabilitation evidence base to support the adoption of effective treatments. Most published ERM intervention studies to date have excluded patients less than three years of age[4 6]. However, this represents 60% of UK PICU patient population[1], and this age group was as likely to receive ERM as older children in our study. Future ERM

Confidential: For Review Only

trials should include all PICU age groups to ensure ERM content and efficacy is assessed across all potential patients.

Our results show that within the UK NHS setting, doctors, physiotherapists, and nurses have an equally important role in the decision to initiate ERM. This contrasts with other countries and health care settings where doctor-led approval was required by 70-81% of respondents and a potential barrier to mobilisation[18 19]. Nurse's and parent's roles are also important both in the initiation and delivery of ERM. In settings where nurses reported a low level (39%) of support for ERM, lack of involvement and understanding were key features[20]. In our study, 91% felt 'involved' in delivery of ERM. However, health care professionals reported that parents were the least likely group to initiate ERM (19%), although becoming influential in its ongoing delivery. This is consistent with a recent European point prevalence study in PICUs, highlighting the important role parents played in delivering ERM[21]. Building on both the multi-disciplinary support for ERM and empowering parents to initiate ERM may be potential strategies to improve implementation.

The key barriers to ERM practice were: i) at institutional level: insufficient resources, equipment and funding; ii) at provider level: limited staffing, training, protocols and slow recognition of readiness for ERM; and iii) at patient-level: physiological instability**,** risk of endotracheal tube dislodgement and amount of sedation. These barriers have been previously described in other health settings[18-20 22] with proposed facilitators, including adopting formal protocol/manual[23] (a key feature lacking across most UK PICUs), local champions[23] and team engagement / collaboration[23 24]. Implementation of ERM within the NHS will require building on the established multi-disciplinary teams, incorporating ERM within established protocols for weaning of mechanical ventilation and sedation and

Confidential: For Review Only

realistic goal setting. The potential benefits of ERM in terms of PICU length of stay and improvement in the psychological outcome for patients should be assessed within the core outcomes evaluating the efficacy of ERM within PICU.

The strength of this survey was an inclusive representation of 90% of UK PICUs and views from the wider multi-disciplinary team. A limitation was the use of a non-validated questionnaire. None or partial responses may indicate poor engagement in ERM topic, and as with all self-reported surveys, responses indicate reported rather than necessarily actual clinical practice. Finally, the findings represent the views of UK NHS staff and may not be generalisable to other healthcare settings.

CONCLUSION

ERM, in some form, is currently delivered to critically ill or injured patients of all ages across UK PICUs, but significant barriers to full implementation exist due to resource limitations and lack of institutional and national guidance. The UK should build on the existing strong multi-disciplinary support for ERM in PICUs. A standardised protocol that sets out defined ERM interventions and implementation support to tackle modifiable barriers is required to ensure the delivery of high-quality ERM.

Confidential: For Review Only

Acknowledgements This study/project is funded by the National Institute for Health Research (NIHR), project reference: 17/21/ HTA (Health Technology Assessment). The views expressed are those of the author(s) and not necessarily those of the NIHR or the Department of Health and Social Care.

We thank the Paediatric Intensive Care Society Study Group (PICS-SG), all members of the Paediatric Intensive Care Units and Principal Investigators for their contribution to the development of the PERMIT survey.

- Addenbrooke's Cambridge, Dr Nazima Pathan
- Alder Hey Children's NHS Foundation Trust, Liverpool, Dr Petr Jirasek
- Birmingham Women & Children's Hospital NHS Foundation Trust, Dr Barnaby Scholefield
- Bristol Royal Hospital for Children, Kate Baptiste
- Evelina London Children's Hospital, Dr Miriam Fine-Goulden
- Freeman Hospital, Newcastle upon Tyne Hospitals NHS Foundation Trust, Dr Deborah Cross
- GOSH CICU, Great Ormond Street Hospital for Children NHS Foundation Trust, Emma Shkurka
- GOSH PICU, Great Ormond Street Hospital for Children NHS Foundation Trust, Dr Peter Sidgwick
- Great North Children's Hospital, Newcastle, Dr Rachel Agbeko
- Kings College Hospital NHS Foundation Trust, Dr Bogdana Zoica
- Leeds Teaching Hospitals NHS Trust, St. James's University Hospital, Dr Sian Cooper
- Noah's Ark Children's Hospital for Wales, Dr Siva Oruganti
- Nottingham Children's Hospital, Queen's Medical Centre, Dr Georgina Harlow
- Oxford University Hospitals, Dr Deirdre O'Shea
- Royal Belfast Hospital for sick children, located in Royal Victoria Hospital, Dr Julie Richardson
- Royal Hospital for Children Glasgow, Dr Richard Levin
- Royal Hospital for Sick Children in Edinburgh, Dr Tsz Yan and Dr Milly Lo
- Royal Manchester Children's Hospital, Dr Ravishankar Nagaraj
- Sheffield Children's Hospital NHS Foundation Trust, Dr Rum Thomas
- Southampton Children's Hospital, Katy Morton
- St Georges Hospital, London, Dr Soumendu Manna
- St Mary's Hospital, Praed Street, Imperial London, Dr Tom Bycroft
- University Hospital Leicester, Dr Sanjiv Nichani
- Glenfield Hospital, Dr Sanjiv Nichani
- University Hospital North Midlands, Dr Kanaris Constantinos
- South Tees Hospitals NHS Foundation Trust

Contributions

BRS, JCMen, JCMan, RF, TR, NP, SB, JM, DM, MG, GC, KM, RP, RF, SL, and FK developed the original study protocol. BRS, EB, JCMan and FR developed the pilot survey and preliminary analysis. JT, JCMen, JCMan and BRS developed the final survey, coordinated administration, interpretation and analysis of data and BRS and JT drafted the manuscript, which has had input from all authors. All authors revised and approved the final manuscript. Paediatric

Confidential: For Review Only

Intensive Care Society Study-Group (PICS-SG) members reviewed the protocol and endorsed the survey.

Competing interests: Dr Julie C. Menzies is funded as a National Institute for Health Research (NIHR) 70@70 Senior Nurse and Midwife Research Leader. Dr Joseph C. Manning is an NIHR Health Education England funded ICA Clinical Lecturer. Dr Barnaby Scholefield receives funding as an NIHR Clinician Scientist Fellow, and Dr Forsyth and Prof Rapley receives NIHR grant funding. The views expressed in this article are those of the authors and not necessarily those of the NIHR, or the Department of Health and Social Care.

Funding informtion: This study/project is funded by the National Institute for Health Research (NIHR) [Health Technology Assessment Programme (project reference: 17/21)/ HTA]. The views expressed are those of the author(s) and not necessarily those of the NIHR or the Department of Health and Social Care.

Patient consent for publication: Not required.

Ethics approval: The study received ethical approval granted by the University of Birmingham review panel on 05/02/2019

Confidential: For Review Only

What is already known?

- Early rehabilitation and mobilisation (ERM) interventions are safe, feasible and effective within adult intensive care, but the evidence base in paediatric setting is limited.
- In critically ill adults, ERM delivery is tailored according to patient's cardiovascular support requirements, levels of consciousness and tolerance levels.
- Barriers to implementation of ERM in paediatric intensive care units (PICUs) have been described in North America but little is known about these within the UK NHS setting.

What does this study add?

- Despite positivity towards the concept of ERM, less than 20% of UK PICUs currently have an established ERM protocol to define ERM content and practice.
- ERM initiation and delivery is collaborative, but there is wide variability on which patients can receive ERM and when this should be initiated.
- The provision of a standardised protocol which sets out safe and defined ERM activities along with implementation support would tackle modifiable barriers of intervention delivery
- The most important barriers to ERM delivery in PICUs within the NHS are financial resource and staffing, lack of protocols for patient selection and ERM guidelines.

Confidential: For Review Only

References

1. PICANet. Paediatric Intensive Care Audit Network National Annual Report 2016 - 2018. 2019. https://www.picanet.org.uk/annual-reporting-and-publications/ (accessed 15th September 2020).

2. Lopes-Junior LC, Rosa M, Lima RAG. Psychological and Psychiatric Outcomes Following PICU Admission: A Systematic Review of Cohort Studies. Pediatr Crit Care Med 2018;19(1):e58-e67. doi: 10.1097/pcc.0000000000001390

3. Manning JC, Pinto NP, Rennick JE, et al. Conceptualizing Post Intensive Care Syndrome in Children-The PICS-p Framework. Pediatr Crit Care Med 2018;19(4):298-300. doi: 10.1097/pcc.0000000000001476

4. Choong K, Awladthani S, Khawaji A, et al. Early Exercise in Critically Ill Youth and Children, a Preliminary Evaluation: The wEECYCLE Pilot Trial. Pediatr Crit Care Med 2017;18(11):e546-e54. doi: 10.1097/pcc.0000000000001329

5. Pun BT, Balas MC, Barnes-Daly MA, et al. Caring for Critically Ill Patients with the ABCDEF Bundle: Results of the ICU Liberation Collaborative in Over 15,000 Adults. Crit Care Med 2019;47(1):3-14. doi: 10.1097/ccm.0000000000003482

6. Fink EL, Beers SR, Houtrow AJ, et al. Early Protocolized Versus Usual Care Rehabilitation for Pediatric Neurocritical Care Patients: A Randomized Controlled Trial. Pediatr Crit Care Med 2019 doi: 10.1097/pcc.0000000000001881

7. Nydahl P, Sricharoenchai T, Chandra S, et al. Safety of Patient Mobilization and Rehabilitation in the Intensive Care Unit. Systematic Review with Meta-Analysis. Ann Am Thorac Soc 2017;14(5):766-77. doi: 10.1513/AnnalsATS.201611-843SR

8. National Institute for Health and Care Excellence. Rehabilitation after critical illness in adults [NICE guidance CG83]. 2009 25 March 2009. https://www.nice.org.uk/guidance/CG83 (accessed 15th September 2020).

9. Wieczorek B, Burke C, Al-Harbi A, et al. Early mobilization in the pediatric intensive care unit: a systematic review. Journal of pediatric intensive care 2015;2015:129-70. doi: 10.1055/s-0035-1563386

10. Cuello-Garcia CA, Mai SHC, Simpson R, et al. Early Mobilization in Critically Ill Children: A Systematic Review. The Journal of pediatrics 2018;203:25-33.e6. doi: 10.1016/j.jpeds.2018.07.037

11. Mayring P. Qualitative Content Analysis. 2000 2000;1(2) doi: 10.17169/fqs-1.2.1089 [published Online First: 2000-06-30]

Confidential: For Review Only

12. Bengtsson M. How to plan and perform a qualitative study using content analysis. NursingPlus Open 2016;2:8-14. doi: https://doi.org/10.1016/j.npls.2016.01.001

13. Vaismoradi M, Turunen H, Bondas T. Content analysis and thematic analysis: Implications for conducting a qualitative descriptive study. Nurs Health Sci 2013;15(3):398-405. doi: 10.1111/nhs.12048

14. Burnard P. A method of analysing interview transcripts in qualitative research. Nurse Education Today 1991;11(6):461-66. doi: https://doi.org/10.1016/0260-6917(91)90009-Y

15. Hodgson CL, Berney S, Harrold M, et al. Clinical review: Early patient mobilization in the ICU. Critical Care 2013;17(1):207. doi: 10.1186/cc11820

16. Cameron S, Ball I, Cepinskas G, et al. Early mobilization in the critical care unit: A review of adult and pediatric literature. J Crit Care 2015;30(4):664-72. doi: 10.1016/j.jcrc.2015.03.032

17. Hayes L, Shaw S, Pearce MS, et al. Requirements for and current provision of rehabilitation services for children after severe acquired brain injury in the UK: a population-based study. Archives of Disease in Childhood 2017;102(9):813. doi: 10.1136/archdischild-2016-312166

18. Anekwe DE, Koo KK, de Marchie M, et al. Interprofessional Survey of Perceived Barriers and Facilitators to Early Mobilization of Critically Ill Patients in Montreal, Canada. J Intensive Care Med 2017:885066617696846. doi: 10.1177/0885066617696846

19. Choong K, Koo KK, Clark H, et al. Early mobilization in critically ill children: a survey of Canadian practice. Critical care medicine 2013;41(7):1745-53. doi: 10.1097/CCM.0b013e318287f592

20. Wang J, Xiao Q, Zhang C, et al. Intensive care unit nurses' knowledge, attitudes, and perceived barriers regarding early mobilization of patients. Nurs Crit Care 2020 doi: 10.1111/nicc.12507

21. Ista E, Scholefield BR, Manning JC, et al. Mobilization practices in critically ill children: a European point prevalence study (EU PARK-PICU). Critical Care 2020;24(1):1-11.

22. Fontela PC, Forgiarini LA, Jr., Friedman G. Clinical attitudes and perceived barriers to early mobilization of critically ill patients in adult intensive care units. Rev Bras Ter Intensiva 2018;30(2):187-94. doi: 10.5935/0103-507x.20180037

Confidential: For Review Only

23. Goddard SL, Lorencatto F, Koo E, et al. Barriers and facilitators to early rehabilitation in mechanically ventilated patients-a theory-driven interview study. J Intensive Care 2018;6:4. doi: 10.1186/s40560-018-0273-0

24. Zheng K, Sarti A, Boles S, et al. Impressions of Early Mobilization of Critically Ill Children-Clinician, Patient, and Family Perspectives. Pediatric critical care medicine : a journal of the Society of Critical Care Medicine and the World Federation of Pediatric Intensive and Critical Care Societies 2018;19(7):e350-e57. doi: 10.1097/pcc.0000000000001547 [published Online First: 2018/04/13]

Confidential: For Review Only

Table 1: Descriptions of ERM

Category	Sub-categories	Examples:
**Activity-focused**<br>ERM is activity-focused, with consideration of seating and equipment, primarily delivered by nursing and allied health care professionals with optimised sedation management to promote patient engagement.<br>There is a lack of consensus on what is routine care compared with purposeful ERM activity.	Activity – mobilising, positioning, stretching	"ERM provided includes - passive movements of limbs, periodic change of position, use of splints on extremities and if stable in the long term patients - sitting up in tumble form chair and mobilising out of bed." (Doctor, 67)<br><br>"We aim to get the children sitting upright, either over the edge of the bed or inappropriate seating as soon as possible." (Occupational Therapist, 001)<br><br>"Nursing staff are taught the importance of regular position changes for patient ... and positioning to maintain ranges of movement and prevent foot drop." (Nurse, 017)
	Core health care professional involvement – physiotherapist, nurse, occupational therapist	"We try and mobilise patients as soon as able when not invasively ventilated - physio led." (Doctor, 058)<br><br>"As physiotherapists, we work with the occupational therapist and nursing staff to either re-position, sit up in bed, sit on the edge of the bed or stand whilst on the ventilators."(Physiotherapist, 069)
	Additional health care professional involvement – psychology, dietician, play therapist, speech and language therapists	"Speech and language therapy become involved usually when nursing or medical staff identify a need for referral." (Doctor, 003)<br><br>"We have a Play Specialist on PICU, who assists with communication tools/toys." (Nurse, 082)
	Seating and equipment	"Specialist seating need – linking in with OT to ensure appropriate seating available." (Physiotherapist, 019)

Confidential: For Review Only

		"We have also in the past asked adult [services] to use their moto-med bike and used this with teenagers, but this can be a challenge as it is very far from PICU and it is often in use." (Physiotherapist, 014)
	Sedation management	"Working with the medics to wean sedation as quickly as possible. This promotes faster ability to mobilise and progress in their rehabilitation." (Nurse, 034)
	'Routine care' vs 'purposeful ERM package'	Some centres define ERM as passive range of movement, repositioning or providing splints. We would deem this as essential core cares rather than ERM; the large majority of our patients will have positioning and movement plan from day 1 of admission." (Physiotherapist, 111)
**Tailored** Following the assessment of needs and patient and family preference, ERM activities are personalised to the individual patient.	Assessment of need	"We have a programme where patients are categorised to one of 3 levels. Each level provides nursing and therapy staff with activities aligned to the acuity of the patient." (Nurse, 040)
	Individual preference	"Parents will tell us what their child enjoys doing and make suggestions, often provide toys/games from home." (Nurse, 076)<br><br>"Discuss with parents what patient enjoys doing, watching etc. Discuss options regarding taking patients 'out' where possible if long term patients." (Nurse, 015)
**Promote recovery** The purpose of ERM is to normalise the PICU environment, to create an environment that addresses holistic needs, sustains or promotes development and supports recovery from critical illness.	Normalising PICU environment	"Provide activities they would use for their enjoyment. This enables them to be more relaxed and less aware of what is going on with / around them." (Play specialist, 102)<br><br>"Being in PICU can be a frightening experience. Not only is a child/young person away from home, but also away from their usual environment, family and friends. There are unfamiliar sounds, smells, equipment and people. The play specialist can help to make the stay much more enjoyable and children/young people to cope and understand." (Play specialist, 055)
	Sustain / promote child development	"Encouraging the patient to regain or further their development in a therapeutic fun manner." (Play therapist,102)

Confidential: For Review Only

	Restoration / recovery	"Interventions aim to promote physical recovery – movement, and ability to engage in activities and psychological recovery – orientation, speech, ability to play and attend school. " (Nurse, 021)
**Timing of ERM** The optimal timing of ERM initiation is challenging for health care professionals to define, although likelihood perceived to increase with increased length of stay. Influenced by the perceived stability of patients and the balance of risk to benefit.	'Early' poorly defined	"I like to think we consider it within 2-3 days we have a better idea of the patients PICU journey and how long they are going to stay. The reality is it's usually longer... usually week 2 of admission if they are still on the unit." (Physiotherapist, 090)<br><br>"There is no formal plan for who decides if a patient should start ERM and when… Currently the delivery of ERM is fairly ad hoc, based on what is highlighted from daily handover each morning." (Physiotherapist, 083)<br><br>"We provide ERM as a structured programme… every patient after 24 hours of admission is considered for ERM." (Doctor, 012)
	Patient stability, risk and benefit	"Members of the PICU team with less experience of early rehab can deem ERM 'unsafe' which can occasionally act as a barrier."(Physiotherapist, 016)<br><br>"Usually ERM activity is not considered until patients can physiologically tolerate movement and are stable with observations." (Nurse, 008)
	'Long-stay'	"ERM is often thought about as a patient comes up to extubation or has failed extubation, and we feel that the reason for failure may be because of critical care weakness." (Physiotherapist, 031)<br><br>"We provide very little ERM on PICU, other than for long term patients, which is often, in my opinion, delayed." (Nurse, 007)

Confidential: For Review Only

Table 2: Content of ERM and non-ERM protocols

Items	Within an ERM protocol (n=12 respondents) yes n (%)	Within a non-ERM protocols (n=124 respondents) Yes n (%)
Physical therapy requiring additional equipment	9 (75)	18 (15)
Occupational therapy interventions	9 (75)	18 (15)
Physical therapy not requiring additional equipment	8 (67)	17 (14)
Speech and language therapy interventions	4 (33)	12 (10)
Psychology interventions	0 (0)	8 (6)
Delirium screening	0 (0)	1 (1)

Table 3: Reported frequency of receiving or being involved in Early Rehabilitation and Mobilisation by age groups, length of PICU stay, diagnostic category, and health care professional or parental/family role (n=124 respondents)*

By age group	Always (n, %)	Very often (n, %)	Sometimes (n, %)	Seldom (n, %)	Never (n, %)	Don't know (n, %)	Not Applicable[#] n
All PICU Patients (any age)	6 (5)	45 (36)	48 (39)	14 (11)	0 (0)	11 (9)	0
>10-18 years old	18 (15)	40 (32)	43 (35)	8 (6)	1 (1)	14 (11)	0
>4-10 years old	16 (13)	42 (34)	41 (33)	10 (8)	1 (1)	14 (11)	0
1-4 years old	13 (10)	42 (34)	39 (31)	13 (10)	1 (1)	16 (13)	0
Infants & <1 year old	6 (5)	47 (38)	32 (26)	20 (16)	3 (2)	16 (13)	0
**By length of stay in PICU**							
PICU >28 days	43 (35)	48 (39)	14 (11)	4 (3)	0 (0)	15 (12)	0
PICU >7-28 days	27 (22)	46 (37)	26 (21)	8 (6)	0 (0)	17 (14)	0
PICU 3-7 days	12 (10)	32 (26)	40 (32)	19 (15)	5 (4)	16 (13)	0
PICU <3 days	3 (2)	14 (11)	39 (31)	33 (27)	17 (14)	18 (15)	0

Confidential: For Review Only

**By diagnosistic category**							
Acquired brain injury	36 (29)	39 (31)	16 (13)	11 (9)	4 (3)	18 (15)	0
Severe developmental delay	11 (9)	43 (35)	39 (31)	12 (10)	3 (2)	16 (13)	0
Cancer	11 (9)	24 (19)	36 (29)	14 (11)	4 (3)	35 (28)	0
Pre-existing physical morbidity	9 (7)	38 (31)	44 (35)	12 (10)	4 (3)	17 (14)	0
Mechanically ventilated	9 (7)	38 (31)	39 (31)	18 (15)	5 (4)	15 (12)	0
Congenital heart disease	9 (7)	24 (19)	39 (31)	15 (12)	6 (5)	31 (25)	0
Respiratory illness	7 (6)	35 (28)	39 (31)	22 (18)	3 (2)	18 (15)	0
Sepsis	7 (6)	28 (23)	44 (35)	20 (16)	5 (4)	20 (16)	0
Multi-organ failure	5 (4)	14 (11)	32 (26)	35 (28)	12 (10)	26 (21)	0
Mechanically supported (eg extracorporeal life support)	4 (3)	8 (6)	14 (11)	13 (10)	47 (38)	38 (31)	0
**Health Care Professional team members & parent or family member(s) involvement in ERM (when applicable#)**							
Physiotherapist	86 (70)	27 (22)	4 (3)	2 (2)	0 (0)	4 (3)	1
Nurse	54 (44)	49 (40)	9 (7)	5 (4)	0 (0)	6 (5)	1
Parent or family member	36 (29)	56 (46)	19 (15)	7 (6)	1 (1)	4 (3)	1
Occupational therapist	23 (19)	34 (29)	29 (24)	20 (17)	6 (5)	7 (6)	5
Doctor	22 (18)	20 (16)	26 (21)	30 (24)	13 (11)	12 (10)	1
Play therapist	12 (10)	24 (21)	39 (34)	26 (23)	9 (8)	5 (4)	9
Dietician	10 (8)	20 (17)	12 (10)	29 (24)	37 (31)	13 (11)	3
Speech and Language therapist	4 (3)	11 (9)	40 (33)	35 (29)	20 (17)	10 (8)	4
Psychologist	3 (3)	11 (10)	24 (23)	32 (30)	30 (28)	6 (6)	18

* Percentages may not total 100 due to rounding

# Not applicable (e.g. not available in PICU) responses excluded from percent calculation.

Confidential: For Review Only

Figure 1: Perceived Benefits of Early Rehabilitation and Mobilisation
Ranking of participants potential top 5 perceived benefits of delivering ERM within PICU's. Sum of rank score: Ranking of top 5 (1-5) (1st placed rank scored 5 points to 5th placed scored 1 point). 121/124 (98%) participants ranked scores.

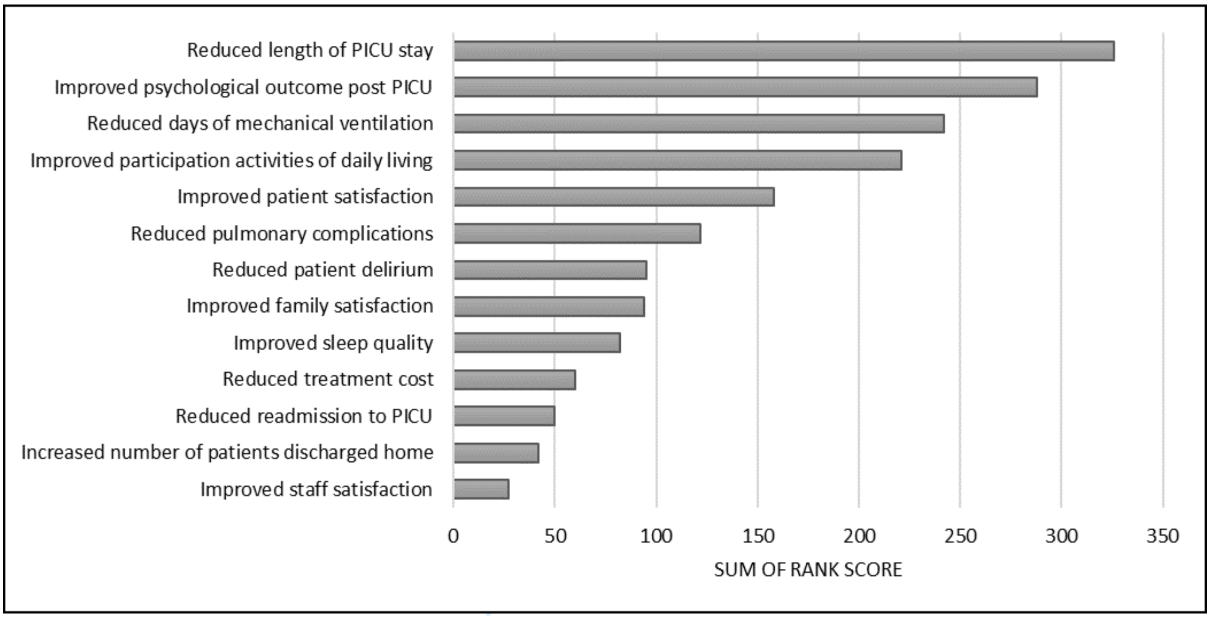

For Review Only

Figure 2: Perceived Barriers of Early Rehabilitation and Mobilisation
Institutional, Patients and Provider barriers to ERM. Figure 2 shows the percentage of responses for categories strongly agree, agree, neutral, disagree and strongly disagree. Responses ranked on the cumulative score of percentage 'strongly agree and agree'.

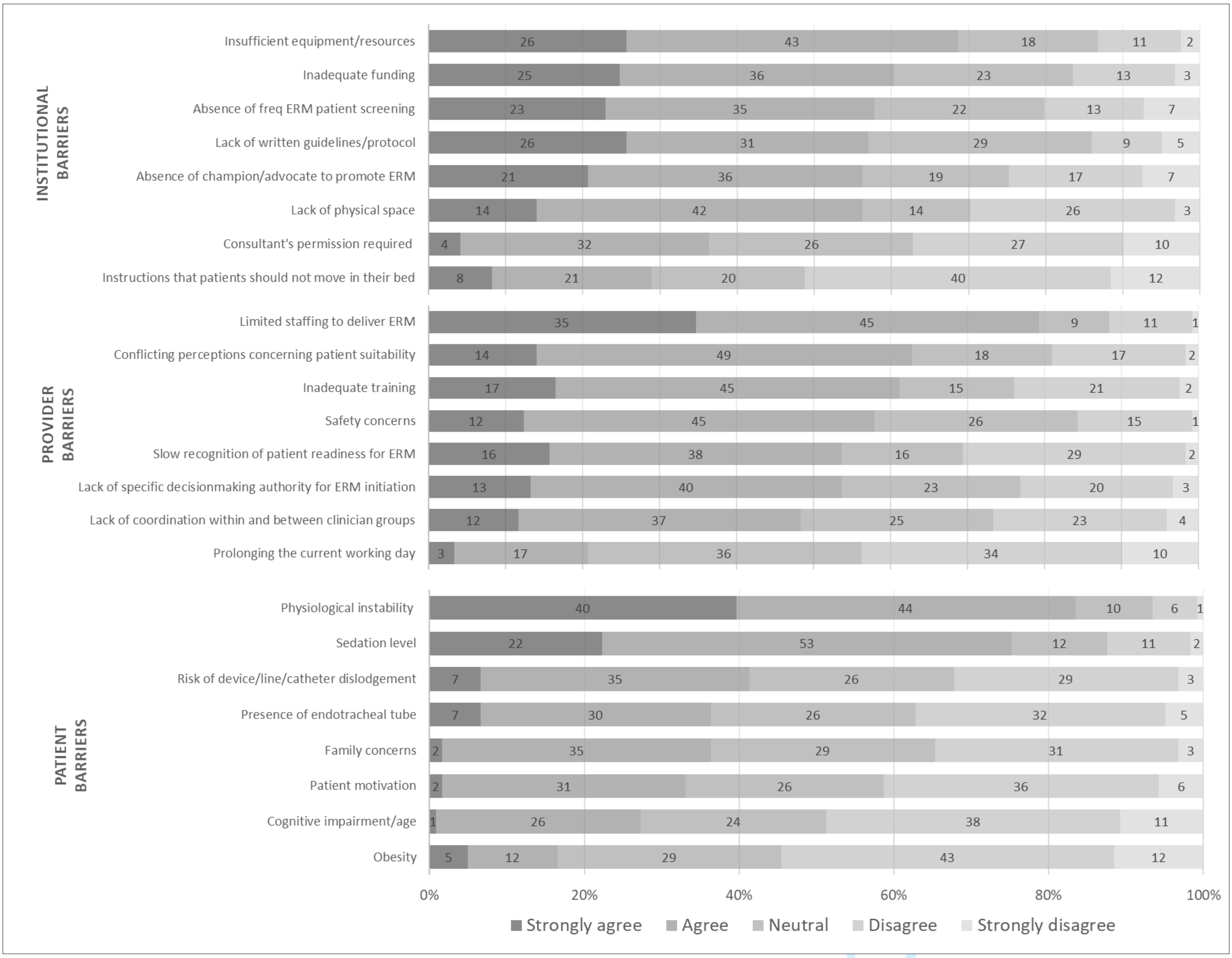

Supplemental Material 1

PERMIT Online Survey

Paediatric Early Rehabilitation and Mobilisation during InTensive care (PERMIT) Survey

1. Introduction

Thank you for your interest in completing a questionnaire as part of the PERMIT Study.

Why have I been invited to take part?
You have been invited to take part because you are a health care practitioner responsible for the delivery of care to children in a PICU. We would like the views of a wide range of health care professionals, and you have been identified either through the collaborating Paediatric Intensive Care Society, Physiotherapy and Occupational therapy membership lists or recommended by lead clinicians in your institutions.

Study Overview
We are conducting research into Early Rehabilitation and Mobilisation (ERM) during paediatric critical illness in the UK and assessing the feasibility and design of a future clinical trial. ERM can encompass patient-tailored interventions or bundles of care provided within the critical care unit. Rehabilitation has been shown to improve quality of life and patient outcomes in the adult ICU; however, there is limited evidence in the PIC patient population and setting.

The whole PERMIT programme aims to assess key questions regarding the acceptability and feasibility of ERM in PIC and form recommendations for a future clinical trial to establish its effectiveness.

In this questionnaire, we would like to find out:
- What forms of ERM are provided in your PICs and to which patients;
- What resources are available (or could be available) to deliver ERM;
- Your views on evaluating ERM interventions;
- Whether you would be interested in a future clinical trial to assess ERM.

Thank you for your time (which we calculated should be less than 20 minutes) in completing this questionnaire.

Barney Scholefield & Jacqueline Thompson

Confidential: For Review Only

On behalf of the PERMIT study team and the Paediatric Intensive Care Society -Study Group (PICS-SG)

2. A little demographic information about you please

1. Which hospital do you work at? *

Other (if not listed)

2. Which best reflects your current professional position? *

- [ ] Medical
- [ ] Nurse
- [ ] Physiotherapist
- [ ] Occupational therapist
- [ ] Speech and Language therapist
- [ ] Dietician
- [ ] Play therapist
- [ ] Psychologist
- [ ] Other (please specify):

3. Number of years experience working in PICU? *

- [ ] <1 year
- [ ] 1 to <5 years
- [ ] 5 to <10 years
- [ ] 10 to <15 years
- [ ] 15 to <20 years

Confidential: For Review Only

- [ ] More than 20 years
- [ ] I do not work in PICU

Comments:

3. What ERM do you currently provide in your PICU?

In the following questions, we hope to explore your current practice related to early rehabilitation and mobilisation (ERM). We understand that the term ERM may mean different things to different professional groups; however, we are keen to capture the current diverse views and how ERM is defined by you and your PICU team.

4. Please describe in the box below the current ERM practice within your PICU. (We are especially interested in whether you provide anything that you feel fits within the term ERM, who receives ERM, what specific actions are involved in ERM and who delivers the ERM).

5. Which of the following best describes when you would consider ERM for a patient? *

- [ ] Within 24 hours of admission to PICU
- [ ] About 2 to 3 days after PICU admission
- [ ] Around the end of the first week of PICU admission
- [ ] Before discharge from PICU
- [ ] As soon as the patient is stable enough
- [ ] As soon as the patient is off mechanical ventilatory support
- [ ] To start earlier than we historically considered starting rehabilitation and mobilisation
- [ ] We do not consider ERM for our PICU patients
- [ ] I don't know
- [ ] Other (please specify):

Confidential: For Review Only

Comments:

6. Which team members are involved in ERM in your PICU? (Please select how frequently they are involved. If the team member is not available in your PICU please select 'Not applicable')

	Always	Very often	Sometimes	Seldom	Never	Not applicable (as team member is not available in my PICU)	Don't know
Medical staff	☐	☐	☐	☐	☐	☐	☐
Nursing staff	☐	☐	☐	☐	☐	☐	☐
Speech and language therapist	☐	☐	☐	☐	☐	☐	☐
Physiotherapist	☐	☐	☐	☐	☐	☐	☐
Occupational therapist	☐	☐	☐	☐	☐	☐	☐
Psychologist	☐	☐	☐	☐	☐	☐	☐
Play therapist	☐	☐	☐	☐	☐	☐	☐
Dietician	☐	☐	☐	☐	☐	☐	☐
Patients family or carers	☐	☐	☐	☐	☐	☐	☐
Other	☐	☐	☐	☐	☐	☐	☐

Please define 'other' or add any comments

Confidential: For Review Only

7. How often do patients, in general, receive ERM in your PICU?

	Always	Very often	Sometimes	Seldom	Never	Don't know
All PICU patients	☐	☐	☐	☐	☐	☐

Comments:

8. How often do patients in the following age groups receive ERM in your PICU? (Please select an option for each)

	Always	Very often	Sometimes	Seldom	Never	Don't know
Infants & <1 year old	☐	☐	☐	☐	☐	☐
1-4 years old	☐	☐	☐	☐	☐	☐
> 4-10 years old	☐	☐	☐	☐	☐	☐
> 10-18 years old	☐	☐	☐	☐	☐	☐

Comments:

9. How often do patients within the following diagnostic categories receive ERM in your PICU? (Please select an option for each)

	Always	Very often	Sometimes	Seldom	Never	Don't know
Acquired brain injury patients (e.g. TBI, meningitis)	☐	☐	☐	☐	☐	☐
Sepsis	☐	☐	☐	☐	☐	☐
Congenital heart disease	☐	☐	☐	☐	☐	☐
Cancer	☐	☐	☐	☐	☐	☐
Mechanically ventilated	☐	☐	☐	☐	☐	☐

Confidential: For Review Only

	Always	Very often	Sometimes	Seldom	Never	Don't know
Mechanically supported (e.g. ECMO)	☐	☐	☐	☐	☐	☐
Respiratory illness (bronchiolitis or pneumonia)	☐	☐	☐	☐	☐	☐
Multi-organ failure	☐	☐	☐	☐	☐	☐
With pre-existing physical co-morbidity	☐	☐	☐	☐	☐	☐
With pre-existing severe developmental delay	☐	☐	☐	☐	☐	☐

Comments:

10. How often do patients, with the following length of stay, receive ERM in your PICU? (Please select an option for each)

	Always	Very often	Sometimes	Seldom	Never	Don't know
Patients in PICU <3 days	☐	☐	☐	☐	☐	☐
Patients in PICU 3-7 days	☐	☐	☐	☐	☐	☐
Patients in PICU >7-28 days	☐	☐	☐	☐	☐	☐
Patients in PICU >28 days	☐	☐	☐	☐	☐	☐

Comments:

11. Who decides if the patient can start to receive ERM? (Please select all that apply)

☐ Medical consultant

☐ Other member of the medical team (e.g. PICU trainee/ advanced nurse practitioner)

https://mc.manuscriptcentral.com/bmjpo

Confidential: For Review Only

- [ ] Bedside nurse
- [ ] Senior nurse on shift
- [ ] Physiotherapist
- [ ] Occupational therapist
- [ ] Parent(s)
- [ ] Don't know
- [ ] Not applicable
- [ ] Other (please specify):

Comments:

12. How does your PICU assess whether patients tolerate and cope with the therapy provided? (eg monitor safety)

13. Do you currently use or have an established guideline or protocol regarding management of ERM in your PICU?

- [ ] No
- [ ] Yes - if you are able to share, please email to b.scholefield@bham.ac.uk
- [ ] Don't know

Comments:

4. Question regarding your established ERM programme or protocol

14. What do you have within your protocolised ERM treatment? (Please select all that apply)

Confidential: For Review Only

- [ ] Physical therapy not requiring additional equipment
- [ ] Physical therapy requiring additional equipment
- [ ] Occupational therapy
- [ ] Speech and language therapy
- [ ] Psychology
- [ ] Delirium screening
- [ ] We do not have a programme or protocol related to ERM activities
- [ ] Other (please specify):

Comments:

15. What are the key factors in successfully implementing your ERM protocol?

5. ERM related processes and equipment in your PICU

The following three questions are related to additional processes or equipment in your PICU related to ERM activities.

16. Does your PICU have a protocol or guidelines for the management of the following?

	Yes	No	Don't know
Sedation	☐	☐	☐
Analgesia	☐	☐	☐
Drug/ opioid withdrawal	☐	☐	☐
Delirium	☐	☐	☐

Confidential: For Review Only

	Yes	No	Don't know
Mobilisation (at any time point)	☐	☐	☐

17. What types of therapies or activities are available in your PICU? (Please select all that apply)

- ☐ Physical therapy - not requiring additional equipment (e.g. passive or active movements)
- ☐ Physical therapy - requiring additional equipment (e.g. in bed cycling)
- ☐ Occupational therapy
- ☐ Speech and language therapy
- ☐ Clinical psychology
- ☐ Delirium screening
- ☐ Patient diaries
- ☐ Play therapy
- ☐ Pet therapy
- ☐ Music therapy
- ☐ Other (please specify):

18. What equipment does your PICU have available for physical therapy/mobilisation? (Please select all that apply)

- ☐ Tilt table
- ☐ Bed with Trendelenburg features
- ☐ Bed with full chair position
- ☐ Bed with chair egress - exit out the foot of the bed
- ☐ Speciality bed with continuous side to side rotation
- ☐ Bed with retractable footboard i.e. flex a foot feature

Confidential: For Review Only

- [ ] Mobile lifts
- [ ] Ceiling lifts
- [ ] Portable ventilators
- [ ] Patient rolling walker
- [ ] Bedside cycle (patient can remain in bed)
- [ ] Transcutaneous electrical stimulation of muscles
- [ ] Specialist wheelchair
- [ ] Specialist static seating i.e. tumble forms
- [ ] Other (please specify):

6. Preferences, barriers and outcomes

For the following questions, we would explore your strength of opinion on ERM as a therapy, potential barriers to using ERM in practice and which are the most important clinical outcomes to consider when assessing if ERM is beneficial.

19. Please select one option regarding your current views of ERM in your PICU? *

- [ ] Crucial, should be the top priority in the care of PICU patients
- [ ] Very important, should be a priority in the care of PICU patients
- [ ] Important, should be a priority in the care of PICU patients
- [ ] Somewhat important, should be considered in the care of PICU patients
- [ ] Not of great importance, clinicians should bear it in mind in the care of PICU patients
- [ ] Of minimal importance to the care of PICU patients
- [ ] Of no importance to the care of PICU patients

Comments:

Confidential: For Review Only

20. Below are potential institutional barriers to the delivery of ERM. Please select to what extent you agree/disagree that each are barriers to delivery of ERM in your PICU. *

	Strongly Agree	Agree	Neutral	Disagree	Strongly Disagree
Instructions that patients should not move in their bed	☐	☐	☐	☐	☐
Consultant's permission or prescription is required prior to mobilisation	☐	☐	☐	☐	☐
Insufficient equipment/ resources	☐	☐	☐	☐	☐
Inadequate funding	☐	☐	☐	☐	☐
Lack of written guidelines/ protocol	☐	☐	☐	☐	☐
Lack of physical space	☐	☐	☐	☐	☐
Absence of champion/ advocate to promote ERM	☐	☐	☐	☐	☐
Absence of frequent (e.g. daily) ERM patient screening	☐	☐	☐	☐	☐

Comments:

21. Below are potential patient barriers to the delivery of ERM. Please select to what extent you agree/disagree that each are barriers to delivery of ERM in your PICU. *

	Strongly Agree	Agree	Neutral	Disagree	Strongly Disagree
Physiological instability ('the patient is just too sick')	☐	☐	☐	☐	☐

Confidential: For Review Only

	Strongly Agree	Agree	Neutral	Disagree	Strongly Disagree
Sedation level	☐	☐	☐	☐	☐
Presence of endotracheal intubation	☐	☐	☐	☐	☐
Risk of device/ line/ catheter dislodgement	☐	☐	☐	☐	☐
Cognitive impairment/ age	☐	☐	☐	☐	☐
Obesity	☐	☐	☐	☐	☐
Patient motivation	☐	☐	☐	☐	☐
Family concerns	☐	☐	☐	☐	☐

Comments:

22. Below are potential provider barriers to the delivery of ERM. Please select to what extent you agree/disagree that each are barriers to delivery of ERM in your PICU. *

	Strongly Agree	Agree	Neutral	Disagree	Strongly Disagree
Lack of prioritisation of ERM within patient care plans	☐	☐	☐	☐	☐
Limited staffing to deliver ERM	☐	☐	☐	☐	☐
Lack of coordination within and between clinician groups	☐	☐	☐	☐	☐
Slow recognition of patient readiness for ERM	☐	☐	☐	☐	☐
Conflicting perceptions concerning patient suitability	☐	☐	☐	☐	☐

Confidential: For Review Only

	Strongly Agree	Agree	Neutral	Disagree	Strongly Disagree
Safety concerns	☐	☐	☐	☐	☐
Lack of specific decision-making authority for ERM initiation	☐	☐	☐	☐	☐
Inadequate training	☐	☐	☐	☐	☐
Prolonging the current working day	☐	☐	☐	☐	☐

Comments:

23. Please rank what you perceive to be the top 5 most important benefits that ERM could provide in PICU. (Please select, in order, the top perceived benefits by selecting a box). *

	1 (most important)	2	3	4	5 (least important)
Reduction in treatment cost	☐	☐	☐	☐	☐
Reduction in patient delirium	☐	☐	☐	☐	☐
Reduction in length of ICU stay	☐	☐	☐	☐	☐
Reduction in days requiring mechanical ventilation	☐	☐	☐	☐	☐
Reduction in the rate of pulmonary complications	☐	☐	☐	☐	☐
Reduction in readmission (hospital and PICU)	☐	☐	☐	☐	☐
Increase in number of patients discharged home	☐	☐	☐	☐	☐
Improved patient satisfaction	☐	☐	☐	☐	☐

Confidential: For Review Only

	1 (most important)	2	3	4	5 (least important)
Improved staff satisfaction	☐	☐	☐	☐	☐
Improved family satisfaction	☐	☐	☐	☐	☐
Improvement in patient sleep quality	☐	☐	☐	☐	☐
Improvement in psychological impact of PICU care	☐	☐	☐	☐	☐
Improvement in daily life participation following discharge	☐	☐	☐	☐	☐

Comments:

7. Final thoughts

24. Would you be willing to support a future clinical trial in the UK and Ireland based on evaluating ERM practice?

☐ Yes

☐ No

☐ Don't Know

Comments:

25. Would you allow us to contact you in the future with information regarding workshops we will be running to discuss ERM practice and trial feasibility, please? *

☐ Yes (please click the link on the next page to provide best contact information, thank you!)

☐ No

Confidential: For Review Only

Funding statement: This study/project is funded by the National Institute for Health Research (NIHR) [Health Technology Assessment Programme (project reference: 17/21)/HTA]. The views expressed are those of the author(s) and not necessarily those of the NIHR or the Department of Health and Social Care.

Confidential: For Review Only

Supplemental Tables

Supplemental Table 1: Characteristics of survey respondents (n=124 respondents)

Professional group	n (%)*
Nurse	34 (27)
Physiotherapist	28 (23)
Medical doctor (consultant)	22 (18)
Occupational therapist	19 (15)
Play therapist	7 (6)
Psychologist	7 (6)
Dietician	6 (5)
Speech and Language therapist	1 (1)
**Years of experience**	**n (%)***
<1 year	7 (6)
1 to <5 years	27 (22)
5 to <10 years	30 (24)
10 to <15 years	14 (11)
15 to <20 years	33 (27)
More than 20 years	15 (12)

* Percentages may not total 100 due to rounding

Supplemental Table 2: Current views of ERM in PICU (n=121 respondents)

Current view of ERM in PICU	n (%)*
Crucial, should be the top priority in the care of PICU patients	15 (12)
Very important, should be a priority in the care of PICU patients	67 (55)
Important, should be a priority in the care of PICU patients	35 (29)
Somewhat important, should be considered in the care of PICU patients	4 (3)
Not of great importance, clinicians should bear it in mind in the care of PICU patients	0 (0)
Of minimal importance to the care of PICU patients	0 (0)
Of no importance to the care of the PICU patients	0 (0)

* Percentages may not total 100 due to rounding

Confidential: For Review Only

Supplemental Table 3: Top 5 most important benefits of Early Rehabilitation and Mobilisation

Perceived ERM benefits (Most to least important)	Sum Rank Score*
Reduction in length of ICU stay	326
Improvement in the psychological impact of PICU care	288
Reduction in days requiring MV	242
Improvement in daily life participation following discharge	221
Improved patient satisfaction	158
Reduction in the rate of pulmonary complications	122
Reduction in patient delirium	95
Improved family satisfaction	94
Improvement in patient sleep quality	82
Reduction in treatment cost	60
Reduction in readmission	50
Increase in the number of patients discharged home	42
Improved staff satisfaction	27

*Sum of rank score: Ranking of top 5 (1-5) (1st placed rank scored 5 points to 5th placed scored 1 point). 121/124 (98%) participants ranked scores.

Supplemental Table 4: Which professional or parent groups in PICU intiates ERM (n=124 respondents)

Professional or family group	Yes n (%)
Physiotherapist	96 (77)
Physicians	92 (74)
Bedside nurse	64 (52)
Senior nurse	58 (47)
Other members of the medical team	55 (44)
Occupational therapist	37 (30)
Parent or family member	24 (19)

Confidential: For Review Only

Supplemental Table 5: Barriers of ERM delivery within Paediatric Intensive Care Units (n=124 respondents)*

**Institutional barriers**	**Strongly Agree n (%)**	**Agree n (%)**	**Neutral n (%)**	**Disagree n (%)**	**Strongly Disagree n (%)**	**Skipped responses[#]** (n)
Insufficient equipment/resources	31 (26)	52 (43)	22 (18)	13 (11)	3 (2)	3
Lack of written guidelines/protocol	31 (26)	38 (31)	35 (29)	11 (9)	6 (5)	3
Inadequate funding	30 (25)	43 (36)	28 (23)	16 (13)	4 (3)	3
Absence of champion/advocate to promote ERM	25 (21)	43 (36)	23 (19)	21 (17)	9 (7)	3
Absence of frequent ERM patient screening	25 (23)	38 (35)	24 (22)	14 (13)	8 (7)	14
Lack of physical space	17 (14)	51 (42)	17 (14)	32 (26)	4 (3)	3
Instructions that patients should not move in their bed	10 (8)	25 (21)	24 (20)	48 (40)	14 (12)	3
Consultant's permission or prescription is required prior to mobilisation	5 (4)	39 (32)	32 (26)	33 (27)	12 (10)	3
**Provider barriers**						
Slow recognition of patient readiness for ERM	19 (16)	46 (38)	19 (16)	35 (29)	2 (2)	3
Limited staffing to deliver ERM	42 (35)	54 (45)	11 (9)	13 (11)	1 (1)	3
Lack of prioritisation of ERM within patient care plans	27 (22)	45 (37)	22 (18)	24 (20)	3 (2)	3
Inadequate training	20 (17)	54 (45)	18 (15)	26 (21)	3 (2)	3
Conflicting perceptions concerning patient suitability	17 (14)	59 (49)	22 (18)	21 (17)	2 (2)	3
Lack of specific decision-making authority for ERM initiation	16 (13)	49 (40)	28 (23)	24 (20)	4 (3)	3
Safety concerns	15 (12)	55 (45)	32 (26)	18 (15)	1 (1)	3
Lack of coordination within and between clinician groups	14 (12)	44 (37)	30 (25)	27 (23)	5 (4)	4
Prolonging the current working day	4 (3)	21 (17)	43 (36)	41 (34)	12 (10)	3
**Patients barriers**						
Levels of physiological instability	48 (40)	53 (44)	12 (10)	7 (6)	1 (1)	3
Sedation level	27 (22)	64 (53)	15 (12)	13 (11)	2 (2)	3
Presence of endotracheal intubation levels	8 (7)	36 (30)	32 (26)	39 (32)	6 (5)	3
Risk of device/line/catheter dislodgement levels	8 (7)	42 (35)	32 (26)	35 (29)	4 (3)	3
Cognitive impairment/age levels	1 (1)	32 (26)	29 (24)	46 (38)	13 (11)	3
Obesity levels	6 (5)	14 (12)	35 (29)	52 (43)	14 (12)	3
Patient motivation level	2 (2)	38 (31)	31 (26)	43 (36)	7 (6)	3
Family concerns levels	2 (2)	42 (35)	35 (29)	38 (31)	4 (3)	3

Confidential: For Review Only

***** Percentages may not total 100 due to rounding [#] Skipped responses excluded from percent calculation.

Confidential: For Review Only

Supplemental Table 6: Types of ERM equipment available in each Paediatric Intensive Care Unit (n=26 PICUs)

ERM equipment available in each PICU	n (%)
Specialist static seating	25 (96)
Portable ventilators	23 (88)
Mobile lifts	22 (85)
Tilt table	22 (85)
Bed with full chair position	18 (69)
Specialist wheelchair	18 (69)
Bed with Trendelenburg features	13 (50)
Patient rolling walker	11 (42)
Bedside cycle or inbed cycle	10 (38)
Ceiling lifts	8 (31)
Speciality bed with continuous side to side rotation	8 (31)
Bed with retractable footboard	7 (27)
Bed with chair egress exit out the foot of the bed	5 (19)
Transcutaneous Electrical Nerve stimulation	3 (12)

Confidential: For Review Only

BMJ Paediatrics Open

Early Mobilisation and Rehabilitation in the PICU: A UK survey

Journal:	*BMJ Paediatrics Open*
Manuscript ID	bmjpo-2021-001300.R1
Article Type:	Original research
Date Submitted by the Author:	01-Dec-2021
Complete List of Authors:	Thompson, Jacqueline; University of Birmingham, ; University of Birmingham<br>Menzies, Julie; Birmingham Women's and Children's NHS Foundation Trust, Paediatric Intensive Care<br>Manning, Joseph ; Nottingham University Hospitals NHS Trust, Nottingham Children's Hospital; University of Nottingham, Children and Young people Health Research, School of Health Sciences<br>McAnuff, Jennifer; Newcastle University, Population Health Sciences Institute, Newcastle University; Northumbria University, Department of Social Work, Education and Community Wellbeing<br>Brush, Emily; University of Birmingham<br>Ryde, Francesca; University of Birmingham<br>Rapley, Tim; Northumbria University<br>Pathan, Nazima; University of Cambridge, Paediatrics<br>Brett, Stephen; Imperial College of Science Technology and Medicine, Department of surgery and cancer<br>Moore, David; University of Birmingham<br>Geary, Michelle; University Hospital Southampton NHS Foundation Trust, Child Health<br>Colville, Gillian; St George's University Hospitals NHS Foundation Trust, Paediatric Psychology Service<br>Morris, Kevin; Birmingham Women's & Children's NHS Foundation Trust<br>PARSLOW, ROGER; UNIVERSITY OF LEEDS, PAEDIATRIC EPIDEMIOLOGY GROUP<br>Feltbower, Richard; UNIVERSITY OF LEEDS<br>Lockley, Sophie; Birmingham Children's Hospital<br>Kirkham, Fenella; Neurosciences Unit, UCL Institute of Child Health<br>Forsyth, Rob; Newcastle University, Institute of Neuroscience<br>Scholefield, Barnaby; University of Birmingham
Keywords:	Rehabilitation, Epidemiology

SCHOLARONE™
Manuscripts

I, the Submitting Author has the right to grant and does grant on behalf of all authors of the Work (as defined in the below author licence), an exclusive licence and/or a non-exclusive licence for contributions from authors who are: i) UK Crown employees; ii) where BMJ has agreed a CC-BY licence shall apply, and/or iii) in accordance with the terms applicable for US Federal Government officers or employees acting as part of their official duties; on a worldwide, perpetual, irrevocable, royalty-free basis to BMJ Publishing Group Ltd ("BMJ") its licensees and where the relevant Journal is co-owned by BMJ to the co-owners of the Journal, to publish the Work in this journal and any other BMJ products and to exploit all rights, as set out in our licence.

The Submitting Author accepts and understands that any supply made under these terms is made by BMJ to the Submitting Author unless you are acting as an employee on behalf of your employer or a postgraduate student of an affiliated institution which is paying any applicable article publishing charge ("APC") for Open Access articles. Where the Submitting Author wishes to make the Work available on an Open Access basis (and intends to pay the relevant APC), the terms of reuse of such Open Access shall be governed by a Creative Commons licence – details of these licences and which Creative Commons licence will apply to this Work are set out in our licence referred to above.

Other than as permitted in any relevant BMJ Author's Self Archiving Policies, I confirm this Work has not been accepted for publication elsewhere, is not being considered for publication elsewhere and does not duplicate material already published. I confirm all authors consent to publication of this Work and authorise the granting of this licence.

Confidential: For Review Only

Early Mobilisation and Rehabilitation in the PICU: A UK survey

Manuscript type: Original Research

Authors:

Thompson JY[1], Menzies JC[2], Manning JC[3,4], McAnuff J[5, 6], Brush E[1], Ryde F[1], Rapley TR[6], Pathan N[7], Brett S[8], Moore D[9], Geary M[10], Colville G[11], Morris KP[2], Parslow RC[12], Feltbower RG[12], Lockley S[13], Kirkham F[10], Forsyth R[14], Scholefield B R[1,2], On behalf of the PERMIT Collaborators & Paediatric Intensive Care Society Study-Group (PICS-SG)

Author affiliations:

1. Birmingham Acute Care Research Group, Institute of Inflammation and Ageing, University of Birmingham
2. Paediatric Intensive Care, Birmingham Women's & Children's NHS Foundation Trust
3. Nottingham Children's Hospital, Nottingham University Hospitals NHS Trust
4. Children and Young People Health Research, School of Health Sciences, The University of Nottingham
5. Population Health Sciences Institute, Newcastle University
6. Department of Social Work, Education and Community Wellbeing, Northumbria University
7. Department of Paediatrics, The Chancellor, Masters, and Scholars of the University of Cambridge
8. Department of surgery and cancer; Imperial College of Science, Technology and Medicine

Confidential: For Review Only

9. Institute of Applied Health, University of Birmingham

10. Child Health, University Hospital Southampton NHS Foundation Trust

11. Paediatric Psychology Service; St George's University Hospitals, NHS Foundation Trust

12. Leeds Institute for Data Analytics, School of Medicine, University of Leeds, Leeds

13. PPI Representative

14. Translational and Clinical Research Institute, Newcastle University

Corresponding author:

Barnaby R Scholefield; B.Scholefield@bham.ac.uk

Twitter handle: @BarneyUoB

Abstract word count: 264 words now

Main text word count (excluding abstract, reference list, tables and figures): 2735

References: 24

Tables: 2

Figures: 2

Supplementary files: 3 (1 survey, 6 supplemental tables)

Confidential: For Review Only

Abstract

Objective: To understand the context and professional perspectives of delivering early rehabilitation and mobilisation (ERM) within UK Paediatric Intensive Care Units (PICU).

Design: A web-based survey administered May-August 2019.

Setting: United Kingdom PICUs.

Participants: A total of 124 staff from 26 PICUs participated, including 22 (18%) Doctors, 34 (27%) Nurses, 28 (23%) Physiotherapists, 19 (15%) Occupational therapists, and 21 (17%) were other professionals.

Results: Key components of participants' definitions of ERM included tailored, multi-disciplinary rehabilitation packages focused on promoting recovery. Multi-disciplinary involvement in initiating ERM was commonly reported. Over half of respondents favoured delivering ERM after achieving physiological stability (n=69, 56%). All age groups were considered for ERM by relevant health professionals. However, responses differed concerning the timing of initiation. Interventions considered for ERM were more likely to be delivered to patients when PICU length of stay exceeded 28 days and among patients with acquired brain injury or severe developmental delay. The most commonly identified barriers were: physiological instability (81%), limited staffing (79%), sedation requirement (73%), insufficient resources and equipment (69%), lack of recognition of patient readiness (67%), patient suitability (63%), inadequate training (61%), and inadequate funding (60%). Respondents ranked reduction in PICU length of stay 74% and improvement in psychological outcomes 73% as the most important benefits of ERM.

Confidential: For Review Only

Conclusion: ERM is gaining familiarity and endorsement in UK PICUs, but significant barriers to implementation due to limited resources and variation in content and delivery of ERM persist. A standardised protocol that sets out defined ERM interventions, along with implementation support to tackle modifiable barriers, is required to ensure the delivery of high-quality ERM.

Confidential: For Review Only

INTRODUCTION

In the UK, approximately 20,000 children are admitted to Paediatric Intensive Care Units (PICU) yearly[1], and although most recover, some develop longer-term physical, psychological and cognitive impairment[2]. These significant morbidities have been termed Post Intensive Care Syndrome in Paediatrics (PICS-p)[3]. Early rehabilitation and mobilisation (ERM) encompass patient-tailored interventions, delivered within seven days of admission, individually[4] or in a bundled package[5] to patients within intensive care settings. It is provided by health professionals from multiple disciplines and care-givers and may promote physical (e.g. functional activities)[4] and non-physical (e.g. psychological, cognitive)[6] recovery.

Within adult ICUs, ERM has been demonstrated to be safe, feasible and cost-effective[7 8]. It can shorten the length of ventilation, duration of intensive care and hospital stay with economic benefit, improve long term physical functioning and return to independence, and is recommended by National Institute for Health Care Excellence (NICE)[8]. Whilst ERM has been reported to be safe and feasible in PICUs [9 10], the patient population is different, and ERM is less well defined, leading to variability in practice. This variation in practice and components of interventions delivered has also been described in European PICU's[11]. At present, no national standardised care pathway for ERM exists within UK PICUs.

This study forms part of the National Institute for Health Research (NIHR) funded PERMIT study (NIHR HTA: 17/21/06) investigating ERM in children. This work set out to understand reported practices and perceptions of ERM within the UK PICU context.

Confidential: For Review Only

Our objectives were to:

I. Explore how health care professionals describe and administer ERM using a qualitative approach
II. Identify and describe current ERM practice using a quantitative approach

Understand and quantify perceived barriers and facilitators of ERM, presenting findings using descriptive analysis.METHODS

A web-based survey (administered through www.smartsurvey.co.uk) was developed that included 25 questions (Supplementary material file 1). A patient representative was involved in the design and development of the survey. A pilot survey was conducted among multi-disciplinary health professionals (n=40) teams within two PICUs to assess acceptability and comprehensiveness. There were very few missing responses; therefore, no questions were removed, but five questions were rephrased to add clarity. Pilot responses were excluded from the main survey analysis.

A UK Paediatric Intensive Care Society Study Group (PICS-SG) member of each UK PICU (n=29) was contacted via email and requested to identify and cascade to members of their local multi-disciplinary team (including at least one physiotherapist, doctor and nurse) to complete the survey. Participating PICUs were sent a survey link between May and August 2019 to distribute. Three follow up reminders were sent at weekly intervals to non-responders.

Likert scales with the following categories (and associated scores) – always (5), very often (4), sometimes (3), seldom (2) and never (1) - were used to express the frequency of

Confidential: For Review Only

practice or level of agreement (using median and IQR of score). The ranking of perceived ERM benefits was calculated using the sum of ranked scores of respondents top 5 important benefits (5 points for 1st, reducing to 1 point for 5th placed). Statistical analysis was performed using R version x64 3.5.1 (R Foundation for Statistical Computing, Vienna, Austria).

Open-ended responses were analysed using a qualitative content analysis approach[12]. Two researchers independently familiarised themselves with the data and conducted open-coding, utilising NVIVO™ software for data management. Codes were then discussed, summarised and organised[13 14]. In relevant sections of the paper, free text quotes from respondents are reported to add context and clarity[15]. The University of Birmingham granted institutional ethical approval on 05/02/2019 (reference ERN_18-1134). Consent was implied through survey completion.

Patient and Public Involvement

The pilot survey received input from multi-disciplinary health professionals who were members of the UK Paediatric Intensive Care Society Study Group (PICS-SG). We also recruited a patient representative as part of the study team to ensure the perspective, experiences and preferences of children admitted to PICU were incorporated into the design and development of the survey. The patient representative was a parent of a child who had been admitted to PICU. They both contributed to the nature of questions considered in the survey to ensure the study outcome would be relevant to parents and members of the PICS-SG on dissemination.

Confidential: For Review Only

RESULTS

Demographics

We received responses from PICS-SG link members in 26/29 (90%) UK PICUs. A total of 191 healthcare professionals opened the survey link, with 124 (65%) submitting responses.

As shown in Supplemental Table 1, the majority of respondents were nurses (n=34, 27%), physiotherapists (n=28, 23%) and doctors (n=22, 18%). There were also responses from occupational therapists (n=19, 15%), play therapists (n=7, 6%), psychologists (n=7, 6%), dieticians (n=6, 5%), and speech and language therapists (n=1, 1%). Almost three-quarters of health professionals had ≥5 years experience, with 48 (39%) ≥15 years. The majority of respondents considered ERM to be a priority, either crucial 15 (12%), very important 67 (55%) or important 35 (29%) in the care of PICU patients (see Supplemental Table 2).

Description of ERM

We invited respondents to describe ERM on their terms. Descriptions were provided by 104 (84%), which were summarised into four categories, 'Activity-focused', 'Tailored', 'Promote recovery', and 'Timing of ERM' (see Table 1, Supplementary material 3). Overall, ERM was considered to be an individualised package of graded interventions based on an activity-focused programme, to reduce the sequelae of critical illness or injury. However, responses differed for when ERM should be initiated, often emphasising the need for individualisation.

Availability of established ERM Protocols

Confidential: For Review Only

Respondents were asked to describe the content of established ERM protocols within their PICU. Only 12 participants (10%) reported working in a PICU with an established ERM protocol (n=5/26, 19% of PICUs). The most common components of ERM protocols were 'physical therapy not requiring additional equipment' (n=9/12, 75%) and 'occupational therapy interventions' (n=8/12, 67%). Only 4/12 (33%) referred to play therapy or speech and language therapy, and no ERM protocol specified input from psychologists or psychiatrists. All participants were asked about the content of non-ERM protocols in their PICU. Only 18/124 (15%) reported that guidance for physical or occupational therapy activities existed in other non-ERM protocols within PICU (Table 1).

Recipients of ERM

51 (41%) respondents reported that *All PICU patients* 'always' or 'very often' received ERM (Table 2). Overall, 14 (11%) respondents reported 'seldom' or 'never' delivering ERM.

ERM was reported to be more likely to be delivered to patients when PICU length of stay exceeded 28 days. Patients admitted for 28 days or more were more likely (n=91, 75%) to 'always' or 'very often' receive ERM compared to those admitted for shorter periods. Only 17 (13%) of those staying fewer than three days, 44 (36%) of those admitted between three to seven days and 73 (59%) of those admitted between seven to 28 days were more likely to receive ERM. . Participants reported that patients with acquired brain injury (n=75, 60%) and severe developmental delay (n=54, 44%) were 'always' or 'very often' likely to receive ERM.

Perceived benefits of ERM

Confidential: For Review Only

Participants ranked the five most important potential benefits of ERM out of thirteen options (Figure 1 and Supplemental Table 3). The most important outcomes identified were: 1) reduced PICU length of stay, 2) improved psychological outcomes for patients after PICU, 3) reduced days of mechanical ventilation, 4) improved participation in activities of daily living and 5) improved patient satisfaction.

Initiation and delivery of ERM

The decision for ERM initiation was perceived by 96 (77%) to be primarily led by physiotherapists, doctors (n=92, 74%), and bedside nurses (n=64, 52%). Parents were felt to initiate ERM by only (n=24, 19%) of respondents (Supplemental Table 4).

The most influential factor in ERM initiation was reported to be patient stability (n=69, 56%). Other influential factors were the length of stay; 15 (12%) reported ERM was initiated within 24 hours, and 16 (13%) within 2-3 days of PICU admission. Only 5 (4%) of respondents would not consider ERM at all on PICU. The influence of perceived clinical stability is demonstrated in respondents' free-text comments:

> "We are involved as early as required depending on the child/young person medical stability and their rehabilitation needs." (Occupational Therapist, 033)

> "Usually ERM activity is not considered until patients can physiologically tolerate movement and are cardiovascularly stable." (Nurse, 008)

Assessment of patient stability and tolerance of ERM was less well described. Most respondents (n=98, 79%) provided subjective cues or informal clinical criteria. These

Confidential: For Review Only

included monitoring vital signs, physiological changes, observation of behavioural changes and documentation of adverse events.

Physiotherapists (n=113, 92%), nurses (n=103, 84%), and parents or family members (n=92, 75%) were 'always' or 'very often' involved in the ongoing delivery of ERM with less frequent input from other members of the multi-disciplinary teams (Table 2).

Barriers to ERM Implementation

Figure 2 presents the perceived barriers of ERM (see also Supplemental Table 5). The most significant factors identified as barriers at the institutional levels were insufficient resources and equipment ('strongly agree' or 'agree': 83, 69%) and inadequate funding (73, 61%). Participants provided examples of resources having to be shared across organisations or specially ordered to deliver ERM to patients.

> "All equipment shared with the whole therapy department at present, therefore dependent on availability." (OT, 010)

A lack of established protocols (n=69, 57%), ERM champions (n=68, 57%), space (n=68, 56%), and robust patient screening processes (n=63, 58%) were also issues identified by respondents. Most PICUs had access to standard lifting 22/26 (85%) and specialist static seating equipment 25/26 (96%); however, bedside or in-bed cycling machines were only available in 10 (38%) of PICUs (see Supplemental Table 6).

Limited staffing was the most frequently reported barrier to ERM being delivered by providers, with 101 (79%) 'agreeing' or 'strongly agreeing'. Approximately half of the

Confidential: For Review Only

respondents agreed that issues such as training, patient safety, lack of decision-making authority, and delays in recognising patients' ERM needs were barriers to ERM initiation. However, only 25 (21%) 'agreed' or 'strongly agreed' that the impact of ERM potentially prolonging the working day was a barrier.

The two most frequently reported barriers to delivering ERM at the patient level were physiological instability (n=101, 81% agreeing or strongly agreeing) and sedation (n=91, 73%). Over 50% (n=64) 'disagreed' or 'strongly disagreed' that obesity was a barrier.

DISCUSSION

This national survey of health care practitioners from UK PICU's identified the importance of ERM as an intervention which participants believe can improve the physical and psychological recovery of critically ill or injured infants and children across all ages. Our findings indicate support for ERM but highlight uncertainty with suitability, variability with the definition of this complex intervention, variation in the timing of initiation and which patient groups should receive ERM. Key barriers to ERM delivery were identified (e.g. funding and staffing) and potential clinical (e.g. improved psychological outcomes) and economic (e.g. reduced PICU length of stay) benefits to patients and PICUs.

How early is 'early' in ERM has been challenging to define for health care professionals? A time-based definition (e.g. within the first 2-5 days of ICU admission) has been proposed[16]; however, this can conflict with the patient stability approach (e.g. start as early as the patient is able to receive it). Our results indicate uncertainty and wide variation in time to

https://mc.manuscriptcentral.com/bmjpo
Confidential: For Review Only

start ERM (24hrs to over seven days), increasing agreement for ERM to be considered after longer periods on PICU, and support for the concept of 'as early as the patient's clinical condition allows' which may be much longer. In the UK, only 43% of patients remain in PICU for three days or more[1]. The balance of delivering a programme of ERM to a large number of patients who may only receive intervention within a very short period versus targeting patients at higher risk of prolonged PICU stay needs to be considered. However, it has been proposed that for ERM to become embedded in clinical practice, it should become a standard of care within 48hrs of ICU admission[17].

The uncertainty of the content of ERM also adds to the challenge for health care professionals to appreciate when ERM could be delivered. Understandably, routine bedside nursing care (e.g. functional positioning) may be considered acceptable earlier than more advanced physical therapies requiring multiple staff (e.g. sitting a ventilated child out of bed or in-bed cycling). Our survey identified that clinical stability as the most influential patient factor for initiation. The reported lack of ERM protocols in most (21/26) UK PICUs reinforces a strong requirement for evidence-based standardised protocols with optimal timing, intensity, frequency and duration of ERM. There is a need for flexible protocols to allow for tailoring rather than prescription.

ERM was more likely to be delivered to patients admitted for greater than 28 days among patients with acquired brain injury or severe developmental delay across all age ranges. This reflects the cross-over between ERM and established rehabilitation programmes following acquired brain injury[18] or for patients admitted to PICU with a pre-existing rehabilitation package. PICUs may be able to build on the success of established programmes to implement ERM to a wider critical care population and to use the existing multi-disciplinary

Confidential: For Review Only

expertise, and evolving rehabilitation evidence base to support the adoption of effective treatments. Most published ERM intervention studies to date have excluded patients less than three years of age[4 6]. However, this represents 60% of the UK PICU patient population[1], and this age group was as likely to receive ERM as older children in our study. Future ERM trials should include all PICU age groups to ensure ERM content and efficacy is assessed across all potential patients.

Our results show that doctors, physiotherapists, and nurses have an equally important role in the decision to initiate ERM within the UK NHS setting. This contrasts with other countries and health care settings where doctor-led approval was required by 70-81% of respondents, which was a potential barrier to mobilisation[19 20]. Nurse's and parent's roles are also important both in the initiation and delivery of ERM. In settings where nurses reported a low level (39%) of support for ERM, lack of involvement and understanding were key features[21]. In our study, 91% felt 'involved' in the delivery of ERM. However, health care professionals reported that parents were the least likely group to initiate ERM (19%), although becoming influential in its ongoing delivery. This is consistent with a recent European point prevalence study in PICUs, highlighting the important role parents played in delivering ERM[22]. Building on both the multi-disciplinary support for ERM and empowering parents to initiate ERM may be potential strategies to improve implementation.

The key barriers to ERM practice were: i) at institutional level: insufficient resources, equipment and funding; ii) at provider level: limited staffing, training, protocols and slow recognition of readiness for ERM; and iii) at patient-level: physiological instability**,** risk of endotracheal tube dislodgement and amount of sedation. These barriers have been previously described in other health settings[19-21 23] with proposed facilitators, including

https://mc.manuscriptcentral.com/bmjpo

Confidential: For Review Only

adopting formal protocol/manual[24] (a key feature lacking across most UK PICUs), local champions[24] and team engagement / collaboration[24 25]. Implementation of ERM within the NHS will require building on the established multi-disciplinary teams, incorporating ERM within established protocols for weaning of mechanical ventilation and sedation and realistic goal setting. The potential benefits of ERM in terms of PICU length of stay and improvement in the psychological outcome for patients should be assessed within the core outcomes evaluating the efficacy of ERM within PICU.

The strength of this survey was an inclusive representation of 90% of UK PICUs and views from the wider multi-disciplinary team. However, none or partial responses may indicate poor engagement in ERM topic, and as with all self-reported surveys, responses indicate reported rather than actual clinical practice. A limitation was the use of a non-validated questionnaire. We did not conduct questionnaire validation because it would be very difficult to standardize these questions within the time constraints of the study and the NIHR HTA funding. The study team considered the questionnaire suitable for the study aims after receiving two rounds of expert reviews. Finally, the findings represent the views of UK NHS staff and may not be generalisable to other healthcare settings.

Confidential: For Review Only

CONCLUSION

ERM, in some form, is currently delivered to critically ill or injured patients of all ages across UK PICUs, but significant barriers to full implementation exist due to resource limitations and lack of institutional and national guidance. The UK should build on the existing strong

multi-disciplinary support for ERM in PICUs. A standardised protocol that sets out defined ERM interventions and implementation support to tackle modifiable barriers is required to ensure the delivery of high-quality ERM.

Confidential: For Review Only

Acknowledgements This study/project is funded by the National Institute for Health Research (NIHR), project reference: 17/21/ HTA (Health Technology Assessment). The views expressed are those of the author(s) and not necessarily those of the NIHR or the Department of Health and Social Care.

We thank the Paediatric Intensive Care Society Study Group (PICS-SG), all members of the Paediatric Intensive Care Units and Principal Investigators for their contribution to the development of the PERMIT survey.

- Addenbrooke's Cambridge, Dr Nazima Pathan
- Alder Hey Children's NHS Foundation Trust, Liverpool, Dr Petr Jirasek
- Birmingham Women & Children's Hospital NHS Foundation Trust, Dr Barnaby Scholefield
- Bristol Royal Hospital for Children, Kate Baptiste
- Evelina London Children's Hospital, Dr Miriam Fine-Goulden
- Freeman Hospital, Newcastle upon Tyne Hospitals NHS Foundation Trust, Dr Deborah Cross
- GOSH CICU, Great Ormond Street Hospital for Children NHS Foundation Trust, Emma Shkurka
- GOSH PICU, Great Ormond Street Hospital for Children NHS Foundation Trust, Dr Peter Sidgwick
- Great North Children's Hospital, Newcastle, Dr Rachel Agbeko
- Kings College Hospital NHS Foundation Trust, Dr Bogdana Zoica
- Leeds Teaching Hospitals NHS Trust, St. James's University Hospital, Dr Sian Cooper
- Noah's Ark Children's Hospital for Wales, Dr Siva Oruganti
- Nottingham Children's Hospital, Queen's Medical Centre, Dr Georgina Harlow
- Oxford University Hospitals, Dr Deirdre O'Shea
- Royal Belfast Hospital for sick children, located in Royal Victoria Hospital, Dr Julie Richardson
- Royal Hospital for Children Glasgow, Dr Richard Levin
- Royal Hospital for Sick Children in Edinburgh, Dr Tsz Yan and Dr Milly Lo
- Royal Manchester Children's Hospital, Dr Ravishankar Nagaraj
- Sheffield Children's Hospital NHS Foundation Trust, Dr Rum Thomas
- Southampton Children's Hospital, Katy Morton
- St Georges Hospital, London, Dr Soumendu Manna
- St Mary's Hospital, Praed Street, Imperial London, Dr Tom Bycroft
- University Hospital Leicester, Dr Sanjiv Nichani
- Glenfield Hospital, Dr Sanjiv Nichani
- University Hospital North Midlands, Dr Kanaris Constantinos
- South Tees Hospitals NHS Foundation Trust

Contributions

BRS, JCMen, JCMan, RF, TR, NP, SB, JM, DM, MG, GC, KM, RP, RF, SL, and FK developed the original study protocol. BRS, EB, JCMan and FR developed the pilot survey and preliminary analysis. JT, JCMen, JCMan and BRS developed the final survey, coordinated administration, interpretation and analysis of data and BRS and JT drafted the manuscript, which has had input from all authors. All authors revised and approved the final manuscript. Paediatric

Confidential: For Review Only

Intensive Care Society Study-Group (PICS-SG) members reviewed the protocol and endorsed the survey.

Competing interests: Dr Julie C. Menzies is funded as a National Institute for Health Research (NIHR) 70@70 Senior Nurse and Midwife Research Leader. Dr Joseph C. Manning is an NIHR Health Education England funded ICA Clinical Lecturer. Dr Barnaby Scholefield receives funding as an NIHR Clinician Scientist Fellow, and Dr Forsyth and Prof Rapley receives NIHR grant funding. The views expressed in this article are those of the authors and not necessarily those of the NIHR, or the Department of Health and Social Care.

Funding informtion: This study/project is funded by the National Institute for Health Research (NIHR) [Health Technology Assessment Programme (project reference: 17/21)/ HTA]. The views expressed are those of the author(s) and not necessarily those of the NIHR or the Department of Health and Social Care.

Patient consent for publication: Not required.

Ethics approval: The study received ethical approval granted by the University of Birmingham review panel on 05/02/2019

Confidential: For Review Only

What is already known?

- Early rehabilitation and mobilisation (ERM) interventions are safe, feasible and effective within adult intensive care, but the evidence base in paediatric setting is limited.
- In critically ill adults, ERM delivery is tailored according to patient's cardiovascular support requirements, levels of consciousness and tolerance levels.
- Barriers to implementation of ERM in paediatric intensive care units (PICUs) have been described in North America but little is known about these within the UK NHS setting.

What does this study add?

- Despite positivity towards the concept of ERM, less than 20% of UK PICUs currently have an established ERM protocol to define ERM content and practice.
- ERM initiation and delivery is collaborative, but there is wide variability on which patients can receive ERM and when this should be initiated.
- The provision of a standardised protocol which sets out safe and defined ERM activities along with implementation support would tackle modifiable barriers of intervention delivery
- The most important barriers to ERM delivery in PICUs within the NHS are financial resource and staffing, lack of protocols for patient selection and ERM guidelines.

Confidential: For Review Only

References

1. PICANet. Paediatric Intensive Care Audit Network National Annual Report 2016 - 2018. 2019. https://www.picanet.org.uk/annual-reporting-and-publications/ (accessed 15th September 2020).

2. Lopes-Junior LC, Rosa M, Lima RAG. Psychological and Psychiatric Outcomes Following PICU Admission: A Systematic Review of Cohort Studies. Pediatr Crit Care Med 2018;19(1):e58-e67. doi: 10.1097/pcc.0000000000001390

3. Manning JC, Pinto NP, Rennick JE, et al. Conceptualizing Post Intensive Care Syndrome in Children-The PICS-p Framework. Pediatr Crit Care Med 2018;19(4):298-300. doi: 10.1097/pcc.0000000000001476

4. Choong K, Awladthani S, Khawaji A, et al. Early Exercise in Critically Ill Youth and Children, a Preliminary Evaluation: The wEECYCLE Pilot Trial. Pediatr Crit Care Med 2017;18(11):e546-e54. doi: 10.1097/pcc.0000000000001329

5. Pun BT, Balas MC, Barnes-Daly MA, et al. Caring for Critically Ill Patients with the ABCDEF Bundle: Results of the ICU Liberation Collaborative in Over 15,000 Adults. Crit Care Med 2019;47(1):3-14. doi: 10.1097/ccm.0000000000003482

6. Fink EL, Beers SR, Houtrow AJ, et al. Early Protocolized Versus Usual Care Rehabilitation for Pediatric Neurocritical Care Patients: A Randomized Controlled Trial. Pediatr Crit Care Med 2019 doi: 10.1097/pcc.0000000000001881

7. Nydahl P, Sricharoenchai T, Chandra S, et al. Safety of Patient Mobilization and Rehabilitation in the Intensive Care Unit. Systematic Review with Meta-Analysis. Ann Am Thorac Soc 2017;14(5):766-77. doi: 10.1513/AnnalsATS.201611-843SR

8. National Institute for Health and Care Excellence. Rehabilitation after critical illness in adults [NICE guidance CG83]. 2009 25 March 2009. https://www.nice.org.uk/guidance/CG83 (accessed 15th September 2020).

9. Wieczorek B, Burke C, Al-Harbi A, et al. Early mobilization in the pediatric intensive care unit: a systematic review. Journal of pediatric intensive care 2015;2015:129-70. doi: 10.1055/s-0035-1563386

10. Cuello-Garcia CA, Mai SHC, Simpson R, et al. Early Mobilization in Critically Ill Children: A Systematic Review. The Journal of pediatrics 2018;203:25-33.e6. doi: 10.1016/j.jpeds.2018.07.037

11. Mayring P. Qualitative Content Analysis. 2000 2000;1(2) doi: 10.17169/fqs-1.2.1089 [published Online First: 2000-06-30]

Confidential: For Review Only

12. Bengtsson M. How to plan and perform a qualitative study using content analysis. NursingPlus Open 2016;2:8-14. doi: https://doi.org/10.1016/j.npls.2016.01.001

13. Vaismoradi M, Turunen H, Bondas T. Content analysis and thematic analysis: Implications for conducting a qualitative descriptive study. Nurs Health Sci 2013;15(3):398-405. doi: 10.1111/nhs.12048

14. Burnard P. A method of analysing interview transcripts in qualitative research. Nurse Education Today 1991;11(6):461-66. doi: https://doi.org/10.1016/0260-6917(91)90009-Y

15. Hodgson CL, Berney S, Harrold M, et al. Clinical review: Early patient mobilizationin the ICU. Critical Care 2013;17(1):207. doi: 10.1186/cc11820

16. Cameron S, Ball I, Cepinskas G, et al. Early mobilizationin the critical care unit: A review of adult and pediatric literature. J Crit Care 2015;30(4):664-72. doi: 10.1016/j.jcrc.2015.03.032

17. Hayes L, Shaw S, Pearce MS, et al. Requirements for and current provision of rehabilitation services for children after severe acquired brain injury in the UK: a population-based study. Archives of Disease in Childhood 2017;102(9):813. doi: 10.1136/archdischild-2016-312166

18. Anekwe DE, Koo KK, de Marchie M, et al. Interprofessional Survey of Perceived Barriers and Facilitators to Early Mobilizationof Critically Ill Patients in Montreal, Canada. J Intensive Care Med 2017:885066617696846. doi: 10.1177/0885066617696846

19. Choong K, Koo KK, Clark H, et al. Early mobilization in critically ill children: a survey of Canadian practice. Critical care medicine 2013;41(7):1745-53. doi: 10.1097/CCM.0b013e318287f592

20. Wang J, Xiao Q, Zhang C, et al. Intensive care unit nurses' knowledge, attitudes, and perceived barriers regarding early mobilization of patients. Nurs Crit Care 2020 doi: 10.1111/nicc.12507

21. Ista E, Scholefield BR, Manning JC, et al. Mobilization practices in critically ill children: a European point prevalence study (EU PARK-PICU). Critical Care 2020;24(1):1-11.

22. Fontela PC, Forgiarini LA, Jr., Friedman G. Clinical attitudes and perceived barriers to early mobilization of critically ill patients in adult intensive care units. Rev Bras Ter Intensiva 2018;30(2):187-94. doi: 10.5935/0103-507x.20180037

Confidential: For Review Only

23. Goddard SL, Lorencatto F, Koo E, et al. Barriers and facilitators to early rehabilitation in mechanically ventilated patients-a theory-driven interview study. J Intensive Care 2018;6:4. doi: 10.1186/s40560-018-0273-0

24. Zheng K, Sarti A, Boles S, et al. Impressions of Early Mobilization of Critically Ill Children-Clinician, Patient, and Family Perspectives. Pediatric critical care medicine : a journal of the Society of Critical Care Medicine and the World Federation of Pediatric Intensive and Critical Care Societies 2018;19(7):e350-e57. doi: 10.1097/pcc.0000000000001547 [published Online First: 2018/04/13]

Confidential: For Review Only

Table 1: Content of ERM and non-ERM protocols

Items	Within an ERM protocol (n=12 respondents) Yes n (%)	Within a non-ERM protocols (n=124 respondents) Yes n (%)
Physical therapy requiring additional equipment	9 (75)	18 (15)
Occupational therapy interventions	9 (75)	18 (15)
Physical therapy not requiring additional equipment	8 (67)	17 (14)
Speech and language therapy interventions	4 (33)	12 (10)
Psychology interventions	0 (0)	8 (6)
Delirium screening	0 (0)	1 (1)

Table 2: Reported frequency of receiving or being involved in Early Rehabilitation and Mobilisation by age groups, length of PICU stay, diagnostic category, and health care professional or parental/family role (n=124 respondents)*

By age group	Always (n, %)	Very often (n, %)	Sometimes (n, %)	Seldom (n, %)	Never (n, %)	Don't know (n, %)	Not Applicable[#] n
All PICU Patients (any age)	6 (5)	45 (36)	48 (39)	14 (11)	0 (0)	11 (9)	0
>10-18 years old	18 (15)	40 (32)	43 (35)	8 (6)	1 (1)	14 (11)	0
>4-10 years old	16 (13)	42 (34)	41 (33)	10 (8)	1 (1)	14 (11)	0
1-4 years old	13 (10)	42 (34)	39 (31)	13 (10)	1 (1)	16 (13)	0
Infants & <1 year old	6 (5)	47 (38)	32 (26)	20 (16)	3 (2)	16 (13)	0
**By length of stay in PICU**							
PICU >28 days	43 (35)	48 (39)	14 (11)	4 (3)	0 (0)	15 (12)	0
PICU >7-28 days	27 (22)	46 (37)	26 (21)	8 (6)	0 (0)	17 (14)	0
PICU 3-7 days	12 (10)	32 (26)	40 (32)	19 (15)	5 (4)	16 (13)	0
PICU <3 days	3 (2)	14 (11)	39 (31)	33 (27)	17 (14)	18 (15)	0

Confidential: For Review Only

**By diagnosistic category**							
Acquired brain injury	36 (29)	39 (31)	16 (13)	11 (9)	4 (3)	18 (15)	0
Severe developmental delay	11 (9)	43 (35)	39 (31)	12 (10)	3 (2)	16 (13)	0
Cancer	11 (9)	24 (19)	36 (29)	14 (11)	4 (3)	35 (28)	0
Pre-existing physical morbidity	9 (7)	38 (31)	44 (35)	12 (10)	4 (3)	17 (14)	0
Mechanically ventilated	9 (7)	38 (31)	39 (31)	18 (15)	5 (4)	15 (12)	0
Congenital heart disease	9 (7)	24 (19)	39 (31)	15 (12)	6 (5)	31 (25)	0
Respiratory illness	7 (6)	35 (28)	39 (31)	22 (18)	3 (2)	18 (15)	0
Sepsis	7 (6)	28 (23)	44 (35)	20 (16)	5 (4)	20 (16)	0
Multi-organ failure	5 (4)	14 (11)	32 (26)	35 (28)	12 (10)	26 (21)	0
Mechanically supported (e.g. extracorporeal life support)	4 (3)	8 (6)	14 (11)	13 (10)	47 (38)	38 (31)	0
**Health Care Professional team members & parent or family member(s) involvement in ERM (when applicable#)**							
Physiotherapist	86 (70)	27 (22)	4 (3)	2 (2)	0 (0)	4 (3)	1
Nurse	54 (44)	49 (40)	9 (7)	5 (4)	0 (0)	6 (5)	1
Parent or family member	36 (29)	56 (46)	19 (15)	7 (6)	1 (1)	4 (3)	1
Occupational therapist	23 (19)	34 (29)	29 (24)	20 (17)	6 (5)	7 (6)	5
Doctor	22 (18)	20 (16)	26 (21)	30 (24)	13 (11)	12 (10)	1
Play therapist	12 (10)	24 (21)	39 (34)	26 (23)	9 (8)	5 (4)	9
Dietician	10 (8)	20 (17)	12 (10)	29 (24)	37 (31)	13 (11)	3
Speech and Language therapist	4 (3)	11 (9)	40 (33)	35 (29)	20 (17)	10 (8)	4
Psychologist	3 (3)	11 (10)	24 (23)	32 (30)	30 (28)	6 (6)	18

* Percentages may not total 100 due to rounding

# Not applicable (e.g. not available in PICU) responses excluded from percent calculation.

Confidential: For Review Only

Confidential: For Review Only

Supplemental Material 1

PERMIT Online Survey

Paediatric Early Rehabilitation and Mobilisation during InTensive care (PERMIT) Survey

1. Introduction

Thank you for your interest in completing a questionnaire as part of the PERMIT Study.

Why have I been invited to take part?
You have been invited to take part because you are a health care practitioner responsible for the delivery of care to children in a PICU. We would like the views of a wide range of health care professionals, and you have been identified either through the collaborating Paediatric Intensive Care Society, Physiotherapy and Occupational therapy membership lists or recommended by lead clinicians in your institutions.

Study Overview
We are conducting research into Early Rehabilitation and Mobilisation (ERM) during paediatric critical illness in the UK and assessing the feasibility and design of a future clinical trial. ERM can encompass patient-tailored interventions or bundles of care provided within the critical care unit. Rehabilitation has been shown to improve quality of life and patient outcomes in the adult ICU; however, there is limited evidence in the PIC patient population and setting.

The whole PERMIT programme aims to assess key questions regarding the acceptability and feasibility of ERM in PIC and form recommendations for a future clinical trial to establish its effectiveness.

In this questionnaire, we would like to find out:
- What forms of ERM are provided in your PICs and to which patients;
- What resources are available (or could be available) to deliver ERM;
- Your views on evaluating ERM interventions;
- Whether you would be interested in a future clinical trial to assess ERM.

Thank you for your time (which we calculated should be less than 20 minutes) in completing this questionnaire.

Barney Scholefield & Jacqueline Thompson

Confidential: For Review Only

On behalf of the PERMIT study team and the Paediatric Intensive Care Society -Study Group (PICS-SG)

2. A little demographic information about you please

1. Which hospital do you work at? *

Other (if not listed)

2. Which best reflects your current professional position? *

- [ ] Medical
- [ ] Nurse
- [ ] Physiotherapist
- [ ] Occupational therapist
- [ ] Speech and Language therapist
- [ ] Dietician
- [ ] Play therapist
- [ ] Psychologist
- [ ] Other (please specify):

3. Number of years experience working in PICU? *

- [ ] <1 year
- [ ] 1 to <5 years
- [ ] 5 to <10 years
- [ ] 10 to <15 years
- [ ] 15 to <20 years

Confidential: For Review Only

☐ More than 20 years

☐ I do not work in PICU

Comments:

3. What ERM do you currently provide in your PICU?

In the following questions, we hope to explore your current practice related to early rehabilitation and mobilisation (ERM). We understand that the term ERM may mean different things to different professional groups; however, we are keen to capture the current diverse views and how ERM is defined by you and your PICU team.

4. Please describe in the box below the current ERM practice within your PICU. (We are especially interested in whether you provide anything that you feel fits within the term ERM, who receives ERM, what specific actions are involved in ERM and who delivers the ERM).

5. Which of the following best describes when you would consider ERM for a patient? *

☐ Within 24 hours of admission to PICU

☐ About 2 to 3 days after PICU admission

☐ Around the end of the first week of PICU admission

☐ Before discharge from PICU

☐ As soon as the patient is stable enough

☐ As soon as the patient is off mechanical ventilatory support

☐ To start earlier than we historically considered starting rehabilitation and mobilisation

☐ We do not consider ERM for our PICU patients

☐ I don't know

☐ Other (please specify):

Confidential: For Review Only

Comments:

6. Which team members are involved in ERM in your PICU? (Please select how frequently they are involved. If the team member is not available in your PICU please select 'Not applicable')

	Always	Very often	Sometimes	Seldom	Never	Not applicable (as team member is not available in my PICU)	Don't know
Medical staff	☐	☐	☐	☐	☐	☐	☐
Nursing staff	☐	☐	☐	☐	☐	☐	☐
Speech and language therapist	☐	☐	☐	☐	☐	☐	☐
Physiotherapist	☐	☐	☐	☐	☐	☐	☐
Occupational therapist	☐	☐	☐	☐	☐	☐	☐
Psychologist	☐	☐	☐	☐	☐	☐	☐
Play therapist	☐	☐	☐	☐	☐	☐	☐
Dietician	☐	☐	☐	☐	☐	☐	☐
Patients family or carers	☐	☐	☐	☐	☐	☐	☐
Other	☐	☐	☐	☐	☐	☐	☐

Please define 'other' or add any comments

Confidential: For Review Only

7. How often do patients, in general, receive ERM in your PICU?

	Always	Very often	Sometimes	Seldom	Never	Don't know
All PICU patients	☐	☐	☐	☐	☐	☐

Comments:

8. How often do patients in the following age groups receive ERM in your PICU? (Please select an option for each)

	Always	Very often	Sometimes	Seldom	Never	Don't know
Infants & <1 year old	☐	☐	☐	☐	☐	☐
1-4 years old	☐	☐	☐	☐	☐	☐
> 4-10 years old	☐	☐	☐	☐	☐	☐
> 10-18 years old	☐	☐	☐	☐	☐	☐

Comments:

9. How often do patients within the following diagnostic categories receive ERM in your PICU? (Please select an option for each)

	Always	Very often	Sometimes	Seldom	Never	Don't know
Acquired brain injury patients (e.g. TBI, meningitis)	☐	☐	☐	☐	☐	☐
Sepsis	☐	☐	☐	☐	☐	☐
Congenital heart disease	☐	☐	☐	☐	☐	☐
Cancer	☐	☐	☐	☐	☐	☐
Mechanically ventilated	☐	☐	☐	☐	☐	☐

Confidential: For Review Only

	Always	Very often	Sometimes	Seldom	Never	Don't know
Mechanically supported (e.g. ECMO)	☐	☐	☐	☐	☐	☐
Respiratory illness (bronchiolitis or pneumonia)	☐	☐	☐	☐	☐	☐
Multi-organ failure	☐	☐	☐	☐	☐	☐
With pre-existing physical co-morbidity	☐	☐	☐	☐	☐	☐
With pre-existing severe developmental delay	☐	☐	☐	☐	☐	☐

Comments:

10. How often do patients, with the following length of stay, receive ERM in your PICU? (Please select an option for each)

	Always	Very often	Sometimes	Seldom	Never	Don't know
Patients in PICU <3 days	☐	☐	☐	☐	☐	☐
Patients in PICU 3-7 days	☐	☐	☐	☐	☐	☐
Patients in PICU >7-28 days	☐	☐	☐	☐	☐	☐
Patients in PICU >28 days	☐	☐	☐	☐	☐	☐

Comments:

11. Who decides if the patient can start to receive ERM? (Please select all that apply)

☐ Medical consultant

☐ Other member of the medical team (e.g. PICU trainee/ advanced nurse practitioner)

Confidential: For Review Only

- [ ] Bedside nurse
- [ ] Senior nurse on shift
- [ ] Physiotherapist
- [ ] Occupational therapist
- [ ] Parent(s)
- [ ] Don't know
- [ ] Not applicable
- [ ] Other (please specify):

Comments:

12. How does your PICU assess whether patients tolerate and cope with the therapy provided? (eg monitor safety)

13. Do you currently use or have an established guideline or protocol regarding management of ERM in your PICU?

- [ ] No
- [ ] Yes - if you are able to share, please email to b.scholefield@bham.ac.uk
- [ ] Don't know

Comments:

4. Question regarding your established ERM programme or protocol

14. What do you have within your protocolised ERM treatment? (Please select all that apply)

Confidential: For Review Only

- [ ] Physical therapy not requiring additional equipment
- [ ] Physical therapy requiring additional equipment
- [ ] Occupational therapy
- [ ] Speech and language therapy
- [ ] Psychology
- [ ] Delirium screening
- [ ] We do not have a programme or protocol related to ERM activities
- [ ] Other (please specify):

Comments:

15. What are the key factors in successfully implementing your ERM protocol?

5. ERM related processes and equipment in your PICU

The following three questions are related to additional processes or equipment in your PICU related to ERM activities.

16. Does your PICU have a protocol or guidelines for the management of the following?

	Yes	No	Don't know
Sedation	☐	☐	☐
Analgesia	☐	☐	☐
Drug/ opioid withdrawal	☐	☐	☐
Delirium	☐	☐	☐

Confidential: For Review Only

	Yes	No	Don't know
Mobilisation (at any time point)	☐	☐	☐

17. What types of therapies or activities are available in your PICU? (Please select all that apply)

- ☐ Physical therapy - not requiring additional equipment (e.g. passive or active movements)
- ☐ Physical therapy - requiring additional equipment (e.g. in bed cycling)
- ☐ Occupational therapy
- ☐ Speech and language therapy
- ☐ Clinical psychology
- ☐ Delirium screening
- ☐ Patient diaries
- ☐ Play therapy
- ☐ Pet therapy
- ☐ Music therapy
- ☐ Other (please specify):

18. What equipment does your PICU have available for physical therapy/mobilisation? (Please select all that apply)

- ☐ Tilt table
- ☐ Bed with Trendelenburg features
- ☐ Bed with full chair position
- ☐ Bed with chair egress - exit out the foot of the bed
- ☐ Speciality bed with continuous side to side rotation
- ☐ Bed with retractable footboard i.e. flex a foot feature

Confidential: For Review Only

- ☐ Mobile lifts
- ☐ Ceiling lifts
- ☐ Portable ventilators
- ☐ Patient rolling walker
- ☐ Bedside cycle (patient can remain in bed)
- ☐ Transcutaneous electrical stimulation of muscles
- ☐ Specialist wheelchair
- ☐ Specialist static seating i.e. tumble forms
- ☐ Other (please specify):

6. Preferences, barriers and outcomes

For the following questions, we would explore your strength of opinion on ERM as a therapy, potential barriers to using ERM in practice and which are the most important clinical outcomes to consider when assessing if ERM is beneficial.

19. Please select one option regarding your current views of ERM in your PICU? *

- ☐ Crucial, should be the top priority in the care of PICU patients
- ☐ Very important, should be a priority in the care of PICU patients
- ☐ Important, should be a priority in the care of PICU patients
- ☐ Somewhat important, should be considered in the care of PICU patients
- ☐ Not of great importance, clinicians should bear it in mind in the care of PICU patients
- ☐ Of minimal importance to the care of PICU patients
- ☐ Of no importance to the care of PICU patients

Comments:

Confidential: For Review Only

20. Below are potential institutional barriers to the delivery of ERM. Please select to what extent you agree/disagree that each are barriers to delivery of ERM in your PICU. *

	Strongly Agree	Agree	Neutral	Disagree	Strongly Disagree
Instructions that patients should not move in their bed	☐	☐	☐	☐	☐
Consultant's permission or prescription is required prior to mobilisation	☐	☐	☐	☐	☐
Insufficient equipment/ resources	☐	☐	☐	☐	☐
Inadequate funding	☐	☐	☐	☐	☐
Lack of written guidelines/ protocol	☐	☐	☐	☐	☐
Lack of physical space	☐	☐	☐	☐	☐
Absence of champion/ advocate to promote ERM	☐	☐	☐	☐	☐
Absence of frequent (e.g. daily) ERM patient screening	☐	☐	☐	☐	☐

Comments:

21. Below are potential patient barriers to the delivery of ERM. Please select to what extent you agree/disagree that each are barriers to delivery of ERM in your PICU. *

	Strongly Agree	Agree	Neutral	Disagree	Strongly Disagree
Physiological instability ('the patient is just too sick')	☐	☐	☐	☐	☐

Confidential: For Review Only

	Strongly Agree	Agree	Neutral	Disagree	Strongly Disagree
Sedation level	☐	☐	☐	☐	☐
Presence of endotracheal intubation	☐	☐	☐	☐	☐
Risk of device/ line/ catheter dislodgement	☐	☐	☐	☐	☐
Cognitive impairment/ age	☐	☐	☐	☐	☐
Obesity	☐	☐	☐	☐	☐
Patient motivation	☐	☐	☐	☐	☐
Family concerns	☐	☐	☐	☐	☐

Comments:

22. Below are potential provider barriers to the delivery of ERM. Please select to what extent you agree/disagree that each are barriers to delivery of ERM in your PICU. *

	Strongly Agree	Agree	Neutral	Disagree	Strongly Disagree
Lack of prioritisation of ERM within patient care plans	☐	☐	☐	☐	☐
Limited staffing to deliver ERM	☐	☐	☐	☐	☐
Lack of coordination within and between clinician groups	☐	☐	☐	☐	☐
Slow recognition of patient readiness for ERM	☐	☐	☐	☐	☐
Conflicting perceptions concerning patient suitability	☐	☐	☐	☐	☐

Confidential: For Review Only

	Strongly Agree	Agree	Neutral	Disagree	Strongly Disagree
Safety concerns	☐	☐	☐	☐	☐
Lack of specific decision-making authority for ERM initiation	☐	☐	☐	☐	☐
Inadequate training	☐	☐	☐	☐	☐
Prolonging the current working day	☐	☐	☐	☐	☐

Comments:

23. Please rank what you perceive to be the top 5 most important benefits that ERM could provide in PICU. (Please select, in order, the top perceived benefits by selecting a box). *

	1 (most important)	2	3	4	5 (least important)
Reduction in treatment cost	☐	☐	☐	☐	☐
Reduction in patient delirium	☐	☐	☐	☐	☐
Reduction in length of ICU stay	☐	☐	☐	☐	☐
Reduction in days requiring mechanical ventilation	☐	☐	☐	☐	☐
Reduction in the rate of pulmonary complications	☐	☐	☐	☐	☐
Reduction in readmission (hospital and PICU)	☐	☐	☐	☐	☐
Increase in number of patients discharged home	☐	☐	☐	☐	☐
Improved patient satisfaction	☐	☐	☐	☐	☐

Confidential: For Review Only

	1 (most important)	2	3	4	5 (least important)
Improved staff satisfaction	☐	☐	☐	☐	☐
Improved family satisfaction	☐	☐	☐	☐	☐
Improvement in patient sleep quality	☐	☐	☐	☐	☐
Improvement in psychological impact of PICU care	☐	☐	☐	☐	☐
Improvement in daily life participation following discharge	☐	☐	☐	☐	☐

Comments:

7. Final thoughts

24. Would you be willing to support a future clinical trial in the UK and Ireland based on evaluating ERM practice?

☐ Yes

☐ No

☐ Don't Know

Comments:

25. Would you allow us to contact you in the future with information regarding workshops we will be running to discuss ERM practice and trial feasibility, please? *

☐ Yes (please click the link on the next page to provide best contact information, thank you!)

☐ No

Confidential: For Review Only

Funding statement: This study/project is funded by the National Institute for Health Research (NIHR) [Health Technology Assessment Programme (project reference: 17/21)/ HTA]. The views expressed are those of the author(s) and not necessarily those of the NIHR or the Department of Health and Social Care.

Confidential: For Review Only

Supplemental Tables

Supplemental Table 1: Characteristics of survey respondents (n=124 respondents)

**Professional group**	**n (%)***
Nurse	34 (27)
Physiotherapist	28 (23)
Medical doctor (consultant)	22 (18)
Occupational therapist	19 (15)
Play therapist	7 (6)
Psychologist	7 (6)
Dietician	6 (5)
Speech and Language therapist	1 (1)
**Years of experience**	**n (%)***
<1 year	7 (6)
1 to <5 years	27 (22)
5 to <10 years	30 (24)
10 to <15 years	14 (11)
15 to <20 years	33 (27)
More than 20 years	15 (12)

* Percentages may not total 100 due to rounding

Supplemental Table 2: Current views of ERM in PICU (n=121 respondents)

**Current view of ERM in PICU**	**n (%)***
Crucial, should be the top priority in the care of PICU patients	15 (12)
Very important, should be a priority in the care of PICU patients	67 (55)
Important, should be a priority in the care of PICU patients	35 (29)
Somewhat important, should be considered in the care of PICU patients	4 (3)
Not of great importance, clinicians should bear it in mind in the care of PICU patients	0 (0)
Of minimal importance to the care of PICU patients	0 (0)
Of no importance to the care of the PICU patients	0 (0)

* Percentages may not total 100 due to rounding

Confidential: For Review Only

Supplemental Table 3: Top 5 most important benefits of Early Rehabilitation and Mobilisation

Perceived ERM benefits (Most to least important)	Sum Rank Score*
Reduction in length of ICU stay	326
Improvement in the psychological impact of PICU care	288
Reduction in days requiring MV	242
Improvement in daily life participation following discharge	221
Improved patient satisfaction	158
Reduction in the rate of pulmonary complications	122
Reduction in patient delirium	95
Improved family satisfaction	94
Improvement in patient sleep quality	82
Reduction in treatment cost	60
Reduction in readmission	50
Increase in the number of patients discharged home	42
Improved staff satisfaction	27

*Sum of rank score: Ranking of top 5 (1-5) (1st placed rank scored 5 points to 5th placed scored 1 point). 121/124 (98%) participants ranked scores.

Supplemental Table 4: Which professional or parent groups in PICU intiates ERM (n=124 respondents)

Professional or family group	Yes n (%)
Physiotherapist	96 (77)
Physicians	92 (74)
Bedside nurse	64 (52)
Senior nurse	58 (47)
Other members of the medical team	55 (44)
Occupational therapist	37 (30)
Parent or family member	24 (19)

Confidential: For Review Only

Supplemental Table 5: Barriers of ERM delivery within Paediatric Intensive Care Units (n=124 respondents)*

**Institutional barriers**	**Strongly Agree n (%)**	**Agree n (%)**	**Neutral n (%)**	**Disagree n (%)**	**Strongly Disagree n (%)**	**Skipped responses[#] (n)**
Insufficient equipment/resources	31 (26)	52 (43)	22 (18)	13 (11)	3 (2)	3
Lack of written guidelines/protocol	31 (26)	38 (31)	35 (29)	11 (9)	6 (5)	3
Inadequate funding	30 (25)	43 (36)	28 (23)	16 (13)	4 (3)	3
Absence of champion/advocate to promote ERM	25 (21)	43 (36)	23 (19)	21 (17)	9 (7)	3
Absence of frequent ERM patient screening	25 (23)	38 (35)	24 (22)	14 (13)	8 (7)	14
Lack of physical space	17 (14)	51 (42)	17 (14)	32 (26)	4 (3)	3
Instructions that patients should not move in their bed	10 (8)	25 (21)	24 (20)	48 (40)	14 (12)	3
Consultant's permission or prescription is required prior to mobilisation	5 (4)	39 (32)	32 (26)	33 (27)	12 (10)	3
**Provider barriers**						
Slow recognition of patient readiness for ERM	19 (16)	46 (38)	19 (16)	35 (29)	2 (2)	3
Limited staffing to deliver ERM	42 (35)	54 (45)	11 (9)	13 (11)	1 (1)	3
Lack of prioritisation of ERM within patient care plans	27 (22)	45 (37)	22 (18)	24 (20)	3 (2)	3
Inadequate training	20 (17)	54 (45)	18 (15)	26 (21)	3 (2)	3
Conflicting perceptions concerning patient suitability	17 (14)	59 (49)	22 (18)	21 (17)	2 (2)	3
Lack of specific decision-making authority for ERM initiation	16 (13)	49 (40)	28 (23)	24 (20)	4 (3)	3
Safety concerns	15 (12)	55 (45)	32 (26)	18 (15)	1 (1)	3
Lack of coordination within and between clinician groups	14 (12)	44 (37)	30 (25)	27 (23)	5 (4)	4
Prolonging the current working day	4 (3)	21 (17)	43 (36)	41 (34)	12 (10)	3
**Patients barriers**						
Levels of physiological instability	48 (40)	53 (44)	12 (10)	7 (6)	1 (1)	3
Sedation level	27 (22)	64 (53)	15 (12)	13 (11)	2 (2)	3
Presence of endotracheal intubation levels	8 (7)	36 (30)	32 (26)	39 (32)	6 (5)	3
Risk of device/line/catheter dislodgement levels	8 (7)	42 (35)	32 (26)	35 (29)	4 (3)	3
Cognitive impairment/age levels	1 (1)	32 (26)	29 (24)	46 (38)	13 (11)	3
Obesity levels	6 (5)	14 (12)	35 (29)	52 (43)	14 (12)	3
Patient motivation level	2 (2)	38 (31)	31 (26)	43 (36)	7 (6)	3
Family concerns levels	2 (2)	42 (35)	35 (29)	38 (31)	4 (3)	3

Confidential: For Review Only

* Percentages may not total 100 due to rounding # Skipped responses excluded from percent calculation.

Confidential: For Review Only

Supplemental Table 6: Types of ERM equipment available in each Paediatric Intensive Care Unit (n=26 PICUs)

ERM equipment available in each PICU	n (%)
Specialist static seating	25 (96)
Portable ventilators	23 (88)
Mobile lifts	22 (85)
Tilt table	22 (85)
Bed with full chair position	18 (69)
Specialist wheelchair	18 (69)
Bed with Trendelenburg features	13 (50)
Patient rolling walker	11 (42)
Bedside cycle or inbed cycle	10 (38)
Ceiling lifts	8 (31)
Speciality bed with continuous side to side rotation	8 (31)
Bed with retractable footboard	7 (27)
Bed with chair egress exit out the foot of the bed	5 (19)
Transcutaneous Electrical Nerve stimulation	3 (12)

Confidential: For Review Only

Supplemental Material 3

Table 1: Descriptions of Early Rehabilitation and Mobilisation, ERM

Category	Sub-categories	Examples:
**Activity-focused** ERM is activity-focused, with consideration of seating and equipment, primarily delivered by nursing and allied health care professionals with optimised sedation management to promote patient engagement. There is a lack of consensus on what is routine care compared with purposeful ERM activity.	Activity – mobilising, positioning, stretching	"ERM provided includes - passive movements of limbs, periodic change of position, use of splints on extremities and if stable in the long term patients - sitting up in tumble form chair and mobilising out of bed." (Doctor, 67)<br><br>"We aim to get the children sitting upright, either over the edge of the bed or inappropriate seating as soon as possible." (Occupational Therapist, 001)<br><br>"Nursing staff are taught the importance of regular position changes for patient ... and positioning to maintain ranges of movement and prevent foot drop." (Nurse, 017)
	Core health care professional involvement – physiotherapist, nurse, occupational therapist	"We try and mobilise patients as soon as able when not invasively ventilated - physio led." (Doctor, 058)<br><br>"As physiotherapists, we work with the occupational therapist and nursing staff to either re-position, sit up in bed, sit on the edge of the bed or stand whilst on the ventilators."(Physiotherapist, 069)
	Additional health care professional involvement – psychology, dietician, play therapist, speech and language therapists	"Speech and language therapy become involved usually when nursing or medical staff identify a need for referral." (Doctor, 003)<br><br>"We have a Play Specialist on PICU, who assists with communication tools/toys." (Nurse, 082)
	Seating and equipment	"Specialist seating need – linking in with OT to ensure appropriate seating available." (Physiotherapist, 019)

Confidential: For Review Only

		"We have also in the past asked adult [services] to use their moto-med bike and used this with teenagers, but this can be a challenge as it is very far from PICU and it is often in use." (Physiotherapist, 014)
	Sedation management	"Working with the medics to wean sedation as quickly as possible. This promotes faster ability to mobilise and progress in their rehabilitation." (Nurse, 034)
	'Routine care' vs 'purposeful ERM package'	Some centres define ERM as passive range of movement, repositioning or providing splints. We would deem this as essential core cares rather than ERM; the large majority of our patients will have positioning and movement plan from day 1 of admission." (Physiotherapist, 111)
**Tailored**<br>Following the assessment of needs and patient and family preference, ERM activities are personalised to the individual patient.	Assessment of need	"We have a programme where patients are categorised to one of 3 levels. Each level provides nursing and therapy staff with activities aligned to the acuity of the patient." (Nurse, 040)
	Individual preference	"Parents will tell us what their child enjoys doing and make suggestions, often provide toys/games from home." (Nurse, 076)<br><br>"Discuss with parents what patient enjoys doing, watching etc. Discuss options regarding taking patients 'out' where possible if long term patients." (Nurse, 015)
**Promote recovery**<br>The purpose of ERM is to normalise the PICU environment, to create an environment that addresses holistic needs, sustains or promotes development and supports recovery from critical illness.	Normalising PICU environment	"Provide activities they would use for their enjoyment. This enables them to be more relaxed and less aware of what is going on with / around them." (Play specialist, 102)<br><br>"Being in PICU can be a frightening experience. Not only is a child/young person away from home, but also away from their usual environment, family and friends. There are unfamiliar sounds, smells, equipment and people. The play specialist can help to make the stay much more enjoyable and children/young people to cope and understand." (Play specialist, 055)
	Sustain / promote child development	"Encouraging the patient to regain or further their development in a therapeutic fun manner." (Play therapist,102)

Confidential: For Review Only

	Restoration / recovery	"Interventions aim to promote physical recovery – movement, and ability to engage in activities and psychological recovery – orientation, speech, ability to play and attend school. " (Nurse, 021)
**Timing of ERM**<br>The optimal timing of ERM initiation is challenging for health care professionals to define, although likelihood perceived to increase with increased length of stay. Influenced by the perceived stability of patients and the balance of risk to benefit.	'Early' poorly defined	"I like to think we consider it within 2-3 days we have a better idea of the patients PICU journey and how long they are going to stay. The reality is it's usually longer... usually week 2 of admission if they are still on the unit." (Physiotherapist, 090)<br><br>"There is no formal plan for who decides if a patient should start ERM and when… Currently the delivery of ERM is fairly ad hoc, based on what is highlighted from daily handover each morning." (Physiotherapist, 083)<br><br>"We provide ERM as a structured programme… every patient after 24 hours of admission is considered for ERM." (Doctor, 012)
	Patient stability, risk and benefit	"Members of the PICU team with less experience of early rehab can deem ERM 'unsafe' which can occasionally act as a barrier."(Physiotherapist, 016)<br><br>"Usually ERM activity is not considered until patients can physiologically tolerate movement and are stable with observations." (Nurse, 008)
	'Long-stay'	"ERM is often thought about as a patient comes up to extubation or has failed extubation, and we feel that the reason for failure may be because of critical care weakness." (Physiotherapist, 031)<br><br>"We provide very little ERM on PICU, other than for long term patients, which is often, in my opinion, delayed." (Nurse, 007)

Confidential: For Review Only

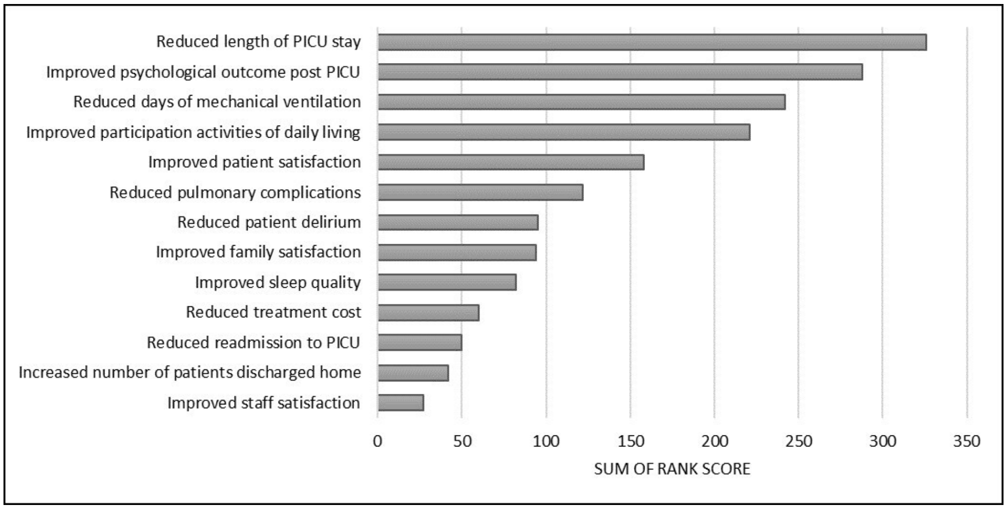

88x44mm (300 x 300 DPI)

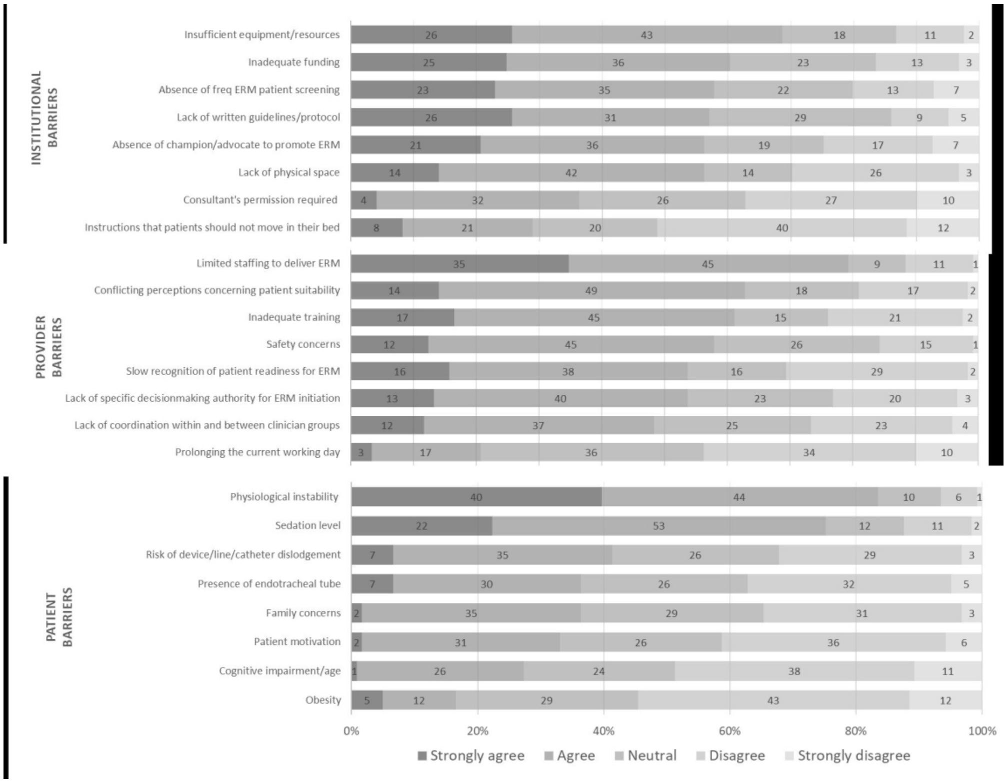

153x119mm (300 x 300 DPI)

Figure 1: Perceived Benefits of Early Rehabilitation and Mobilisation

Ranking of participants potential top 5 perceived benefits of delivering ERM within PICU's. Sum of rank score: Ranking of top 5 (1-5) (1st placed rank scored 5 points to 5th placed scored 1 point). 121/124 (98%) participants ranked scores.

Confidential: For Review Only

Figure 2: Perceived Barriers of Early Rehabilitation and Mobilisation
Institutional, Patients and Provider barriers to ERM. Figure 2 shows the percentage of responses for categories strongly agree, agree, neutral, disagree and strongly disagree. Responses ranked on the cumulative score of percentage 'strongly agree and agree'.

Confidential: For Review Only

BMJ Paediatrics Open

Early Mobilisation and Rehabilitation in the PICU: A UK survey

Journal:	*BMJ Paediatrics Open*
Manuscript ID	bmjpo-2021-001300.R2
Article Type:	Original research
Date Submitted by the Author:	02-Feb-2022

Complete List of Authors:	Thompson, Jacqueline; University of Birmingham, ; University of Birmingham<br>Menzies, Julie; Birmingham Women's and Children's NHS Foundation Trust, Paediatric Intensive Care<br>Manning, Joseph ; Nottingham University Hospitals NHS Trust, Nottingham Children's Hospital; University of Nottingham, Children and Young people Health Research, School of Health Sciences<br>McAnuff, Jennifer; Newcastle University, Population Health Sciences Institute, Newcastle University; Northumbria University, Department of Social Work, Education and Community Wellbeing<br>Brush, Emily; University of Birmingham<br>Ryde, Francesca; University of Birmingham<br>Rapley, Tim; Northumbria University<br>Pathan, Nazima; University of Cambridge, Paediatrics<br>Brett, Stephen; Imperial College of Science Technology and Medicine, Department of surgery and cancer<br>Moore, David; University of Birmingham<br>Geary, Michelle; University Hospital Southampton NHS Foundation Trust, Child Health<br>Colville, Gillian; St George's University Hospitals NHS Foundation Trust, Paediatric Psychology Service<br>Morris, Kevin; Birmingham Women's & Children's NHS Foundation Trust<br>PARSLOW, ROGER; UNIVERSITY OF LEEDS, PAEDIATRIC EPIDEMIOLOGY GROUP<br>Feltbower, Richard; UNIVERSITY OF LEEDS<br>Lockley, Sophie; Birmingham Children's Hospital<br>Kirkham, Fenella; Neurosciences Unit, UCL Institute of Child Health<br>Forsyth, Rob; Newcastle University, Institute of Neuroscience<br>Scholefield, Barnaby; University of Birmingham

Keywords:	Rehabilitation, Epidemiology

SCHOLARONE™
Manuscripts

I, the Submitting Author has the right to grant and does grant on behalf of all authors of the Work (as defined in the below author licence), an exclusive licence and/or a non-exclusive licence for contributions from authors who are: i) UK Crown employees; ii) where BMJ has agreed a CC-BY licence shall apply, and/or iii) in accordance with the terms applicable for US Federal Government officers or employees acting as part of their official duties; on a worldwide, perpetual, irrevocable, royalty-free basis to BMJ Publishing Group Ltd ("BMJ") its licensees and where the relevant Journal is co-owned by BMJ to the co-owners of the Journal, to publish the Work in this journal and any other BMJ products and to exploit all rights, as set out in our licence.

The Submitting Author accepts and understands that any supply made under these terms is made by BMJ to the Submitting Author unless you are acting as an employee on behalf of your employer or a postgraduate student of an affiliated institution which is paying any applicable article publishing charge ("APC") for Open Access articles. Where the Submitting Author wishes to make the Work available on an Open Access basis (and intends to pay the relevant APC), the terms of reuse of such Open Access shall be governed by a Creative Commons licence – details of these licences and which Creative Commons licence will apply to this Work are set out in our licence referred to above.

Other than as permitted in any relevant BMJ Author's Self Archiving Policies, I confirm this Work has not been accepted for publication elsewhere, is not being considered for publication elsewhere and does not duplicate material already published. I confirm all authors consent to publication of this Work and authorise the granting of this licence.

Confidential: For Review Only

Early Mobilisation and Rehabilitation in the PICU: A UK survey

Manuscript type: Original Research

Authors:

Thompson JY[1], Menzies JC[2], Manning JC[3,4], McAnuff J[5, 6], Brush E[1], Ryde F[1], Rapley TR[6], Pathan N[7], Brett S[8], Moore D[9], Geary M[10], Colville G[11], Morris KP[2], Parslow RC[12], Feltbower RG[12], Lockley S[13], Kirkham F[10], Forsyth R[14], Scholefield B R[1,2], On behalf of the PERMIT Collaborators & Paediatric Intensive Care Society Study-Group (PICS-SG)

Author affiliations:

1. Birmingham Acute Care Research Group, Institute of Inflammation and Ageing, University of Birmingham
2. Paediatric Intensive Care, Birmingham Women's & Children's NHS Foundation Trust
3. Nottingham Children's Hospital, Nottingham University Hospitals NHS Trust
4. Children and Young People Health Research, School of Health Sciences, The University of Nottingham
5. Population Health Sciences Institute, Newcastle University
6. Department of Social Work, Education and Community Wellbeing, Northumbria University
7. Department of Paediatrics, The Chancellor, Masters, and Scholars of the University of Cambridge
8. Department of surgery and cancer; Imperial College of Science, Technology and Medicine

Confidential: For Review Only

9. Institute of Applied Health, University of Birmingham

10. Child Health, University Hospital Southampton NHS Foundation Trust

11. Paediatric Psychology Service; St George's University Hospitals, NHS Foundation Trust

12. Leeds Institute for Data Analytics, School of Medicine, University of Leeds, Leeds

13. PPI Representative

14. Translational and Clinical Research Institute, Newcastle University

Corresponding author:

Barnaby R Scholefield; B.Scholefield@bham.ac.uk

Twitter handle: @BarneyUoB

Abstract word count: 264 words now

Main text word count (excluding abstract, reference list, tables, and figures): 2732

References: 27

Tables: 6

Figures: 2

Supplementary files: 2 (1 survey, 3 supplemental tables)

Confidential: For Review Only

Abstract

Objective: To understand the context and professional perspectives of delivering early rehabilitation and mobilisation (ERM) within UK Paediatric Intensive Care Units (PICU).

Design: A web-based survey administered May-August 2019.

Setting: United Kingdom PICUs.

Participants: A total of 124 staff from 26 PICUs participated, including 22 (18%) Doctors, 34 (27%) Nurses, 28 (23%) Physiotherapists, 19 (15%) Occupational therapists, and 21 (17%) were other professionals.

Results: Key components of participants' definitions of ERM included tailored, multi-disciplinary rehabilitation packages focused on promoting recovery. Multi-disciplinary involvement in initiating ERM was commonly reported. Over half of respondents favoured delivering ERM after achieving physiological stability (n=69, 56%). All age groups were considered for ERM by relevant health professionals. However, responses differed concerning the timing of initiation. Interventions considered for ERM were more likely to be delivered to patients when PICU length of stay exceeded 28 days and among patients with acquired brain injury or severe developmental delay. The most commonly identified barriers were: physiological instability (81%), limited staffing (79%), sedation requirement (73%), insufficient resources and equipment (69%), lack of recognition of patient readiness (67%), patient suitability (63%), inadequate training (61%), and inadequate funding (60%). Respondents ranked reduction in PICU length of stay 74% and improvement in psychological outcomes 73% as the most important benefits of ERM.

Confidential: For Review Only

Conclusion: ERM is gaining familiarity and endorsement in UK PICUs, but significant barriers to implementation due to limited resources and variation in content and delivery of ERM persist. A standardised protocol that sets out defined ERM interventions, along with implementation support to tackle modifiable barriers, is required to ensure the delivery of high-quality ERM.

Confidential: For Review Only

INTRODUCTION

In the UK, approximately 20,000 children are admitted to Paediatric Intensive Care Units (PICU) yearly[1], and although most recover, some develop longer-term physical, psychological and cognitive impairment[2]. These significant morbidities have been termed Post Intensive Care Syndrome in Paediatrics (PICS-p)[3]. Early rehabilitation and mobilisation (ERM) encompass patient-tailored interventions, delivered within seven days of admission, individually[4] or in a bundled package[5] to patients within intensive care settings. It is provided by health professionals from multiple disciplines and care-givers and may promote physical (e.g. functional activities)[4] and non-physical (e.g. psychological, cognitive)[6] recovery.

Within adult ICUs, ERM has been demonstrated to be safe, feasible and cost-effective[7 8]. It can shorten the length of ventilation, duration of intensive care and hospital stay with economic benefit, improve long term physical functioning and return to independence, and is recommended by National Institute for Health Care Excellence (NICE)[8]. Whilst ERM has been reported to be safe and feasible in PICUs [9-11], the patient population is different, and ERM is less well defined, leading to variability in practice. This variation in practice and components of interventions delivered has also been described in European PICU's[12]. At present, no national standardised care pathway for ERM exists within UK PICUs.

This study forms part of the National Institute for Health Research (NIHR) funded PERMIT study (NIHR HTA: 17/21/06) investigating ERM in children. This work set out to understand reported practices and perceptions of ERM within the UK PICU context.

Confidential: For Review Only

Our objectives were to:

I. Explore how health care professionals describe and administer ERM using a qualitative approach
II. Identify and describe current ERM practice using a quantitative approach

Understand and quantify perceived barriers and facilitators of ERM, presenting findings using descriptive analysis.METHODS

A web-based survey (administered through www.smartsurvey.co.uk) was developed that included 25 questions (Supplementary material file 1). A patient representative was involved in the design and development of the survey. A pilot survey was conducted among multi-disciplinary health professionals (n=40) teams within two PICUs to assess acceptability and comprehensiveness. There were very few missing responses; therefore, no questions were removed, but five questions were rephrased to add clarity. Pilot responses were excluded from the main survey analysis.

A UK Paediatric Intensive Care Society Study Group (PICS-SG) member of each UK PICU (n=29) was contacted via email and requested to identify and cascade to members of their local multi-disciplinary team (including at least one physiotherapist, doctor and nurse) to complete the survey. Participating PICUs were sent a survey link to distribute between May and August 2019. Three follow up reminders were sent at weekly intervals to non-responders.

Likert scales with the following categories (and associated scores) – always (5), very often (4), sometimes (3), seldom (2) and never (1) - were used to express the frequency of

Confidential: For Review Only

practice or level of agreement (using median and IQR of score). The ranking of perceived ERM benefits was calculated using the sum of ranked scores of respondents top 5 important benefits (5 points for 1st, reducing to 1 point for 5th placed). Statistical analysis was performed using R version x64 3.5.1 (R Foundation for Statistical Computing, Vienna, Austria).

Open-ended responses were analysed using a qualitative content analysis approach[13]. Two researchers independently familiarised themselves with the data and conducted open-coding, utilising NVIVO™ software for data management. Codes were then discussed, summarised and organised[14 15]. In relevant sections of the paper, free text quotes from respondents are reported to add context and clarity[16]. The University of Birmingham granted institutional ethical approval on 05/02/2019 (reference ERN_18-1134). Consent was implied through survey completion.

Patient and Public Involvement

The pilot survey received input from multi-disciplinary health professionals who were members of the UK Paediatric Intensive Care Society Study Group (PICS-SG). We also recruited a patient representative as part of the study team to ensure the perspective, experiences and preferences of children admitted to PICU were incorporated into the design and development of the survey. The patient representative was a parent of a child who had been admitted to PICU. They both contributed to the nature of questions considered in the survey to ensure the study outcome would be relevant to parents and members of the PICS-SG on dissemination.

https://mc.manuscriptcentral.com/bmjpo

Confidential: For Review Only

RESULTS

Demographics

We received responses from PICS-SG link members in 26/29 (90%) UK PICUs. A total of 191 healthcare professionals opened the survey link, with 124 (65%) submitting responses.

Table 1: Characteristics of survey respondents (n=124 respondents)

**Professional group**	**n (%)***
Nurse	34 (27)
Physiotherapist	28 (23)
Medical doctor (consultant)	22 (18)
Occupational therapist	19 (15)
Play therapist	7 (6)
Psychologist	7 (6)
Dietician	6 (5)
Speech and Language therapist	1 (1)
**Years of experience**	**n (%)***
<1 year	7 (6)
1 to <5 years	27 (22)
5 to <10 years	30 (24)
10 to <15 years	14 (11)
15 to <20 years	33 (27)
More than 20 years	15 (12)

* Percentages may not total 100 due to rounding

As shown in Table 1, the majority of respondents were nurses (n=34, 27%), physiotherapists (n=28, 23%) and doctors (n=22, 18%). There were also responses from occupational therapists (n=19, 15%), play therapists (n=7, 6%), psychologists (n=7, 6%), dieticians (n=6, 5%), and speech and language therapists (n=1, 1%). Almost three-quarters of health professionals had ≥5 years experience, with 48 (39%) ≥15 years. The majority of respondents considered ERM to be a priority, either crucial 15 (12%), very important 67 (55%) or important 35 (29%) in the care of PICU patients (Table 2).

Confidential: For Review Only

Table 2: Current views of ERM in PICU (n=121 respondents)

Current view of ERM in PICU	n (%)*
Crucial, should be the top priority in the care of PICU patients	15 (12)
Very important, should be a priority in the care of PICU patients	67 (55)
Important, should be a priority in the care of PICU patients	35 (29)
Somewhat important, should be considered in the care of PICU patients	4 (3)
Not of great importance, clinicians should bear it in mind in the care of PICU patients	0 (0)
Of minimal importance to the care of PICU patients	0 (0)
Of no importance to the care of the PICU patients	0 (0)

* Percentages may not total 100 due to rounding

Confidential: For Review Only

Description of ERM

We invited respondents to describe ERM on their terms. Descriptions were provided by 104 (84%), which were summarised into four categories, 'Activity-focused', 'Tailored', 'Promote recovery', and 'Timing of ERM' (see Supplementary file 2, Table 1). Overall, ERM was considered to be an individualised package of graded interventions based on an activity-focused programme, to reduce the sequelae of critical illness or injury. However, responses differed for when ERM should be initiated, often emphasising the need for individualisation.

Availability of established ERM Protocols

Respondents were asked to describe the content of established ERM protocols within their PICU. Only 12 participants (10%) reported working in a PICU with an established ERM protocol (n=5/26, 19% of PICUs). The most common components of ERM protocols were 'physical therapy not requiring additional equipment' (n=9/12, 75%) and 'occupational therapy interventions' (n=8/12, 67%). Only 4/12 (33%) referred to play therapy or speech and language therapy, and no ERM protocol specified input from psychologists or psychiatrists. All participants were asked about the content of non-ERM protocols in their

PICU. Only 18/124 (15%) reported that guidance for physical or occupational therapy activities existed in other non-ERM protocols within PICU (Table 3).

Table 3: Content of ERM and non-ERM protocols

Items	Within an ERM protocol (n=12 respondents) Yes n (%)	Within a non-ERM protocols (n=124 respondents) Yes n (%)
Physical therapy requiring additional equipment	9 (75)	18 (15)
Occupational therapy interventions	9 (75)	18 (15)
Physical therapy not requiring additional equipment	8 (67)	17 (14)
Speech and language therapy interventions	4 (33)	12 (10)
Psychology interventions	0 (0)	8 (6)
Delirium screening	0 (0)	1 (1)

Recipients of ERM

51 (41%) respondents reported that *All PICU patients* 'always' or 'very often' received ERM (Supplementary file 2, Table 2). Overall, 14 (11%) respondents reported 'seldom' or 'never' delivering ERM.

ERM was reported to be more likely to be delivered to patients when PICU length of stay exceeded 28 days. Patients admitted for 28 days or more were more likely (n=91, 75%) to 'always' or 'very often' receive ERM compared to those admitted for shorter periods. Only 17 (13%) of those staying fewer than three days, 44 (36%) of those admitted between three to seven days and 73 (59%) of those admitted between seven to 28 days were more likely to receive ERM. . Participants reported that patients with acquired brain injury (n=75, 60%) and severe developmental delay (n=54, 44%) were 'always' or 'very often' likely to receive ERM.

Confidential: For Review Only

Perceived benefits of ERM

Participants ranked the five most important potential benefits of ERM out of thirteen options (Figure 1 and Table 4). The most important outcomes identified were: 1) reduced PICU length of stay, 2) improved psychological outcomes for patients after PICU, 3) reduced days of mechanical ventilation, 4) improved participation in activities of daily living and 5) improved patient satisfaction.

Table 4: Top 5 most important benefits of Early Rehabilitation and Mobilisation

Perceived ERM benefits (Most to least important)	Sum Rank Score*
Reduction in length of ICU stay	326
Improvement in the psychological impact of PICU care	288
Reduction in days requiring MV	242
Improvement in daily life participation following discharge	221
Improved patient satisfaction	158
Reduction in the rate of pulmonary complications	122
Reduction in patient delirium	95
Improved family satisfaction	94
Improvement in patient sleep quality	82
Reduction in treatment cost	60
Reduction in readmission	50
Increase in the number of patients discharged home	42
Improved staff satisfaction	27

*Sum of rank score: Ranking of top 5 (1-5) (1st placed rank scored 5 points to 5th placed scored 1 point). 121/124 (98%) participants ranked scores.

Initiation and delivery of ERM

The decision for ERM initiation was perceived by 96 (77%) to be primarily led by physiotherapists, doctors (n=92, 74%), and bedside nurses (n=64, 52%). Parents were felt to initiate ERM by only (n=24, 19%) of respondents (Table 5).

Confidential: For Review Only

Table 5: Which professional or parent groups in PICU initiates ERM (n=124 respondents)

Professional or family group	Yes n (%)
Physiotherapist	96 (77)
Physicians	92 (74)
Bedside nurse	64 (52)
Senior nurse	58 (47)
Other members of the medical team	55 (44)
Occupational therapist	37 (30)
Parent or family member	24 (19)

The most influential factor in ERM initiation was reported to be patient stability (n=69, 56%). Other influential factors were the length of stay; 15 (12%) reported ERM was initiated within 24 hours, and 16 (13%) within 2-3 days of PICU admission. Only 5 (4%) of respondents would not consider ERM at all on PICU. The influence of perceived clinical stability is demonstrated in respondents' free-text comments:

> "We are involved as early as required depending on the child/young person medical stability and their rehabilitation needs." (Occupational Therapist, 033)

> "Usually ERM activity is not considered until patients can physiologically tolerate movement and are cardiovascularly stable." (Nurse, 008)

Assessment of patient stability and tolerance of ERM was less well described. Most respondents (n=98, 79%) provided subjective cues or informal clinical criteria. These included monitoring vital signs, physiological changes, observing behavioural changes, and documenting adverse events.

Confidential: For Review Only

Physiotherapists (n=113, 92%), nurses (n=103, 84%), and parents or family members (n=92, 75%) were 'always' or 'very often' involved in the ongoing delivery of ERM with less frequent input from other members of the multi-disciplinary teams (Supplemental material file 2, Table 2).

Barriers to ERM Implementation

Figure 2 presents the perceived barriers of ERM (see also Supplemental material file 2, Table 3). The most significant barriers identified as institutional barriers were insufficient resources and equipment ('strongly agree' or 'agree': 83, 69%) and inadequate funding (73, 61%). Participants provided examples of resources having to be shared across organisations or specially ordered to deliver ERM to patients.

> "All equipment shared with the whole therapy department at present, therefore dependent on availability." (OT, 010)

A lack of established protocols (n=69, 57%), ERM champions (n=68, 57%), space (n=68, 56%), and robust patient screening processes (n=63, 58%) were also issues identified by respondents. Most PICUs had access to standard lifting 22/26 (85%) and specialist static seating equipment 25/26 (96%); however, bedside or in-bed cycling machines were only available in 10 (38%) of PICUs (see Table 6).

Limited staffing was the most frequently reported barrier to providers delivering ERM, with 101 (79%) 'agreeing' or 'strongly agreeing'. Approximately half of the respondents agreed that issues such as training, patient safety, lack of decision-making authority, and delays in recognising patients' ERM needs were barriers to ERM initiation. However, only 25 (21%)

Confidential: For Review Only

'agreed' or 'strongly agreed' that the impact of ERM potentially prolonging the working day was a barrier.

Table 6: Types of ERM equipment available in each Paediatric Intensive Care Unit (n=26)

ERM equipment available in each PICU	n (%)
Specialist static seating	25 (96)
Portable ventilators	23 (88)
Mobile lifts	22 (85)
Tilt table	22 (85)
Bed with full chair position	18 (69)
Specialist wheelchair	18 (69)
Bed with Trendelenburg features	13 (50)
Patient rolling walker	11 (42)
Bedside cycle or inbed cycle	10 (38)
Ceiling lifts	8 (31)
Speciality bed with continuous side to side rotation	8 (31)
Bed with retractable footboard	7 (27)
Bed with chair egress exit out the foot of the bed	5 (19)
Transcutaneous Electrical Nerve stimulation	3 (12)

The two most frequently reported barriers to delivering ERM at the patient level were physiological instability (n=101, 81% agreeing or strongly agreeing) and sedation (n=91, 73%). Over 50% (n=64) 'disagreed' or 'strongly disagreed' that obesity was a barrier.

DISCUSSION

This national survey of health care practitioners from UK PICU's identified the importance of ERM as an intervention that participants believe can improve the physical and psychological recovery of critically ill or injured infants and children across all ages. Our findings indicate

Confidential: For Review Only

support for ERM but highlight uncertainty with suitability, variability with the definition of this complex intervention, variation in the timing of initiation and which patient groups should receive ERM. Key barriers to ERM delivery were identified (e.g. funding and staffing) and potential clinical (e.g. improved psychological outcomes) and economic (e.g. reduced PICU length of stay) benefits to patients and PICUs.

How early is 'early' in ERM has been challenging to define for health care professionals? A time-based definition (e.g. within the first 2-5 days of ICU admission) has been proposed[17]; however, this can conflict with the patient stability approach (e.g. start as early as the patient is able to receive it). Our results indicate uncertainty and wide variation in time to start ERM (24hrs to over seven days), increasing agreement for ERM to be considered after longer periods on PICU, and support for the concept of 'as early as the patient's clinical condition allows' which may be much longer. In the UK, only 43% of patients remain in PICU for three days or more[1]. The balance of delivering a programme of ERM to a large number of patients who may only receive intervention within a very short period versus targeting patients at higher risk of prolonged PICU stay needs to be considered. However, it has been proposed that for ERM to become embedded in clinical practice, it should become a standard of care within 48hrs of ICU admission[18].

The uncertainty of the content of ERM also adds to the challenge for health care professionals to appreciate when ERM could be delivered. Understandably, routine bedside nursing care (e.g. functional positioning) may be considered acceptable earlier than more advanced physical therapies requiring multiple staff (e.g. sitting a ventilated child out of bed or in-bed cycling). Our survey identified clinical stability as the most influential patient factor for initiation. The reported lack of ERM protocols in most (21/26) UK PICUs reinforces a

Confidential: For Review Only

strong requirement for evidence-based standardised protocols with optimal timing, intensity, frequency and duration of ERM. There is a need for flexible protocols to allow for tailoring rather than prescription.

ERM was more likely to be delivered to patients admitted for greater than 28 days among patients with acquired brain injury or severe developmental delay across all age ranges. This reflects the cross-over between ERM and established rehabilitation programmes following acquired brain injury[19] or for patients admitted to PICU with a pre-existing rehabilitation package. PICUs may be able to build on the success of established programmes to implement ERM to a wider critical care population and to use the existing multi-disciplinary expertise, and evolving rehabilitation evidence base to support the adoption of effective treatments. To date, most published ERM intervention studies have excluded patients less than three years of age[4 6]. However, this represents 60% of the UK PICU patient population[1], and this age group was as likely to receive ERM as older children in our study. Future ERM trials should include all PICU age groups to ensure ERM content and efficacy is assessed across all potential patients.

Our results show that doctors, physiotherapists, and nurses have an equally important role in the decision to initiate ERM within the UK NHS setting. This contrasts with other countries and health care settings where doctor-led approval was required by 70-81% of respondents, which was a potential barrier to mobilisation[20 21]. Nurses' and parents' roles are also important both in the initiation and delivery of ERM. In settings where nurses reported a low level (39%) of support for ERM, lack of involvement and understanding were key features[22]. In our study, 91% felt 'involved' in the delivery of ERM. However, health care professionals reported that parents were the least likely group to initiate ERM (19%),

https://mc.manuscriptcentral.com/bmjpo

Confidential: For Review Only

although becoming influential in its ongoing delivery. This is consistent with recent European[12] and North-American[11 23 24] point prevalence studies in PICUs, highlighting the important role parents played in delivering ERM. Building on both the multi-disciplinary support for ERM and empowering parents to initiate ERM may be potential strategies to improve implementation.

The key barriers to ERM practice were: i) at institutional level: insufficient resources, equipment and funding; ii) at provider level: limited staffing, training, protocols and slow recognition of readiness for ERM; and iii) at patient-level: physiological instability**,** risk of endotracheal tube dislodgement and amount of sedation. These barriers have been previously described in other health settings[20-22 25] with proposed facilitators, including adopting formal protocol/manual[26] (a key feature lacking across most UK PICUs), local champions[26] and team engagement / collaboration[26 27]. Implementation of ERM within the NHS will require building on the established multi-disciplinary teams, incorporating ERM within established protocols for weaning off mechanical ventilation and sedation and realistic goal setting. The potential benefits of ERM in terms of PICU length of stay and improvement in the psychological outcome for patients should be assessed within the core outcomes evaluating the efficacy of ERM within PICU.

The strength of this survey was an inclusive representation of 90% of UK PICUs and views from the wider multi-disciplinary team. However, none or partial responses may indicate poor engagement in ERM topic, and as with all self-reported surveys, responses indicate reported rather than actual clinical practice. A limitation was the use of a non-validated questionnaire. We did not conduct questionnaire validation because it would be very difficult to standardise these questions within the time constraints of the study and the

Confidential: For Review Only

NIHR HTA funding. The study team considered the questionnaire suitable for the study aims after receiving two rounds of expert reviews. Finally, the findings represent the views of UK NHS staff and may not be generalisable to other healthcare settings.

CONCLUSION

ERM, in some form, is currently delivered to critically ill or injured patients of all ages across UK PICUs, but significant barriers to full implementation exist due to resource limitations and lack of institutional and national guidance. The UK should build on the existing strong multi-disciplinary support for ERM in PICUs. A standardised protocol that sets out defined ERM interventions and implementation support to tackle modifiable barriers is required to ensure the delivery of high-quality ERM.

Confidential: For Review Only

Acknowledgements This study/project is funded by the National Institute for Health Research (NIHR), project reference: 17/21/ HTA (Health Technology Assessment). The views expressed are those of the author(s) and not necessarily those of the NIHR or the Department of Health and Social Care.

We thank the Paediatric Intensive Care Society Study Group (PICS-SG), all members of the Paediatric Intensive Care Units and Principal Investigators for their contribution to the development of the PERMIT survey.

- Addenbrooke's Cambridge, Dr Nazima Pathan
- Alder Hey Children's NHS Foundation Trust, Liverpool, Dr Petr Jirasek
- Birmingham Women & Children's Hospital NHS Foundation Trust, Dr Barnaby Scholefield
- Bristol Royal Hospital for Children, Kate Baptiste
- Evelina London Children's Hospital, Dr Miriam Fine-Goulden
- Freeman Hospital, Newcastle upon Tyne Hospitals NHS Foundation Trust, Dr Deborah Cross
- GOSH CICU, Great Ormond Street Hospital for Children NHS Foundation Trust, Emma Shkurka
- GOSH PICU, Great Ormond Street Hospital for Children NHS Foundation Trust, Dr Peter Sidgwick
- Great North Children's Hospital, Newcastle, Dr Rachel Agbeko
- Kings College Hospital NHS Foundation Trust, Dr Bogdana Zoica
- Leeds Teaching Hospitals NHS Trust, St. James's University Hospital, Dr Sian Cooper
- Noah's Ark Children's Hospital for Wales, Dr Siva Oruganti
- Nottingham Children's Hospital, Queen's Medical Centre, Dr Georgina Harlow
- Oxford University Hospitals, Dr Deirdre O'Shea
- Royal Belfast Hospital for sick children, located in Royal Victoria Hospital, Dr Julie Richardson
- Royal Hospital for Children Glasgow, Dr Richard Levin
- Royal Hospital for Sick Children in Edinburgh, Dr Tsz Yan and Dr Milly Lo
- Royal Manchester Children's Hospital, Dr Ravishankar Nagaraj
- Sheffield Children's Hospital NHS Foundation Trust, Dr Rum Thomas
- Southampton Children's Hospital, Katy Morton
- St Georges Hospital, London, Dr Soumendu Manna
- St Mary's Hospital, Praed Street, Imperial London, Dr Tom Bycroft
- University Hospital Leicester, Dr Sanjiv Nichani
- Glenfield Hospital, Dr Sanjiv Nichani
- University Hospital North Midlands, Dr Kanaris Constantinos
- South Tees Hospitals NHS Foundation Trust

Contributions

BRS, JCMen, JCMan, RF, TR, NP, SB, JM, DM, MG, GC, KM, RP, RF, SL, and FK developed the original study protocol. BRS, EB, JCMan and FR developed the pilot survey and preliminary analysis. JT, JCMen, JCMan and BRS developed the final survey, coordinated administration, interpretation and analysis of data and BRS and JT drafted the manuscript, which has had input from all authors. All authors revised and approved the final manuscript. Paediatric

Confidential: For Review Only

Intensive Care Society Study-Group (PICS-SG) members reviewed the protocol and endorsed the survey.

Competing interests: Dr Julie C. Menzies is funded as a National Institute for Health Research (NIHR) 70@70 Senior Nurse and Midwife Research Leader. Dr Joseph C. Manning is an NIHR Health Education England funded ICA Clinical Lecturer. Dr Barnaby Scholefield receives funding as an NIHR Clinician Scientist Fellow, and Dr Forsyth and Prof Rapley receives NIHR grant funding. The views expressed in this article are those of the authors and not necessarily those of the NIHR, or the Department of Health and Social Care.

Funding informtion: This study/project is funded by the National Institute for Health Research (NIHR) [Health Technology Assessment Programme (project reference: 17/21)/ HTA]. The views expressed are those of the author(s) and not necessarily those of the NIHR or the Department of Health and Social Care.

Patient consent for publication: Not required.

Ethics approval: The study received ethical approval granted by the University of Birmingham review panel on 05/02/2019

Confidential: For Review Only

What is already known?

- Early rehabilitation and mobilisation (ERM) interventions are safe, feasible and effective within adult intensive care, but the evidence base in paediatric setting is limited.
- In critically ill adults, ERM delivery is tailored according to patient's cardiovascular support requirements, levels of consciousness and tolerance levels.
- Barriers to implementation of ERM in paediatric intensive care units (PICUs) have been described in North America but little is known about these within the UK NHS setting.

What does this study add?

- Despite positivity towards the concept of ERM, less than 20% of UK PICUs currently have an established ERM protocol to define ERM content and practice.
- ERM initiation and delivery is collaborative, but there is wide variability on which patients can receive ERM and when this should be initiated.
- The provision of a standardised protocol which sets out safe and defined ERM activities along with implementation support would tackle modifiable barriers of intervention delivery
- The most important barriers to ERM delivery in PICUs within the NHS are financial resource and staffing, lack of protocols for patient selection and ERM guidelines.

Confidential: For Review Only

References

1. PICANet. Paediatric Intensive Care Audit Network National Annual Report 2016 - 2018. 2019. https://www.picanet.org.uk/annual-reporting-and-publications/ (accessed 15th September 2020).
2. Lopes-Junior LC, Rosa M, Lima RAG. Psychological and Psychiatric Outcomes Following PICU Admission: A Systematic Review of Cohort Studies. *Pediatr Crit Care Med* 2018;19(1):e58-e67. doi: 10.1097/pcc.0000000000001390 [published Online First: 2017/12/01]
3. Manning JC, Pinto NP, Rennick JE, et al. Conceptualizing Post Intensive Care Syndrome in Children-The PICS-p Framework. *Pediatr Crit Care Med* 2018;19(4):298-300. doi: 10.1097/pcc.0000000000001476 [published Online First: 2018/02/07]
4. Choong K, Awladthani S, Khawaji A, et al. Early Exercise in Critically Ill Youth and Children, a Preliminary Evaluation: The wEECYCLE Pilot Trial. *Pediatr Crit Care Med* 2017;18(11):e546-e54. doi: 10.1097/pcc.0000000000001329 [published Online First: 2017/09/19]
5. Pun BT, Balas MC, Barnes-Daly MA, et al. Caring for Critically Ill Patients with the ABCDEF Bundle: Results of the ICU Liberation Collaborative in Over 15,000 Adults. *Crit Care Med* 2019;47(1):3-14. doi: 10.1097/ccm.0000000000003482 [published Online First: 2018/10/20]
6. Fink EL, Beers SR, Houtrow AJ, et al. Early Protocolized Versus Usual Care Rehabilitation for Pediatric Neurocritical Care Patients: A Randomized Controlled Trial. *Pediatr Crit Care Med* 2019 doi: 10.1097/pcc.0000000000001881. [published Online First: 2019/02/02]
7. Nydahl P, Sricharoenchai T, Chandra S, et al. Safety of Patient Mobilization and Rehabilitation in the Intensive Care Unit. Systematic Review with Meta-Analysis. *Ann Am Thorac Soc* 2017;14(5):766-77. doi: 10.1513/AnnalsATS.201611-843SR. [published Online First: 2017/02/24]
8. National Institute for Health and Care Excellence. Rehabilitation after critical illness in adults [NICE guidance CG83]. 2009 25 March 2009. https://www.nice.org.uk/guidance/CG83 (accessed 15th September 2020).
9. Wieczorek B, Burke C, Al-Harbi A, et al. Early mobilization in the pediatric intensive care unit: a systematic review. *J Pediatr Intensive Care* 2015;2015:129-70. doi: 10.1055/s-0035-1563386 [published Online First: 2015/09/18]
10. Cuello-Garcia CA, Mai SHC, Simpson R, et al. Early Mobilization in Critically Ill Children: A Systematic Review. *The Journal of pediatrics* 2018;203:25-33.e6. doi: 10.1016/j.jpeds.2018.07.037 [published Online First: 2018/09/03]
11. Choong K, Zorko D, Awojoodu R, et al. Prevalence of acute rehabilitation for kids in the picu: A canadian multicenter point prevalence study (park-picu canada). *Pediatr Crit Care Med* 2021;22:68-69.
12. Ista E, Scholefield B, Manning J, et al. Physical rehabilitation in critically ill children: A european point prevalence study. *Pediatr Crit Care Med* 2021;22:65.
13. Mayring P. Qualitative Content Analysis. *2000* 2000;1(2) doi: 10.17169/fqs-1.2.1089 [published Online First: 2000-06-30]
14. Bengtsson M. How to plan and perform a qualitative study using content analysis. *NursingPlus Open* 2016;2:8-14. doi: https://doi.org/10.1016/j.npls.2016.01.001
15. Vaismoradi M, Turunen H, Bondas T. Content analysis and thematic analysis: Implications for conducting a qualitative descriptive study. *Nurs Health Sci* 2013;15(3):398-405. doi: 10.1111/nhs.12048. Epub 2013 Mar 11. [published Online First: 2013/03/14]
16. Burnard P. A method of analysing interview transcripts in qualitative research. *Nurse Education Today* 1991;11(6):461-66. doi: https://doi.org/10.1016/0260-6917(91)90009-Y
17. Hodgson CL, Berney S, Harrold M, et al. Clinical review: Early patient mobilization in the ICU. *Critical Care* 2013;17(1):207. doi: 10.1186/cc11820

Confidential: For Review Only

18. Cameron S, Ball I, Cepinskas G, et al. Early mobilization in the critical care unit: A review of adult and pediatric literature. *J Crit Care* 2015;30(4):664-72. doi: 10.1016/j.jcrc.2015.03.032 [published Online First: 2015/05/20]
19. Hayes L, Shaw S, Pearce MS, et al. Requirements for and current provision of rehabilitation services for children after severe acquired brain injury in the UK: a population-based study. *Archives of Disease in Childhood* 2017;102(9):813. doi: 10.1136/archdischild-2016-312166
20. Anekwe DE, Koo KK, de Marchie M, et al. Interprofessional Survey of Perceived Barriers and Facilitators to Early Mobilization of Critically Ill Patients in Montreal, Canada. *J Intensive Care Med* 2017:885066617696846. doi: 10.1177/0885066617696846. [published Online First: 2017/03/31]
21. Choong K, Koo KK, Clark H, et al. Early mobilization in critically ill children: a survey of Canadian practice. *Critical care medicine* 2013;41(7):1745-53. doi: 10.1097/CCM.0b013e318287f592 [published Online First: 2013/03/20]
22. Wang J, Xiao Q, Zhang C, et al. Intensive care unit nurses' knowledge, attitudes, and perceived barriers regarding early mobilization of patients. *Nurs Crit Care* 2020 doi: 10.1111/nicc.12507. [published Online First: 2020/04/15]
23. Curry A, Brown A, Williams S, et al. Impact of an early mobility program in the hematologic oncologic PICU setting. *Crit Care Med* 2021;49(1):527.
24. Nelliot A, Awojoodu R, Walker T, et al. Physical Rehabilitation in Critically Ill Children: A Multicenter Point Prevalence Study in the United States. *Crit Care Med* 2020:634-44.
25. Fontela PC, Forgiarini LA, Jr., Friedman G. Clinical attitudes and perceived barriers to early mobilization of critically ill patients in adult intensive care units. *Rev Bras Ter Intensiva* 2018;30(2):187-94. doi: 10.5935/0103-507x.20180037 [published Online First: 2018/07/12]
26. Goddard SL, Lorencatto F, Koo E, et al. Barriers and facilitators to early rehabilitation in mechanically ventilated patients-a theory-driven interview study. *J Intensive Care* 2018;6:4. doi: 10.1186/s40560-018-0273-0. eCollection 2018. [published Online First: 2018/02/07]
27. Zheng K, Sarti A, Boles S, et al. Impressions of Early Mobilization of Critically Ill Children-Clinician, Patient, and Family Perspectives. *Pediatr Crit Care Med* 2018;19(7):e350-e57. doi: 10.1097/PCC.0000000000001547 [published Online First: 2018/04/13]

Confidential: For Review Only

Figure 1: Perceived Benefits of Early Rehabilitation and Mobilisation
Ranking of participants potential top 5 perceived benefits of delivering ERM within PICU's. Sum of rank score: Ranking of top 5 (1-5) (1st placed rank scored 5 points to 5th placed scored 1 point). 121/124 (98%) participants ranked scores.

Figure 2: Perceived Barriers of Early Rehabilitation and Mobilisation
Institutional, Patients and Provider barriers to ERM. Figure 2 shows the percentage of responses for categories strongly agree, agree, neutral, disagree and strongly disagree. Responses ranked on the cumulative score of percentage 'strongly agree and agree'.

Confidential: For Review Only

Supplementary Material File 1

PERMIT Online Survey

Paediatric Early Rehabilitation and Mobilisation during InTensive care (PERMIT) Survey

1. Introduction

Thank you for your interest in completing a questionnaire as part of the PERMIT Study.

Why have I been invited to take part?
You have been invited to take part because you are a health care practitioner responsible for the delivery of care to children in a PICU. We would like the views of a wide range of health care professionals, and you have been identified either through the collaborating Paediatric Intensive Care Society, Physiotherapy and Occupational therapy membership lists or recommended by lead clinicians in your institutions.

Study Overview
We are conducting research into Early Rehabilitation and Mobilisation (ERM) during paediatric critical illness in the UK and assessing the feasibility and design of a future clinical trial. ERM can encompass patient-tailored interventions or bundles of care provided within the critical care unit. Rehabilitation has been shown to improve quality of life and patient outcomes in the adult ICU; however, there is limited evidence in the PIC patient population and setting.

The whole PERMIT programme aims to assess key questions regarding the acceptability and feasibility of ERM in PIC and form recommendations for a future clinical trial to establish its effectiveness.

In this questionnaire, we would like to find out:
- What forms of ERM are provided in your PICs and to which patients;
- What resources are available (or could be available) to deliver ERM;
- Your views on evaluating ERM interventions;
- Whether you would be interested in a future clinical trial to assess ERM.

Thank you for your time (which we calculated should be less than 20 minutes) in completing this questionnaire.

Barney Scholefield & Jacqueline Thompson

Confidential: For Review Only

On behalf of the PERMIT study team and the Paediatric Intensive Care Society -Study Group (PICS-SG)

2. A little demographic information about you please

1. Which hospital do you work at? *

Other (if not listed)

2. Which best reflects your current professional position? *

- [ ] Medical
- [ ] Nurse
- [ ] Physiotherapist
- [ ] Occupational therapist
- [ ] Speech and Language therapist
- [ ] Dietician
- [ ] Play therapist
- [ ] Psychologist
- [ ] Other (please specify):

3. Number of years experience working in PICU? *

- [ ] <1 year
- [ ] 1 to <5 years
- [ ] 5 to <10 years
- [ ] 10 to <15 years
- [ ] 15 to <20 years

Confidential: For Review Only

- [ ] More than 20 years
- [ ] I do not work in PICU

Comments:

3. What ERM do you currently provide in your PICU?

In the following questions, we hope to explore your current practice related to early rehabilitation and mobilisation (ERM). We understand that the term ERM may mean different things to different professional groups; however, we are keen to capture the current diverse views and how ERM is defined by you and your PICU team.

4. Please describe in the box below the current ERM practice within your PICU. (We are especially interested in whether you provide anything that you feel fits within the term ERM, who receives ERM, what specific actions are involved in ERM and who delivers the ERM).

5. Which of the following best describes when you would consider ERM for a patient? *

- [ ] Within 24 hours of admission to PICU
- [ ] About 2 to 3 days after PICU admission
- [ ] Around the end of the first week of PICU admission
- [ ] Before discharge from PICU
- [ ] As soon as the patient is stable enough
- [ ] As soon as the patient is off mechanical ventilatory support
- [ ] To start earlier than we historically considered starting rehabilitation and mobilisation
- [ ] We do not consider ERM for our PICU patients
- [ ] I don't know
- [ ] Other (please specify):

Confidential: For Review Only

Comments:

6. Which team members are involved in ERM in your PICU? (Please select how frequently they are involved. If the team member is not available in your PICU please select 'Not applicable')

	Always	Very often	Sometimes	Seldom	Never	Not applicable (as team member is not available in my PICU)	Don't know
Medical staff	☐	☐	☐	☐	☐	☐	☐
Nursing staff	☐	☐	☐	☐	☐	☐	☐
Speech and language therapist	☐	☐	☐	☐	☐	☐	☐
Physiotherapist	☐	☐	☐	☐	☐	☐	☐
Occupational therapist	☐	☐	☐	☐	☐	☐	☐
Psychologist	☐	☐	☐	☐	☐	☐	☐
Play therapist	☐	☐	☐	☐	☐	☐	☐
Dietician	☐	☐	☐	☐	☐	☐	☐
Patients family or carers	☐	☐	☐	☐	☐	☐	☐
Other	☐	☐	☐	☐	☐	☐	☐

Please define 'other' or add any comments

Confidential: For Review Only

7. How often do patients, in general, receive ERM in your PICU?

	Always	Very often	Sometimes	Seldom	Never	Don't know
All PICU patients	☐	☐	☐	☐	☐	☐

Comments:

8. How often do patients in the following age groups receive ERM in your PICU? (Please select an option for each)

	Always	Very often	Sometimes	Seldom	Never	Don't know
Infants & <1 year old	☐	☐	☐	☐	☐	☐
1-4 years old	☐	☐	☐	☐	☐	☐
> 4-10 years old	☐	☐	☐	☐	☐	☐
> 10-18 years old	☐	☐	☐	☐	☐	☐

Comments:

9. How often do patients within the following diagnostic categories receive ERM in your PICU? (Please select an option for each)

	Always	Very often	Sometimes	Seldom	Never	Don't know
Acquired brain injury patients (e.g. TBI, meningitis)	☐	☐	☐	☐	☐	☐
Sepsis	☐	☐	☐	☐	☐	☐
Congenital heart disease	☐	☐	☐	☐	☐	☐
Cancer	☐	☐	☐	☐	☐	☐
Mechanically ventilated	☐	☐	☐	☐	☐	☐

Confidential: For Review Only

	Always	Very often	Sometimes	Seldom	Never	Don't know
Mechanically supported (e.g. ECMO)	☐	☐	☐	☐	☐	☐
Respiratory illness (bronchiolitis or pneumonia)	☐	☐	☐	☐	☐	☐
Multi-organ failure	☐	☐	☐	☐	☐	☐
With pre-existing physical co-morbidity	☐	☐	☐	☐	☐	☐
With pre-existing severe developmental delay	☐	☐	☐	☐	☐	☐

Comments:

10. How often do patients, with the following length of stay, receive ERM in your PICU? (Please select an option for each)

	Always	Very often	Sometimes	Seldom	Never	Don't know
Patients in PICU <3 days	☐	☐	☐	☐	☐	☐
Patients in PICU 3-7 days	☐	☐	☐	☐	☐	☐
Patients in PICU >7-28 days	☐	☐	☐	☐	☐	☐
Patients in PICU >28 days	☐	☐	☐	☐	☐	☐

Comments:

11. Who decides if the patient can start to receive ERM? (Please select all that apply)

☐ Medical consultant

☐ Other member of the medical team (e.g. PICU trainee/ advanced nurse practitioner)

Confidential: For Review Only

- ☐ Bedside nurse
- ☐ Senior nurse on shift
- ☐ Physiotherapist
- ☐ Occupational therapist
- ☐ Parent(s)
- ☐ Don't know
- ☐ Not applicable
- ☐ Other (please specify):

Comments:

12. How does your PICU assess whether patients tolerate and cope with the therapy provided? (eg monitor safety)

13. Do you currently use or have an established guideline or protocol regarding management of ERM in your PICU?

- ☐ No
- ☐ Yes - if you are able to share, please email to b.scholefield@bham.ac.uk
- ☐ Don't know

Comments:

4. Question regarding your established ERM programme or protocol

14. What do you have within your protocolised ERM treatment? (Please select all that apply)

Confidential: For Review Only

- [ ] Physical therapy not requiring additional equipment
- [ ] Physical therapy requiring additional equipment
- [ ] Occupational therapy
- [ ] Speech and language therapy
- [ ] Psychology
- [ ] Delirium screening
- [ ] We do not have a programme or protocol related to ERM activities
- [ ] Other (please specify):

Comments:

15. What are the key factors in successfully implementing your ERM protocol?

5. ERM related processes and equipment in your PICU

The following three questions are related to additional processes or equipment in your PICU related to ERM activities.

16. Does your PICU have a protocol or guidelines for the management of the following?

	Yes	No	Don't know
Sedation	☐	☐	☐
Analgesia	☐	☐	☐
Drug/ opioid withdrawal	☐	☐	☐
Delirium	☐	☐	☐

Confidential: For Review Only

	Yes	No	Don't know
Mobilisation (at any time point)	☐	☐	☐

17. What types of therapies or activities are available in your PICU? (Please select all that apply)

- ☐ Physical therapy - not requiring additional equipment (e.g. passive or active movements)
- ☐ Physical therapy - requiring additional equipment (e.g. in bed cycling)
- ☐ Occupational therapy
- ☐ Speech and language therapy
- ☐ Clinical psychology
- ☐ Delirium screening
- ☐ Patient diaries
- ☐ Play therapy
- ☐ Pet therapy
- ☐ Music therapy
- ☐ Other (please specify):

18. What equipment does your PICU have available for physical therapy/mobilisation? (Please select all that apply)

- ☐ Tilt table
- ☐ Bed with Trendelenburg features
- ☐ Bed with full chair position
- ☐ Bed with chair egress - exit out the foot of the bed
- ☐ Speciality bed with continuous side to side rotation
- ☐ Bed with retractable footboard i.e. flex a foot feature

Confidential: For Review Only

- [ ] Mobile lifts
- [ ] Ceiling lifts
- [ ] Portable ventilators
- [ ] Patient rolling walker
- [ ] Bedside cycle (patient can remain in bed)
- [ ] Transcutaneous electrical stimulation of muscles
- [ ] Specialist wheelchair
- [ ] Specialist static seating i.e. tumble forms
- [ ] Other (please specify):

6. Preferences, barriers and outcomes

For the following questions, we would explore your strength of opinion on ERM as a therapy, potential barriers to using ERM in practice and which are the most important clinical outcomes to consider when assessing if ERM is beneficial.

19. Please select one option regarding your current views of ERM in your PICU? *

- [ ] Crucial, should be the top priority in the care of PICU patients
- [ ] Very important, should be a priority in the care of PICU patients
- [ ] Important, should be a priority in the care of PICU patients
- [ ] Somewhat important, should be considered in the care of PICU patients
- [ ] Not of great importance, clinicians should bear it in mind in the care of PICU patients
- [ ] Of minimal importance to the care of PICU patients
- [ ] Of no importance to the care of PICU patients

Comments:

Confidential: For Review Only

20. Below are potential institutional barriers to the delivery of ERM. Please select to what extent you agree/disagree that each are barriers to delivery of ERM in your PICU. *

	Strongly Agree	Agree	Neutral	Disagree	Strongly Disagree
Instructions that patients should not move in their bed	☐	☐	☐	☐	☐
Consultant's permission or prescription is required prior to mobilisation	☐	☐	☐	☐	☐
Insufficient equipment/ resources	☐	☐	☐	☐	☐
Inadequate funding	☐	☐	☐	☐	☐
Lack of written guidelines/ protocol	☐	☐	☐	☐	☐
Lack of physical space	☐	☐	☐	☐	☐
Absence of champion/ advocate to promote ERM	☐	☐	☐	☐	☐
Absence of frequent (e.g. daily) ERM patient screening	☐	☐	☐	☐	☐

Comments:

Confidential: For Review Only

21. Below are potential patient barriers to the delivery of ERM. Please select to what extent you agree/disagree that each are barriers to delivery of ERM in your PICU. *

	Strongly Agree	Agree	Neutral	Disagree	Strongly Disagree
Physiological instability ('the patient is just too sick')	☐	☐	☐	☐	☐

	Strongly Agree	Agree	Neutral	Disagree	Strongly Disagree
Sedation level	☐	☐	☐	☐	☐
Presence of endotracheal intubation	☐	☐	☐	☐	☐
Risk of device/ line/ catheter dislodgement	☐	☐	☐	☐	☐
Cognitive impairment/ age	☐	☐	☐	☐	☐
Obesity	☐	☐	☐	☐	☐
Patient motivation	☐	☐	☐	☐	☐
Family concerns	☐	☐	☐	☐	☐

Comments:

22. Below are potential provider barriers to the delivery of ERM. Please select to what extent you agree/disagree that each are barriers to delivery of ERM in your PICU. *

	Strongly Agree	Agree	Neutral	Disagree	Strongly Disagree
Lack of prioritisation of ERM within patient care plans	☐	☐	☐	☐	☐
Limited staffing to deliver ERM	☐	☐	☐	☐	☐
Lack of coordination within and between clinician groups	☐	☐	☐	☐	☐
Slow recognition of patient readiness for ERM	☐	☐	☐	☐	☐
Conflicting perceptions concerning patient suitability	☐	☐	☐	☐	☐

Confidential: For Review Only

	Strongly Agree	Agree	Neutral	Disagree	Strongly Disagree
Safety concerns	☐	☐	☐	☐	☐
Lack of specific decision-making authority for ERM initiation	☐	☐	☐	☐	☐
Inadequate training	☐	☐	☐	☐	☐
Prolonging the current working day	☐	☐	☐	☐	☐

Comments:

23. Please rank what you perceive to be the top 5 most important benefits that ERM could provide in PICU. (Please select, in order, the top perceived benefits by selecting a box). *

	1 (most important)	2	3	4	5 (least important)
Reduction in treatment cost	☐	☐	☐	☐	☐
Reduction in patient delirium	☐	☐	☐	☐	☐
Reduction in length of ICU stay	☐	☐	☐	☐	☐
Reduction in days requiring mechanical ventilation	☐	☐	☐	☐	☐
Reduction in the rate of pulmonary complications	☐	☐	☐	☐	☐
Reduction in readmission (hospital and PICU)	☐	☐	☐	☐	☐
Increase in number of patients discharged home	☐	☐	☐	☐	☐
Improved patient satisfaction	☐	☐	☐	☐	☐

Confidential: For Review Only

	1 (most important)	2	3	4	5 (least important)
Improved staff satisfaction	☐	☐	☐	☐	☐
Improved family satisfaction	☐	☐	☐	☐	☐
Improvement in patient sleep quality	☐	☐	☐	☐	☐
Improvement in psychological impact of PICU care	☐	☐	☐	☐	☐
Improvement in daily life participation following discharge	☐	☐	☐	☐	☐

Comments:

7. Final thoughts

24. Would you be willing to support a future clinical trial in the UK and Ireland based on evaluating ERM practice?

☐ Yes

☐ No

☐ Don't Know

Comments:

25. Would you allow us to contact you in the future with information regarding workshops we will be running to discuss ERM practice and trial feasibility, please? *

☐ Yes (please click the link on the next page to provide best contact information, thank you!)

☐ No

Confidential: For Review Only

Funding statement: This study/project is funded by the National Institute for Health Research (NIHR) [Health Technology Assessment Programme (project reference: 17/21)/ HTA]. The views expressed are those of the author(s) and not necessarily those of the NIHR or the Department of Health and Social Care.

Confidential: For Review Only

Supplementary Material File 2

Table 1: Descriptions of ERM

Confidential: For Review Only

Category	Sub-categories	Examples:
**Activity-focused**<br>ERM is activity-focused, with consideration of seating and equipment, primarily delivered by nursing and allied health care professionals with optimised sedation management to promote patient engagement.<br>There is a lack of consensus on what is routine care compared with purposeful ERM activity.	Activity – mobilising, positioning, stretching	"ERM provided includes - passive movements of limbs, periodic change of position, use of splints on extremities and if stable in the long term patients - sitting up in tumble form chair and mobilising out of bed." (Doctor, 67)<br><br>"We aim to get the children sitting upright, either over the edge of the bed or inappropriate seating as soon as possible." (Occupational Therapist, 001)<br><br>"Nursing staff are taught the importance of regular position changes for patient ... and positioning to maintain ranges of movement and prevent foot drop." (Nurse, 017)
	Core health care professional involvement – physiotherapist, nurse, occupational therapist	"We try and mobilise patients as soon as able when not invasively ventilated - physio led." (Doctor, 058)<br><br>"As physiotherapists, we work with the occupational therapist and nursing staff to either re-position, sit up in bed, sit on the edge of the bed or stand whilst on the ventilators."(Physiotherapist, 069)
	Additional health care professional involvement – psychology, dietician, play therapist, speech and language therapists	"Speech and language therapy become involved usually when nursing or medical staff identify a need for referral." (Doctor, 003)<br><br>"We have a Play Specialist on PICU, who assists with communication tools/toys." (Nurse, 082)
	Seating and equipment	"Specialist seating need – linking in with OT to ensure appropriate seating available." (Physiotherapist, 019)

		"We have also in the past asked adult [services] to use their moto-med bike and used this with teenagers, but this can be a challenge as it is very far from PICU and it is often in use." (Physiotherapist, 014)
	Sedation management	"Working with the medics to wean sedation as quickly as possible. This promotes faster ability to mobilise and progress in their rehabilitation." (Nurse, 034)
	'Routine care' vs 'purposeful ERM package'	Some centres define ERM as passive range of movement, repositioning or providing splints. We would deem this as essential core cares rather than ERM; the large majority of our patients will have positioning and movement plan from day 1 of admission." (Physiotherapist, 111)
**Tailored**<br>Following the assessment of needs and patient and family preference, ERM activities are personalised to the individual patient.	Assessment of need	"We have a programme where patients are categorised to one of 3 levels. Each level provides nursing and therapy staff with activities aligned to the acuity of the patient." (Nurse, 040)
	Individual preference	"Parents will tell us what their child enjoys doing and make suggestions, often provide toys/games from home." (Nurse, 076)<br><br>"Discuss with parents what patient enjoys doing, watching etc. Discuss options regarding taking patients 'out' where possible if long term patients." (Nurse, 015)
**Promote recovery**<br>The purpose of ERM is to normalise the PICU environment, to create an environment that addresses holistic needs, sustains or promotes development and supports recovery from critical illness.	Normalising PICU environment	"Provide activities they would use for their enjoyment. This enables them to be more relaxed and less aware of what is going on with / around them." (Play specialist, 102)<br><br>"Being in PICU can be a frightening experience. Not only is a child/young person away from home, but also away from their usual environment, family and friends. There are unfamiliar sounds, smells, equipment and people. The play specialist can help to make the stay much more enjoyable and children/young people to cope and understand." (Play specialist, 055)
	Sustain / promote child development	"Encouraging the patient to regain or further their development in a therapeutic fun manner." (Play therapist,102)

Confidential: For Review Only

	Restoration / recovery	"Interventions aim to promote physical recovery – movement, and ability to engage in activities and psychological recovery – orientation, speech, ability to play and attend school. " (Nurse, 021)
**Timing of ERM**<br>The optimal timing of ERM initiation is challenging for health care professionals to define, although likelihood perceived to increase with increased length of stay. Influenced by the perceived stability of patients and the balance of risk to benefit.	'Early' poorly defined	"I like to think we consider it within 2-3 days we have a better idea of the patients PICU journey and how long they are going to stay. The reality is it's usually longer... usually week 2 of admission if they are still on the unit." (Physiotherapist, 090)<br><br>"There is no formal plan for who decides if a patient should start ERM and when… Currently the delivery of ERM is fairly ad hoc, based on what is highlighted from daily handover each morning." (Physiotherapist, 083)<br><br>"We provide ERM as a structured programme… every patient after 24 hours of admission is considered for ERM." (Doctor, 012)
	Patient stability, risk and benefit	"Members of the PICU team with less experience of early rehab can deem ERM 'unsafe' which can occasionally act as a barrier."(Physiotherapist, 016)<br><br>"Usually ERM activity is not considered until patients can physiologically tolerate movement and are stable with observations." (Nurse, 008)
	'Long-stay'	"ERM is often thought about as a patient comes up to extubation or has failed extubation, and we feel that the reason for failure may be because of critical care weakness." (Physiotherapist, 031)<br><br>"We provide very little ERM on PICU, other than for long term patients, which is often, in my opinion, delayed." (Nurse, 007)

Confidential: For Review Only

Table 2: Reported frequency of receiving or being involved in Early Rehabilitation and Mobilisation by age groups, length of PICU stay, diagnostic category, and health care professional or parental/family role (n=124 respondents)*

By age group	Always (n, %)	Very often (n, %)	Sometimes (n, %)	Seldom (n, %)	Never (n, %)	Don't know (n, %)	Not Applicable[#] n
All PICU Patients (any age)	6 (5)	45 (36)	48 (39)	14 (11)	0 (0)	11 (9)	0
>10-18 years old	18 (15)	40 (32)	43 (35)	8 (6)	1 (1)	14 (11)	0
>4-10 years old	16 (13)	42 (34)	41 (33)	10 (8)	1 (1)	14 (11)	0
1-4 years old	13 (10)	42 (34)	39 (31)	13 (10)	1 (1)	16 (13)	0
Infants & <1 year old	6 (5)	47 (38)	32 (26)	20 (16)	3 (2)	16 (13)	0
**By length of stay in PICU**							
PICU >28 days	43 (35)	48 (39)	14 (11)	4 (3)	0 (0)	15 (12)	0
PICU >7-28 days	27 (22)	46 (37)	26 (21)	8 (6)	0 (0)	17 (14)	0
PICU 3-7 days	12 (10)	32 (26)	40 (32)	19 (15)	5 (4)	16 (13)	0
PICU <3 days	3 (2)	14 (11)	39 (31)	33 (27)	17 (14)	18 (15)	0
**By diagnosistic category**							
Acquired brain injury	36 (29)	39 (31)	16 (13)	11 (9)	4 (3)	18 (15)	0
Severe developmental delay	11 (9)	43 (35)	39 (31)	12 (10)	3 (2)	16 (13)	0
Cancer	11 (9)	24 (19)	36 (29)	14 (11)	4 (3)	35 (28)	0
Pre-existing physical morbidity	9 (7)	38 (31)	44 (35)	12 (10)	4 (3)	17 (14)	0
Mechanically ventilated	9 (7)	38 (31)	39 (31)	18 (15)	5 (4)	15 (12)	0
Congenital heart disease	9 (7)	24 (19)	39 (31)	15 (12)	6 (5)	31 (25)	0
Respiratory illness	7 (6)	35 (28)	39 (31)	22 (18)	3 (2)	18 (15)	0
Sepsis	7 (6)	28 (23)	44 (35)	20 (16)	5 (4)	20 (16)	0
Multi-organ failure	5 (4)	14 (11)	32 (26)	35 (28)	12 (10)	26 (21)	0
Mechanically supported (e.g. extracorporeal life support)	4 (3)	8 (6)	14 (11)	13 (10)	47 (38)	38 (31)	0
**Health Care Professional team members & parent or family member(s) involvement in ERM (when applicable[#])**							
Physiotherapist	86 (70)	27 (22)	4 (3)	2 (2)	0 (0)	4 (3)	1

Confidential: For Review Only

Nurse	54 (44)	49 (40)	9 (7)	5 (4)	0 (0)	6 (5)	1
Parent or family member	36 (29)	56 (46)	19 (15)	7 (6)	1 (1)	4 (3)	1
Occupational therapist	23 (19)	34 (29)	29 (24)	20 (17)	6 (5)	7 (6)	5
Doctor	22 (18)	20 (16)	26 (21)	30 (24)	13 (11)	12 (10)	1
Play therapist	12 (10)	24 (21)	39 (34)	26 (23)	9 (8)	5 (4)	9
Dietician	10 (8)	20 (17)	12 (10)	29 (24)	37 (31)	13 (11)	3
Speech and Language therapist	4 (3)	11 (9)	40 (33)	35 (29)	20 (17)	10 (8)	4
Psychologist	3 (3)	11 (10)	24 (23)	32 (30)	30 (28)	6 (6)	18

* Percentages may not total 100 due to rounding

# Not applicable (e.g. not available in PICU) responses excluded from percent calculation.

Confidential: For Review Only

Table 3: Barriers of ERM delivery within Paediatric Intensive Care Units (n=124 respondents)*

Institutional barriers	Strongly Agree n (%)	Agree n (%)	Neutral n (%)	Disagree n (%)	Strongly Disagree n (%)	Skipped responses[#] (n)
Insufficient equipment/resources	31 (26)	52 (43)	22 (18)	13 (11)	3 (2)	3
Lack of written guidelines/protocol	31 (26)	38 (31)	35 (29)	11 (9)	6 (5)	3
Inadequate funding	30 (25)	43 (36)	28 (23)	16 (13)	4 (3)	3
Absence of champion/advocate to promote ERM	25 (21)	43 (36)	23 (19)	21 (17)	9 (7)	3
Absence of frequent ERM patient screening	25 (23)	38 (35)	24 (22)	14 (13)	8 (7)	14
Lack of physical space	17 (14)	51 (42)	17 (14)	32 (26)	4 (3)	3
Instructions that patients should not move in their bed	10 (8)	25 (21)	24 (20)	48 (40)	14 (12)	3
Consultant's permission or prescription is required prior to mobilisation	5 (4)	39 (32)	32 (26)	33 (27)	12 (10)	3
**Provider barriers**						
Slow recognition of patient readiness for ERM	19 (16)	46 (38)	19 (16)	35 (29)	2 (2)	3
Limited staffing to deliver ERM	42 (35)	54 (45)	11 (9)	13 (11)	1 (1)	3
Lack of prioritisation of ERM within patient care plans	27 (22)	45 (37)	22 (18)	24 (20)	3 (2)	3
Inadequate training	20 (17)	54 (45)	18 (15)	26 (21)	3 (2)	3
Conflicting perceptions concerning patient suitability	17 (14)	59 (49)	22 (18)	21 (17)	2 (2)	3
Lack of specific decision-making authority for ERM initiation	16 (13)	49 (40)	28 (23)	24 (20)	4 (3)	3
Safety concerns	15 (12)	55 (45)	32 (26)	18 (15)	1 (1)	3
Lack of coordination within and between clinician groups	14 (12)	44 (37)	30 (25)	27 (23)	5 (4)	4
Prolonging the current working day	4 (3)	21 (17)	43 (36)	41 (34)	12 (10)	3
**Patients barriers**						
Levels of physiological instability	48 (40)	53 (44)	12 (10)	7 (6)	1 (1)	3
Sedation level	27 (22)	64 (53)	15 (12)	13 (11)	2 (2)	3
Presence of endotracheal intubation levels	8 (7)	36 (30)	32 (26)	39 (32)	6 (5)	3
Risk of device/line/catheter dislodgement levels	8 (7)	42 (35)	32 (26)	35 (29)	4 (3)	3
Cognitive impairment/age levels	1 (1)	32 (26)	29 (24)	46 (38)	13 (11)	3
Obesity levels	6 (5)	14 (12)	35 (29)	52 (43)	14 (12)	3

Confidential: For Review Only

Patient motivation level	2 (2)	38 (31)	31 (26)	43 (36)	7 (6)	3
Family concerns levels	2 (2)	42 (35)	35 (29)	38 (31)	4 (3)	3

* Percentages may not total 100 due to rounding # Skipped responses excluded from percent calculation.

Confidential: For Review Only

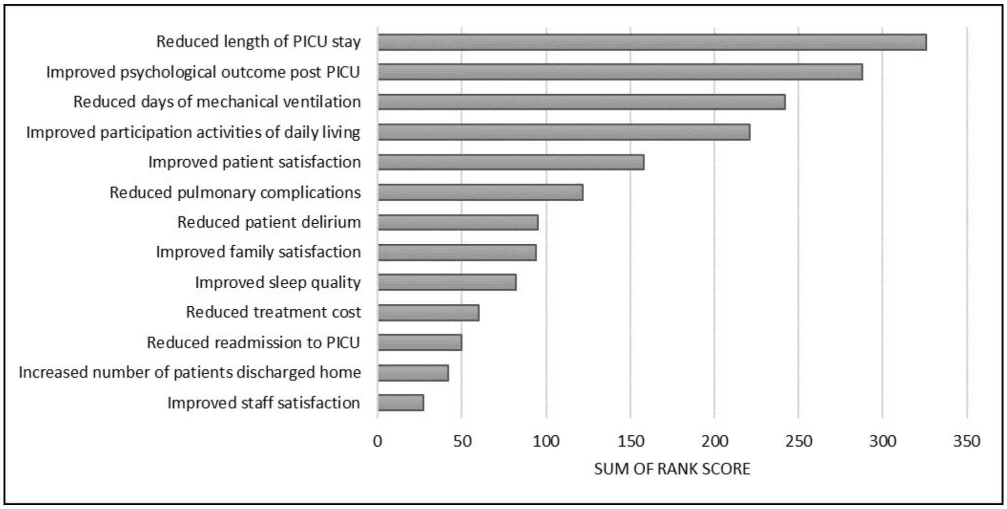

88x44mm (300 x 300 DPI)

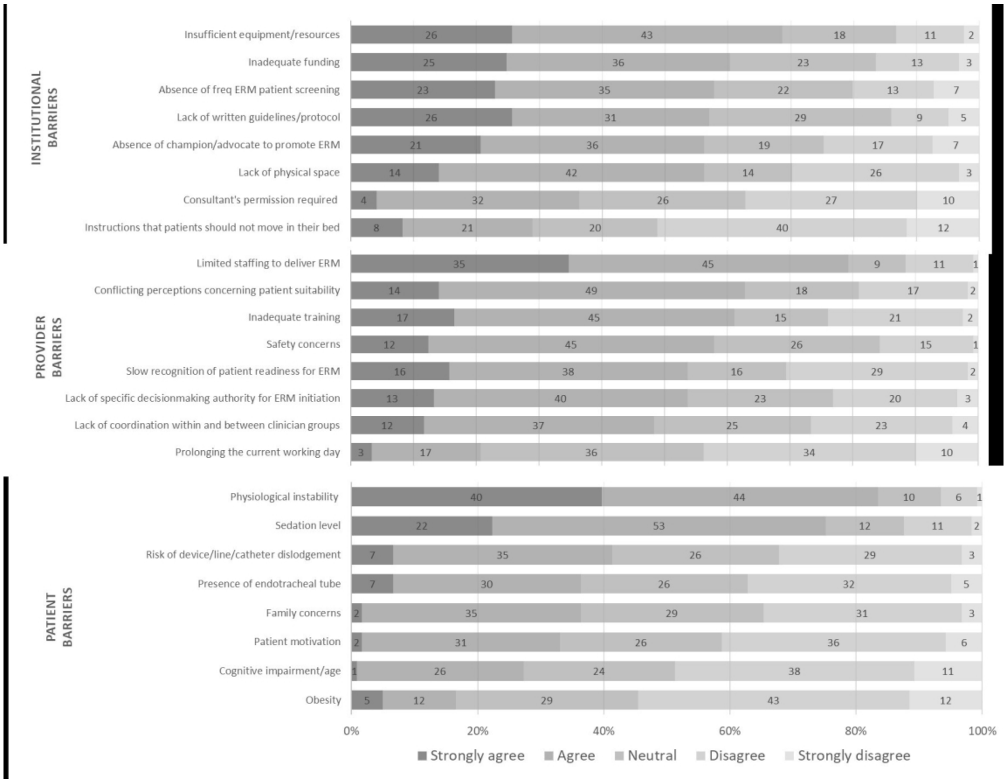

153x119mm (300 x 300 DPI)

BMJ Paediatrics Open

Early Mobilisation and Rehabilitation in the PICU: A UK survey

Journal:	*BMJ Paediatrics Open*
Manuscript ID	bmjpo-2021-001300.R3
Article Type:	Original research
Date Submitted by the Author:	07-Feb-2022
Complete List of Authors:	Thompson, Jacqueline; University of Birmingham, ; University of Birmingham<br>Menzies, Julie; Birmingham Women's and Children's NHS Foundation Trust, Paediatric Intensive Care<br>Manning, Joseph ; Nottingham University Hospitals NHS Trust, Nottingham Children's Hospital; University of Nottingham, Children and Young people Health Research, School of Health Sciences<br>McAnuff, Jennifer; Newcastle University, Population Health Sciences Institute, Newcastle University; Northumbria University, Department of Social Work, Education and Community Wellbeing<br>Brush, Emily; University of Birmingham<br>Ryde, Francesca; University of Birmingham<br>Rapley, Tim; Northumbria University<br>Pathan, Nazima; University of Cambridge, Paediatrics<br>Brett, Stephen; Imperial College of Science Technology and Medicine, Department of surgery and cancer<br>Moore, David; University of Birmingham<br>Geary, Michelle; University Hospital Southampton NHS Foundation Trust, Child Health<br>Colville, Gillian; St George's University Hospitals NHS Foundation Trust, Paediatric Psychology Service<br>Morris, Kevin; Birmingham Women's & Children's NHS Foundation Trust<br>PARSLOW, ROGER; UNIVERSITY OF LEEDS, PAEDIATRIC EPIDEMIOLOGY GROUP<br>Feltbower, Richard; UNIVERSITY OF LEEDS<br>Lockley, Sophie; Birmingham Children's Hospital<br>Kirkham, Fenella; Neurosciences Unit, UCL Institute of Child Health<br>Forsyth, Rob; Newcastle University, Institute of Neuroscience<br>Scholefield, Barnaby; University of Birmingham
Keywords:	Rehabilitation, Epidemiology

SCHOLARONE™
Manuscripts

I, the Submitting Author has the right to grant and does grant on behalf of all authors of the Work (as defined in the below author licence), an exclusive licence and/or a non-exclusive licence for contributions from authors who are: i) UK Crown employees; ii) where BMJ has agreed a CC-BY licence shall apply, and/or iii) in accordance with the terms applicable for US Federal Government officers or employees acting as part of their official duties; on a worldwide, perpetual, irrevocable, royalty-free basis to BMJ Publishing Group Ltd ("BMJ") its licensees and where the relevant Journal is co-owned by BMJ to the co-owners of the Journal, to publish the Work in this journal and any other BMJ products and to exploit all rights, as set out in our licence.

The Submitting Author accepts and understands that any supply made under these terms is made by BMJ to the Submitting Author unless you are acting as an employee on behalf of your employer or a postgraduate student of an affiliated institution which is paying any applicable article publishing charge ("APC") for Open Access articles. Where the Submitting Author wishes to make the Work available on an Open Access basis (and intends to pay the relevant APC), the terms of reuse of such Open Access shall be governed by a Creative Commons licence – details of these licences and which Creative Commons licence will apply to this Work are set out in our licence referred to above.

Other than as permitted in any relevant BMJ Author's Self Archiving Policies, I confirm this Work has not been accepted for publication elsewhere, is not being considered for publication elsewhere and does not duplicate material already published. I confirm all authors consent to publication of this Work and authorise the granting of this licence.

Confidential: For Review Only

Early Mobilisation and Rehabilitation in the PICU: A UK survey

Manuscript type: Original Research

Authors:

Thompson JY[1], Menzies JC[2], Manning JC[3,4], McAnuff J[5, 6], Brush E[1], Ryde F[1], Rapley TR[6], Pathan N[7], Brett S[8], Moore D[9], Geary M[10], Colville G[11], Morris KP[2], Parslow RC[12], Feltbower RG[12], Lockley S[13], Kirkham F[10], Forsyth R[14], Scholefield B R[1,2], On behalf of the PERMIT Collaborators & Paediatric Intensive Care Society Study-Group (PICS-SG)

Author affiliations:

1. Birmingham Acute Care Research Group, Institute of Inflammation and Ageing, University of Birmingham
2. Paediatric Intensive Care, Birmingham Women's & Children's NHS Foundation Trust
3. Nottingham Children's Hospital, Nottingham University Hospitals NHS Trust
4. Children and Young People Health Research, School of Health Sciences, The University of Nottingham
5. Population Health Sciences Institute, Newcastle University
6. Department of Social Work, Education and Community Wellbeing, Northumbria University
7. Department of Paediatrics, The Chancellor, Masters, and Scholars of the University of Cambridge
8. Department of surgery and cancer; Imperial College of Science, Technology and Medicine

Confidential: For Review Only

9. Institute of Applied Health, University of Birmingham

10. Child Health, University Hospital Southampton NHS Foundation Trust

11. Paediatric Psychology Service; St George's University Hospitals, NHS Foundation Trust

12. Leeds Institute for Data Analytics, School of Medicine, University of Leeds, Leeds

13. PPI Representative

14. Translational and Clinical Research Institute, Newcastle University

Corresponding author:

Barnaby R Scholefield; B.Scholefield@bham.ac.uk

Twitter handle: @BarneyUoB

Abstract word count: 264 words now

Main text word count (excluding abstract, reference list, tables, and figures): 2732

References: 27

Tables: 6

Figures: 2

Supplementary files: 2 (1 survey, 3 supplemental tables)

Confidential: For Review Only

Abstract

Objective: To understand the context and professional perspectives of delivering early rehabilitation and mobilisation (ERM) within UK Paediatric Intensive Care Units (PICU).

Design: A web-based survey administered May-August 2019.

Setting: United Kingdom PICUs.

Participants: A total of 124 staff from 26 PICUs participated, including 22 (18%) Doctors, 34 (27%) Nurses, 28 (23%) Physiotherapists, 19 (15%) Occupational therapists, and 21 (17%) were other professionals.

Results: Key components of participants' definitions of ERM included tailored, multi-disciplinary rehabilitation packages focused on promoting recovery. Multi-disciplinary involvement in initiating ERM was commonly reported. Over half of respondents favoured delivering ERM after achieving physiological stability (n=69, 56%). All age groups were considered for ERM by relevant health professionals. However, responses differed concerning the timing of initiation. Interventions considered for ERM were more likely to be delivered to patients when PICU length of stay exceeded 28 days and among patients with acquired brain injury or severe developmental delay. The most commonly identified barriers were: physiological instability (81%), limited staffing (79%), sedation requirement (73%), insufficient resources and equipment (69%), lack of recognition of patient readiness (67%), patient suitability (63%), inadequate training (61%), and inadequate funding (60%). Respondents ranked reduction in PICU length of stay 74% and improvement in psychological outcomes 73% as the most important benefits of ERM.

Confidential: For Review Only

Conclusion: ERM is gaining familiarity and endorsement in UK PICUs, but significant barriers to implementation due to limited resources and variation in content and delivery of ERM persist. A standardised protocol that sets out defined ERM interventions, along with implementation support to tackle modifiable barriers, is required to ensure the delivery of high-quality ERM.

Confidential: For Review Only

INTRODUCTION

In the UK, approximately 20,000 children are admitted to Paediatric Intensive Care Units (PICU) yearly[1], and although most recover, some develop longer-term physical, psychological and cognitive impairment[2]. These significant morbidities have been termed Post Intensive Care Syndrome in Paediatrics (PICS-p)[3]. Early rehabilitation and mobilisation (ERM) encompass patient-tailored interventions, delivered within seven days of admission, individually[4] or in a bundled package[5] to patients within intensive care settings. It is provided by health professionals from multiple disciplines and care-givers and may promote physical (e.g. functional activities)[4] and non-physical (e.g. psychological, cognitive)[6] recovery.

Within adult ICUs, ERM has been demonstrated to be safe, feasible and cost-effective[7 8]. It can shorten the length of ventilation, duration of intensive care and hospital stay with economic benefit, improve long term physical functioning and return to independence, and is recommended by National Institute for Health Care Excellence (NICE)[8]. Whilst ERM has been reported to be safe and feasible in PICUs [9-11], the patient population is different, and ERM is less well defined, leading to variability in practice. This variation in practice and components of interventions delivered has also been described in European PICU's[12]. At present, no national standardised care pathway for ERM exists within UK PICUs.

This study forms part of the National Institute for Health Research (NIHR) funded PERMIT study (NIHR HTA: 17/21/06) investigating ERM in children. This work set out to understand reported practices and perceptions of ERM within the UK PICU context.

Confidential: For Review Only

Our objectives were to:

I. Explore how health care professionals describe and administer ERM using a qualitative approach

II. Identify and describe current ERM practice using a quantitative approach

Understand and quantify perceived barriers and facilitators of ERM, presenting findings using descriptive analysis.METHODS

A web-based survey (administered through www.smartsurvey.co.uk) was developed that included 25 questions (Supplementary material file 1). A patient representative was involved in the design and development of the survey. A pilot survey was conducted among multi-disciplinary health professionals (n=40) teams within two PICUs to assess acceptability and comprehensiveness. There were very few missing responses; therefore, no questions were removed, but five questions were rephrased to add clarity. Pilot responses were excluded from the main survey analysis.

A UK Paediatric Intensive Care Society Study Group (PICS-SG) member of each UK PICU (n=29) was contacted via email and requested to identify and cascade to members of their local multi-disciplinary team (including at least one physiotherapist, doctor and nurse) to complete the survey. Participating PICUs were sent a survey link to distribute between May and August 2019. Three follow up reminders were sent at weekly intervals to non-responders.

Likert scales with the following categories (and associated scores) – always (5), very often (4), sometimes (3), seldom (2) and never (1) - were used to express the frequency of

Confidential: For Review Only

practice or level of agreement (using median and IQR of score). The ranking of perceived ERM benefits was calculated using the sum of ranked scores of respondents top 5 important benefits (5 points for 1st, reducing to 1 point for 5th placed). Statistical analysis was performed using R version x64 3.5.1 (R Foundation for Statistical Computing, Vienna, Austria).

Open-ended responses were analysed using a qualitative content analysis approach[13]. Two researchers independently familiarised themselves with the data and conducted open-coding, utilising NVIVO™ software for data management. Codes were then discussed, summarised and organised[14 15]. In relevant sections of the paper, free text quotes from respondents are reported to add context and clarity[16]. The University of Birmingham granted institutional ethical approval on 05/02/2019 (reference ERN_18-1134). Consent was implied through survey completion.

Patient and Public Involvement

The pilot survey received input from multi-disciplinary health professionals who were members of the UK Paediatric Intensive Care Society Study Group (PICS-SG). We also recruited a patient representative as part of the study team to ensure the perspective, experiences and preferences of children admitted to PICU were incorporated into the design and development of the survey. The patient representative was a parent of a child who had been admitted to PICU. They both contributed to the nature of questions considered in the survey to ensure the study outcome would be relevant to parents and members of the PICS-SG on dissemination.

Confidential: For Review Only

RESULTS

Demographics

We received responses from PICS-SG link members in 26/29 (90%) UK PICUs. A total of 191 healthcare professionals opened the survey link, with 124 (65%) submitting responses.

Table 1: Characteristics of survey respondents (n=124 respondents)

**Professional group**	**n (%)***
Nurse	34 (27)
Physiotherapist	28 (23)
Medical doctor (consultant)	22 (18)
Occupational therapist	19 (15)
Play therapist	7 (6)
Psychologist	7 (6)
Dietician	6 (5)
Speech and Language therapist	1 (1)
**Years of experience**	**n (%)***
<1 year	7 (6)
1 to <5 years	27 (22)
5 to <10 years	30 (24)
10 to <15 years	14 (11)
15 to <20 years	33 (27)
More than 20 years	15 (12)

* Percentages may not total 100 due to rounding

As shown in Table 1, the majority of respondents were nurses (n=34, 27%), physiotherapists (n=28, 23%) and doctors (n=22, 18%). There were also responses from occupational therapists (n=19, 15%), play therapists (n=7, 6%), psychologists (n=7, 6%), dieticians (n=6, 5%), and speech and language therapists (n=1, 1%). Almost three-quarters of health professionals had ≥5 years experience, with 48 (39%) ≥15 years. The majority of respondents considered ERM to be a priority, either crucial 15 (12%), very important 67 (55%) or important 35 (29%) in the care of PICU patients (Table 2).

Confidential: For Review Only

Table 2: Current views of ERM in PICU (n=121 respondents)

Current view of ERM in PICU	n (%)*
Crucial, should be the top priority in the care of PICU patients	15 (12)
Very important, should be a priority in the care of PICU patients	67 (55)
Important, should be a priority in the care of PICU patients	35 (29)
Somewhat important, should be considered in the care of PICU patients	4 (3)
Not of great importance, clinicians should bear it in mind in the care of PICU patients	0 (0)
Of minimal importance to the care of PICU patients	0 (0)
Of no importance to the care of the PICU patients	0 (0)

* Percentages may not total 100 due to rounding

Description of ERM

We invited respondents to describe ERM on their terms. Descriptions were provided by 104 (84%), which were summarised into four categories, 'Activity-focused', 'Tailored', 'Promote recovery', and 'Timing of ERM' (see Supplementary file 2, Table 1). Overall, ERM was considered to be an individualised package of graded interventions based on an activity-focused programme, to reduce the sequelae of critical illness or injury. However, responses differed for when ERM should be initiated, often emphasising the need for individualisation.

Availability of established ERM Protocols

Respondents were asked to describe the content of established ERM protocols within their PICU. Only 12 participants (10%) reported working in a PICU with an established ERM protocol (n=5/26, 19% of PICUs). The most common components of ERM protocols were 'physical therapy not requiring additional equipment' (n=9/12, 75%) and 'occupational therapy interventions' (n=8/12, 67%). Only 4/12 (33%) referred to play therapy or speech and language therapy, and no ERM protocol specified input from psychologists or psychiatrists. All participants were asked about the content of non-ERM protocols in their

https://mc.manuscriptcentral.com/bmjpo

Confidential: For Review Only

PICU. Only 18/124 (15%) reported that guidance for physical or occupational therapy activities existed in other non-ERM protocols within PICU (Table 3).

Table 3: Content of ERM and non-ERM protocols

Items	Within an ERM protocol (n=12 respondents) Yes n (%)	Within a non-ERM protocols (n=124 respondents) Yes n (%)
Physical therapy requiring additional equipment	9 (75)	18 (15)
Occupational therapy interventions	9 (75)	18 (15)
Physical therapy not requiring additional equipment	8 (67)	17 (14)
Speech and language therapy interventions	4 (33)	12 (10)
Psychology interventions	0 (0)	8 (6)
Delirium screening	0 (0)	1 (1)

Confidential: For Review Only

Recipients of ERM

51 (41%) respondents reported that *All PICU patients* 'always' or 'very often' received ERM (Supplementary file 2, Table 2). Overall, 14 (11%) respondents reported 'seldom' or 'never' delivering ERM.

ERM was reported to be more likely to be delivered to patients when PICU length of stay exceeded 28 days. Patients admitted for 28 days or more were more likely (n=91, 75%) to 'always' or 'very often' receive ERM compared to those admitted for shorter periods. Only 17 (13%) of those staying fewer than three days, 44 (36%) of those admitted between three to seven days and 73 (59%) of those admitted between seven to 28 days were more likely to receive ERM. . Participants reported that patients with acquired brain injury (n=75, 60%) and severe developmental delay (n=54, 44%) were 'always' or 'very often' likely to receive ERM.

Perceived benefits of ERM

Participants ranked the five most important potential benefits of ERM out of thirteen options (Figure 1 and Table 4). The most important outcomes identified were: 1) reduced PICU length of stay, 2) improved psychological outcomes for patients after PICU, 3) reduced days of mechanical ventilation, 4) improved participation in activities of daily living and 5) improved patient satisfaction.

Table 4: Top 5 most important benefits of Early Rehabilitation and Mobilisation

Perceived ERM benefits (Most to least important)	Sum Rank score*
Reduction in length of ICU stay	326
Improvement in the psychological impact of PICU care	288
Reduction in days requiring MV	242
Improvement in daily life participation following discharge	221
Improved patient satisfaction	158
Reduction in the rate of pulmonary complications	122
Reduction in patient delirium	95
Improved family satisfaction	94
Improvement in patient sleep quality	82
Reduction in treatment cost	60
Reduction in readmission	50
Increase in the number of patients discharged home	42
Improved staff satisfaction	27

*Sum of rank score: Ranking of top 5 (1-5) (1st placed rank scored 5 points to 5th placed scored 1 point). 121/124 (98%) participants ranked scores.

Initiation and delivery of ERM

The decision for ERM initiation was perceived by 96 (77%) to be primarily led by physiotherapists, doctors (n=92, 74%), and bedside nurses (n=64, 52%). Parents were felt to initiate ERM by only (n=24, 19%) of respondents (Table 5).

Confidential: For Review Only

Table 5: Which professional or parent groups in PICU initiates ERM (n=124 respondents)

Professional or family group	Yes n (%)
Physiotherapist	96 (77)
Physicians	92 (74)
Bedside nurse	64 (52)
Senior nurse	58 (47)
Other members of the medical team	55 (44)
Occupational therapist	37 (30)
Parent or family member	24 (19)

The most influential factor in ERM initiation was reported to be patient stability (n=69, 56%). Other influential factors were the length of stay; 15 (12%) reported ERM was initiated within 24 hours, and 16 (13%) within 2-3 days of PICU admission. Only 5 (4%) of respondents would not consider ERM at all on PICU. The influence of perceived clinical stability is demonstrated in respondents' free-text comments:

> "We are involved as early as required depending on the child/young person medical stability and their rehabilitation needs." (Occupational Therapist, 033)

> "Usually ERM activity is not considered until patients can physiologically tolerate movement and are cardiovascularly stable." (Nurse, 008)

Assessment of patient stability and tolerance of ERM was less well described. Most respondents (n=98, 79%) provided subjective cues or informal clinical criteria. These included monitoring vital signs, physiological changes, observing behavioural changes, and documenting adverse events.

https://mc.manuscriptcentral.com/bmjpo

Confidential: For Review Only

Physiotherapists (n=113, 92%), nurses (n=103, 84%), and parents or family members (n=92, 75%) were 'always' or 'very often' involved in the ongoing delivery of ERM with less frequent input from other members of the multi-disciplinary teams (Supplemental material file 2, Table 2).

Barriers to ERM Implementation

Figure 2 presents the perceived barriers of ERM (see also Supplemental material file 2, Table 3). The most significant barriers identified as institutional barriers were insufficient resources and equipment ('strongly agree' or 'agree': 83, 69%) and inadequate funding (73, 61%). Participants provided examples of resources having to be shared across organisations or specially ordered to deliver ERM to patients.

> "All equipment shared with the whole therapy department at present, therefore dependent on availability." (OT, 010)

A lack of established protocols (n=69, 57%), ERM champions (n=68, 57%), space (n=68, 56%), and robust patient screening processes (n=63, 58%) were also issues identified by respondents. Most PICUs had access to standard lifting 22/26 (85%) and specialist static seating equipment 25/26 (96%); however, bedside or in-bed cycling machines were only available in 10 (38%) of PICUs (see Table 6).

Limited staffing was the most frequently reported barrier to providers delivering ERM, with 101 (79%) 'agreeing' or 'strongly agreeing'. Approximately half of the respondents agreed that issues such as training, patient safety, lack of decision-making authority, and delays in recognising patients' ERM needs were barriers to ERM initiation. However, only 25 (21%)

Confidential: For Review Only

'agreed' or 'strongly agreed' that the impact of ERM potentially prolonging the working day was a barrier.

Table 6: Types of ERM equipment available in each Paediatric Intensive Care Unit (n=26)

ERM equipment available in each PICU	n (%)
Specialist static seating	25 (96)
Portable ventilators	23 (88)
Mobile lifts	22 (85)
Tilt table	22 (85)
Bed with full chair position	18 (69)
Specialist wheelchair	18 (69)
Bed with Trendelenburg features	13 (50)
Patient rolling walker	11 (42)
Bedside cycle or in-bed cycle	10 (38)
Ceiling lifts	8 (31)
Speciality bed with continuous side to side rotation	8 (31)
Bed with retractable footboard	7 (27)
Bed with chair egress exit out the foot of the bed	5 (19)
Transcutaneous Electrical Nerve stimulation	3 (12)

The two most frequently reported barriers to delivering ERM at the patient level were physiological instability (n=101, 81% agreeing or strongly agreeing) and sedation (n=91, 73%). Over 50% (n=64) 'disagreed' or 'strongly disagreed' that obesity was a barrier.

DISCUSSION

This national survey of health care practitioners from UK PICU's identified the importance of ERM as an intervention that participants believe can improve the physical and psychological recovery of critically ill or injured infants and children across all ages. Our findings indicate

Confidential: For Review Only

support for ERM but highlight uncertainty with suitability, variability with the definition of this complex intervention, variation in the timing of initiation and which patient groups should receive ERM. Key barriers to ERM delivery were identified (e.g. funding and staffing) and potential clinical (e.g. improved psychological outcomes) and economic (e.g. reduced PICU length of stay) benefits to patients and PICUs.

How early is 'early' in ERM has been challenging to define for health care professionals? A time-based definition (e.g. within the first 2-5 days of ICU admission) has been proposed[17]; however, this can conflict with the patient stability approach (e.g. start as early as the patient is able to receive it). Our results indicate uncertainty and wide variation in time to start ERM (24hrs to over seven days), increasing agreement for ERM to be considered after longer periods on PICU, and support for the concept of 'as early as the patient's clinical condition allows' which may be much longer. In the UK, only 43% of patients remain in PICU for three days or more[1]. The balance of delivering a programme of ERM to a large number of patients who may only receive intervention within a very short period versus targeting patients at higher risk of prolonged PICU stay needs to be considered. However, it has been proposed that for ERM to become embedded in clinical practice, it should become a standard of care within 48hrs of ICU admission[18].

The uncertainty of the content of ERM also adds to the challenge for health care professionals to appreciate when ERM could be delivered. Understandably, routine bedside nursing care (e.g. functional positioning) may be considered acceptable earlier than more advanced physical therapies requiring multiple staff (e.g. sitting a ventilated child out of bed or in-bed cycling). Our survey identified clinical stability as the most influential patient factor for initiation. The reported lack of ERM protocols in most (21/26) UK PICUs reinforces a

Confidential: For Review Only

strong requirement for evidence-based standardised protocols with optimal timing, intensity, frequency and duration of ERM. There is a need for flexible protocols to allow for tailoring rather than prescription.

ERM was more likely to be delivered to patients admitted for greater than 28 days among patients with acquired brain injury or severe developmental delay across all age ranges. This reflects the cross-over between ERM and established rehabilitation programmes following acquired brain injury[19] or for patients admitted to PICU with a pre-existing rehabilitation package. PICUs may be able to build on the success of established programmes to implement ERM to a wider critical care population and to use the existing multi-disciplinary expertise, and evolving rehabilitation evidence base to support the adoption of effective treatments. To date, most published ERM intervention studies have excluded patients less than three years of age[4 6]. However, this represents 60% of the UK PICU patient population[1], and this age group was as likely to receive ERM as older children in our study. Future ERM trials should include all PICU age groups to ensure ERM content and efficacy is assessed across all potential patients.

Our results show that doctors, physiotherapists, and nurses have an equally important role in the decision to initiate ERM within the UK NHS setting. This contrasts with other countries and health care settings where doctor-led approval was required by 70-81% of respondents, which was a potential barrier to mobilisation[20 21]. Nurses' and parents' roles are also important both in the initiation and delivery of ERM. In settings where nurses reported a low level (39%) of support for ERM, lack of involvement and understanding were key features[22]. In our study, 91% felt 'involved' in the delivery of ERM. However, health care professionals reported that parents were the least likely group to initiate ERM (19%),

Confidential: For Review Only

although becoming influential in its ongoing delivery. This is consistent with recent European[23] and North-American[11 24 25] point prevalence studies in PICUs, highlighting the important role parents play in delivering ERM. Building on both the multi-disciplinary support for ERM and empowering parents to initiate ERM may be potential strategies to improve implementation.

The key barriers to ERM practice were: i) at institutional level: insufficient resources, equipment and funding; ii) at provider level: limited staffing, training, protocols and slow recognition of readiness for ERM; and iii) at patient-level: physiological instability**,** risk of endotracheal tube dislodgement and amount of sedation. These barriers have been previously described in other health settings[20-22 26] with proposed facilitators, including adopting formal protocol/manual[27] (a key feature lacking across most UK PICUs), local champions[27] and team engagement / collaboration[27 28]. Implementation of ERM within the NHS will require building on the established multi-disciplinary teams, incorporating ERM within established protocols for weaning off mechanical ventilation and sedation and realistic goal setting. The potential benefits of ERM in terms of PICU length of stay and improvement in the psychological outcome for patients should be assessed within the core outcomes evaluating the efficacy of ERM within PICU.

The strength of this survey was an inclusive representation of 90% of UK PICUs and views from the wider multi-disciplinary team. However, none or partial responses may indicate poor engagement in ERM topic, and as with all self-reported surveys, responses indicate reported rather than actual clinical practice. A limitation was the use of a non-validated questionnaire. We did not conduct questionnaire validation because it would be very difficult to standardise these questions within the time constraints of the study and the

Confidential: For Review Only

NIHR HTA funding. The study team considered the questionnaire suitable for the study aims after receiving two rounds of expert reviews. Finally, the findings represent the views of UK NHS staff and may not be generalisable to other healthcare settings.

CONCLUSION

ERM, in some form, is currently delivered to critically ill or injured patients of all ages across UK PICUs, but significant barriers to full implementation exist due to resource limitations and lack of institutional and national guidance. The UK should build on the existing strong multi-disciplinary support for ERM in PICUs. A standardised protocol that sets out defined ERM interventions and implementation support to tackle modifiable barriers is required to ensure the delivery of high-quality ERM.

Confidential: For Review Only

Acknowledgements This study/project is funded by the National Institute for Health Research (NIHR), project reference: 17/21/ HTA (Health Technology Assessment). The views expressed are those of the author(s) and not necessarily those of the NIHR or the Department of Health and Social Care.

We thank the Paediatric Intensive Care Society Study Group (PICS-SG), all members of the Paediatric Intensive Care Units and Principal Investigators for their contribution to the development of the PERMIT survey.

- Addenbrooke's Cambridge, Dr Nazima Pathan
- Alder Hey Children's NHS Foundation Trust, Liverpool, Dr Petr Jirasek
- Birmingham Women & Children's Hospital NHS Foundation Trust, Dr Barnaby Scholefield
- Bristol Royal Hospital for Children, Kate Baptiste
- Evelina London Children's Hospital, Dr Miriam Fine-Goulden
- Freeman Hospital, Newcastle upon Tyne Hospitals NHS Foundation Trust, Dr Deborah Cross
- GOSH CICU, Great Ormond Street Hospital for Children NHS Foundation Trust, Emma Shkurka
- GOSH PICU, Great Ormond Street Hospital for Children NHS Foundation Trust, Dr Peter Sidgwick
- Great North Children's Hospital, Newcastle, Dr Rachel Agbeko
- Kings College Hospital NHS Foundation Trust, Dr Bogdana Zoica
- Leeds Teaching Hospitals NHS Trust, St. James's University Hospital, Dr Sian Cooper
- Noah's Ark Children's Hospital for Wales, Dr Siva Oruganti
- Nottingham Children's Hospital, Queen's Medical Centre, Dr Georgina Harlow
- Oxford University Hospitals, Dr Deirdre O'Shea
- Royal Belfast Hospital for sick children, located in Royal Victoria Hospital, Dr Julie Richardson
- Royal Hospital for Children Glasgow, Dr Richard Levin
- Royal Hospital for Sick Children in Edinburgh, Dr Tsz Yan and Dr Milly Lo
- Royal Manchester Children's Hospital, Dr Ravishankar Nagaraj
- Sheffield Children's Hospital NHS Foundation Trust, Dr Rum Thomas
- Southampton Children's Hospital, Katy Morton
- St Georges Hospital, London, Dr Soumendu Manna
- St Mary's Hospital, Praed Street, Imperial London, Dr Tom Bycroft
- University Hospital Leicester, Dr Sanjiv Nichani
- Glenfield Hospital, Dr Sanjiv Nichani
- University Hospital North Midlands, Dr Kanaris Constantinos
- South Tees Hospitals NHS Foundation Trust

Contributions

BRS, JCMen, JCMan, RF, TR, NP, SB, JM, DM, MG, GC, KM, RP, RF, SL, and FK developed the original study protocol. BRS, EB, JCMan and FR developed the pilot survey and preliminary analysis. JT, JCMen, JCMan and BRS developed the final survey, coordinated administration, interpretation and analysis of data and BRS and JT drafted the manuscript, which has had input from all authors. All authors revised and approved the final manuscript. Paediatric

Confidential: For Review Only

Intensive Care Society Study-Group (PICS-SG) members reviewed the protocol and endorsed the survey.

Competing interests: Dr Julie C. Menzies is funded as a National Institute for Health Research (NIHR) 70@70 Senior Nurse and Midwife Research Leader. Dr Joseph C. Manning is an NIHR Health Education England funded ICA Clinical Lecturer. Dr Barnaby Scholefield receives funding as an NIHR Clinician Scientist Fellow, and Dr Forsyth and Prof Rapley receives NIHR grant funding. The views expressed in this article are those of the authors and not necessarily those of the NIHR, or the Department of Health and Social Care.

Funding informtion: This study/project is funded by the National Institute for Health Research (NIHR) [Health Technology Assessment Programme (project reference: 17/21)/ HTA]. The views expressed are those of the author(s) and not necessarily those of the NIHR or the Department of Health and Social Care.

Patient consent for publication: Not required.

Ethics approval: The study received ethical approval granted by the University of Birmingham review panel on 05/02/2019

Confidential: For Review Only

What is already known?

- Early rehabilitation and mobilisation (ERM) interventions are safe, feasible and effective within adult intensive care, but the evidence base in a paediatric setting is limited.
- In critically ill adults, ERM delivery is tailored according to the patient's cardiovascular support requirements, levels of consciousness and tolerance levels.
- Barriers to implementing ERM in paediatric intensive care units (PICUs) have been described in North America, but little is known about these within the UK NHS setting.

What does this study add?

- Despite positivity towards the concept of ERM, less than 20% of UK PICUs currently have an established ERM protocol to define ERM content and practice.
- ERM initiation and delivery is collaborative, but there is wide variability on which patients can receive ERM and when this should be initiated.
- The provision of a standardised protocol that sets out safe and defined ERM activities along with implementation support would tackle modifiable barriers of intervention delivery
- The most important barriers to ERM delivery in PICUs within the NHS are financial resources and staffing, lack of protocols for patient selection and ERM guidelines.

Confidential: For Review Only

References

1. PICANet. Paediatric Intensive Care Audit Network National Annual Report 2016 - 2018. 2019. https://www.picanet.org.uk/annual-reporting-and-publications/ (accessed 15th September 2020).
2. Lopes-Junior LC, Rosa M, Lima RAG. Psychological and Psychiatric Outcomes Following PICU Admission: A Systematic Review of Cohort Studies. *Pediatr Crit Care Med* 2018;19(1):e58-e67. doi: 10.1097/pcc.0000000000001390 [published Online First: 2017/12/01]
3. Manning JC, Pinto NP, Rennick JE, et al. Conceptualizing Post Intensive Care Syndrome in Children-The PICS-p Framework. *Pediatr Crit Care Med* 2018;19(4):298-300. doi: 10.1097/pcc.0000000000001476 [published Online First: 2018/02/07]
4. Choong K, Awladthani S, Khawaji A, et al. Early Exercise in Critically Ill Youth and Children, a Preliminary Evaluation: The wEECYCLE Pilot Trial. *Pediatr Crit Care Med* 2017;18(11):e546-e54. doi: 10.1097/pcc.0000000000001329 [published Online First: 2017/09/19]
5. Pun BT, Balas MC, Barnes-Daly MA, et al. Caring for Critically Ill Patients with the ABCDEF Bundle: Results of the ICU Liberation Collaborative in Over 15,000 Adults. *Crit Care Med* 2019;47(1):3-14. doi: 10.1097/ccm.0000000000003482 [published Online First: 2018/10/20]
6. Fink EL, Beers SR, Houtrow AJ, et al. Early Protocolized Versus Usual Care Rehabilitation for Pediatric Neurocritical Care Patients: A Randomized Controlled Trial. *Pediatr Crit Care Med* 2019 doi: 10.1097/pcc.0000000000001881. [published Online First: 2019/02/02]
7. Nydahl P, Sricharoenchai T, Chandra S, et al. Safety of Patient Mobilization and Rehabilitation in the Intensive Care Unit. Systematic Review with Meta-Analysis. *Ann Am Thorac Soc* 2017;14(5):766-77. doi: 10.1513/AnnalsATS.201611-843SR. [published Online First: 2017/02/24]
8. National Institute for Health and Care Excellence. Rehabilitation after critical illness in adults [NICE guidance CG83]. 2009 25 March 2009. https://www.nice.org.uk/guidance/CG83 (accessed 15th September 2020).
9. Wieczorek B, Burke C, Al-Harbi A, et al. Early mobilization in the pediatric intensive care unit: a systematic review. *J Pediatr Intensive Care* 2015;2015:129-70. doi: 10.1055/s-0035-1563386 [published Online First: 2015/09/18]
10. Cuello-Garcia CA, Mai SHC, Simpson R, et al. Early Mobilization in Critically Ill Children: A Systematic Review. *The Journal of pediatrics* 2018;203:25-33.e6. doi: 10.1016/j.jpeds.2018.07.037 [published Online First: 2018/09/03]
11. Choong K, Zorko D, Awojoodu R, et al. Prevalence of acute rehabilitation for kids in the picu: A canadian multicenter point prevalence study (park-picu canada). *Pediatr Crit Care Med* 2021;22:68-69.
12. Ista E, Redivo J, Kananur P, et al. ABCDEF Bundle Practices for Critically Ill Children: An International Survey of 161 PICUs in 18 Countries. *Crit Care Med* 2022;50(1):114-25. doi: 10.1097/CCM.0000000000005168.
13. Mayring P. Qualitative Content Analysis. *2000* 2000;1(2) doi: 10.17169/fqs-1.2.1089 [published Online First: 2000-06-30]
14. Bengtsson M. How to plan and perform a qualitative study using content analysis. *NursingPlus Open* 2016;2:8-14. doi: https://doi.org/10.1016/j.npls.2016.01.001
15. Vaismoradi M, Turunen H, Bondas T. Content analysis and thematic analysis: Implications for conducting a qualitative descriptive study. *Nurs Health Sci* 2013;15(3):398-405. doi: 10.1111/nhs.12048. Epub 2013 Mar 11. [published Online First: 2013/03/14]
16. Burnard P. A method of analysing interview transcripts in qualitative research. *Nurse Education Today* 1991;11(6):461-66. doi: https://doi.org/10.1016/0260-6917(91)90009-Y
17. Hodgson CL, Berney S, Harrold M, et al. Clinical review: Early patient mobilization in the ICU. *Critical Care* 2013;17(1):207. doi: 10.1186/cc11820

Confidential: For Review Only

18. Cameron S, Ball I, Cepinskas G, et al. Early mobilization in the critical care unit: A review of adult and pediatric literature. *J Crit Care* 2015;30(4):664-72. doi: 10.1016/j.jcrc.2015.03.032 [published Online First: 2015/05/20]
19. Hayes L, Shaw S, Pearce MS, et al. Requirements for and current provision of rehabilitation services for children after severe acquired brain injury in the UK: a population-based study. *Archives of Disease in Childhood* 2017;102(9):813. doi: 10.1136/archdischild-2016-312166
20. Anekwe DE, Koo KK, de Marchie M, et al. Interprofessional Survey of Perceived Barriers and Facilitators to Early Mobilization of Critically Ill Patients in Montreal, Canada. *J Intensive Care Med* 2017:885066617696846. doi: 10.1177/0885066617696846. [published Online First: 2017/03/31]
21. Choong K, Koo KK, Clark H, et al. Early mobilization in critically ill children: a survey of Canadian practice. *Critical care medicine* 2013;41(7):1745-53. doi: 10.1097/CCM.0b013e318287f592 [published Online First: 2013/03/20]
22. Wang J, Xiao Q, Zhang C, et al. Intensive care unit nurses' knowledge, attitudes, and perceived barriers regarding early mobilization of patients. *Nurs Crit Care* 2020 doi: 10.1111/nicc.12507. [published Online First: 2020/04/15]
23. Ista E, Scholefield BR, Manning JC, et al. Mobilization practices in critically ill children: a European point prevalence study (EU PARK-PICU). *Critical Care* 2020;24(1) doi: 10.1186/s13054-020-02988-2
24. Curry A, Brown A, Williams S, et al. Impact of an early mobility program in the hematologic oncologic PICU setting. *Crit Care Med* 2021;49(1):527.
25. Nelliot A, Awojoodu R, Walker T, et al. Physical Rehabilitation in Critically Ill Children: A Multicenter Point Prevalence Study in the United States. *Crit Care Med* 2020:634-44.
26. Fontela PC, Forgiarini LA, Jr., Friedman G. Clinical attitudes and perceived barriers to early mobilization of critically ill patients in adult intensive care units. *Rev Bras Ter Intensiva* 2018;30(2):187-94. doi: 10.5935/0103-507x.20180037 [published Online First: 2018/07/12]
27. Goddard SL, Lorencatto F, Koo E, et al. Barriers and facilitators to early rehabilitation in mechanically ventilated patients-a theory-driven interview study. *J Intensive Care* 2018;6:4. doi: 10.1186/s40560-018-0273-0. eCollection 2018. [published Online First: 2018/02/07]
28. Zheng K, Sarti A, Boles S, et al. Impressions of Early Mobilization of Critically Ill Children-Clinician, Patient, and Family Perspectives. *Pediatr Crit Care Med* 2018;19(7):e350-e57. doi: 10.1097/PCC.0000000000001547 [published Online First: 2018/04/13]

Confidential: For Review Only

Figure 1: Perceived Benefits of Early Rehabilitation and Mobilisation
Ranking of participants potential top 5 perceived benefits of delivering ERM within PICU's. Sum of rank score: Ranking of top 5 (1-5) (1st placed rank scored 5 points to 5th placed scored 1 point). 121/124 (98%) participants ranked scores.

Figure 2: Perceived Barriers of Early Rehabilitation and Mobilisation
Institutional, Patients and Provider barriers to ERM. Figure 2 shows the percentage of responses for categories strongly agree, agree, neutral, disagree and strongly disagree. Responses ranked on the cumulative score of percentage 'strongly agree and agree'.

Confidential: For Review Only

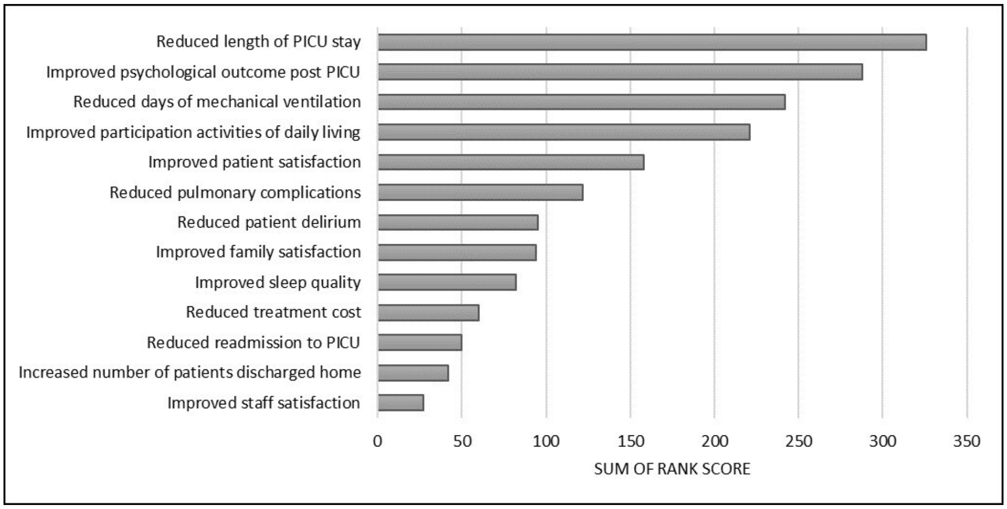

88x44mm (300 x 300 DPI)

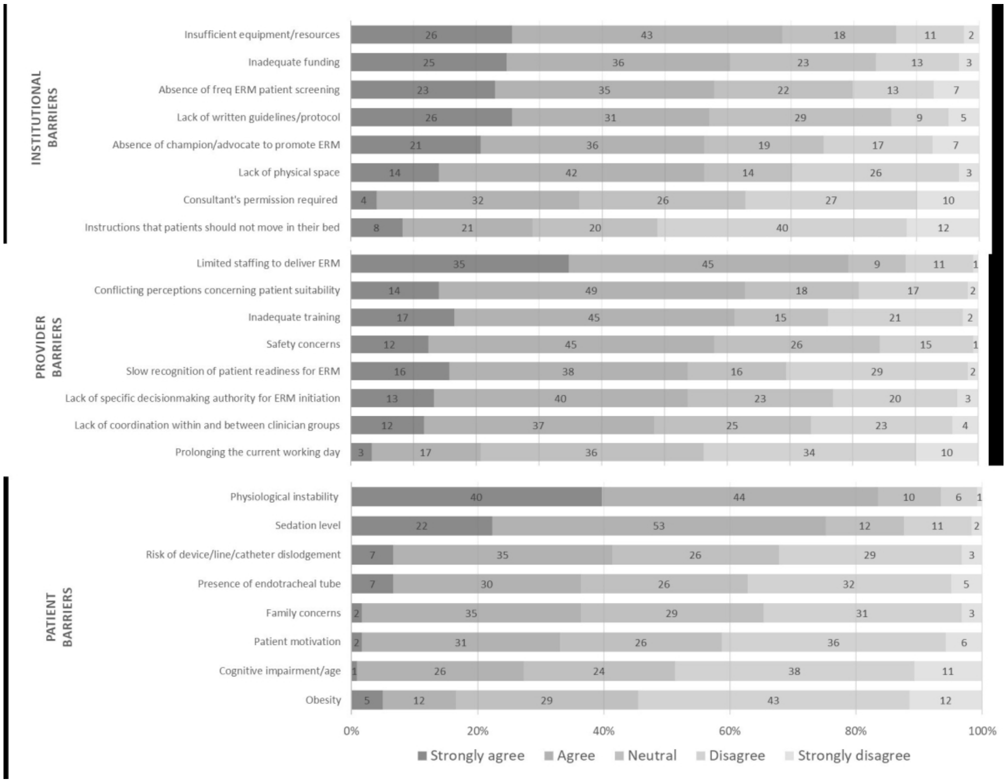

153x119mm (300 x 300 DPI)

Supplementary Material File 1

PERMIT Online Survey

Paediatric Early Rehabilitation and Mobilisation during InTensive care (PERMIT) Survey

1. Introduction

Thank you for your interest in completing a questionnaire as part of the PERMIT Study.

Why have I been invited to take part?
You have been invited to take part because you are a health care practitioner responsible for the delivery of care to children in a PICU. We would like the views of a wide range of health care professionals, and you have been identified either through the collaborating Paediatric Intensive Care Society, Physiotherapy and Occupational therapy membership lists or recommended by lead clinicians in your institutions.

Study Overview
We are conducting research into Early Rehabilitation and Mobilisation (ERM) during paediatric critical illness in the UK and assessing the feasibility and design of a future clinical trial. ERM can encompass patient-tailored interventions or bundles of care provided within the critical care unit. Rehabilitation has been shown to improve quality of life and patient outcomes in the adult ICU; however, there is limited evidence in the PIC patient population and setting.

The whole PERMIT programme aims to assess key questions regarding the acceptability and feasibility of ERM in PIC and form recommendations for a future clinical trial to establish its effectiveness.

In this questionnaire, we would like to find out:
- What forms of ERM are provided in your PICs and to which patients;
- What resources are available (or could be available) to deliver ERM;
- Your views on evaluating ERM interventions;
- Whether you would be interested in a future clinical trial to assess ERM.

Thank you for your time (which we calculated should be less than 20 minutes) in completing this questionnaire.

Barney Scholefield & Jacqueline Thompson

Confidential: For Review Only

On behalf of the PERMIT study team and the Paediatric Intensive Care Society -Study Group (PICS-SG)

2. A little demographic information about you please

1. Which hospital do you work at? *

Other (if not listed)

2. Which best reflects your current professional position? *

- [ ] Medical
- [ ] Nurse
- [ ] Physiotherapist
- [ ] Occupational therapist
- [ ] Speech and Language therapist
- [ ] Dietician
- [ ] Play therapist
- [ ] Psychologist
- [ ] Other (please specify):

3. Number of years experience working in PICU? *

- [ ] <1 year
- [ ] 1 to <5 years
- [ ] 5 to <10 years
- [ ] 10 to <15 years
- [ ] 15 to <20 years

Confidential: For Review Only

- [ ] More than 20 years
- [ ] I do not work in PICU

Comments:

3. What ERM do you currently provide in your PICU?

In the following questions, we hope to explore your current practice related to early rehabilitation and mobilisation (ERM). We understand that the term ERM may mean different things to different professional groups; however, we are keen to capture the current diverse views and how ERM is defined by you and your PICU team.

4. Please describe in the box below the current ERM practice within your PICU. (We are especially interested in whether you provide anything that you feel fits within the term ERM, who receives ERM, what specific actions are involved in ERM and who delivers the ERM).

5. Which of the following best describes when you would consider ERM for a patient? *

- [ ] Within 24 hours of admission to PICU
- [ ] About 2 to 3 days after PICU admission
- [ ] Around the end of the first week of PICU admission
- [ ] Before discharge from PICU
- [ ] As soon as the patient is stable enough
- [ ] As soon as the patient is off mechanical ventilatory support
- [ ] To start earlier than we historically considered starting rehabilitation and mobilisation
- [ ] We do not consider ERM for our PICU patients
- [ ] I don't know
- [ ] Other (please specify):

Confidential: For Review Only

Comments:

6. Which team members are involved in ERM in your PICU? (Please select how frequently they are involved. If the team member is not available in your PICU please select 'Not applicable')

	Always	Very often	Sometimes	Seldom	Never	Not applicable (as team member is not available in my PICU)	Don't know
Medical staff	☐	☐	☐	☐	☐	☐	☐
Nursing staff	☐	☐	☐	☐	☐	☐	☐
Speech and language therapist	☐	☐	☐	☐	☐	☐	☐
Physiotherapist	☐	☐	☐	☐	☐	☐	☐
Occupational therapist	☐	☐	☐	☐	☐	☐	☐
Psychologist	☐	☐	☐	☐	☐	☐	☐
Play therapist	☐	☐	☐	☐	☐	☐	☐
Dietician	☐	☐	☐	☐	☐	☐	☐
Patients family or carers	☐	☐	☐	☐	☐	☐	☐
Other	☐	☐	☐	☐	☐	☐	☐

Please define 'other' or add any comments

Confidential: For Review Only

Confidential: For Review Only

7. How often do patients, in general, receive ERM in your PICU?

	Always	Very often	Sometimes	Seldom	Never	Don't know
All PICU patients	☐	☐	☐	☐	☐	☐

Comments:

8. How often do patients in the following age groups receive ERM in your PICU? (Please select an option for each)

	Always	Very often	Sometimes	Seldom	Never	Don't know
Infants & <1 year old	☐	☐	☐	☐	☐	☐
1-4 years old	☐	☐	☐	☐	☐	☐
> 4-10 years old	☐	☐	☐	☐	☐	☐
> 10-18 years old	☐	☐	☐	☐	☐	☐

Comments:

9. How often do patients within the following diagnostic categories receive ERM in your PICU? (Please select an option for each)

	Always	Very often	Sometimes	Seldom	Never	Don't know
Acquired brain injury patients (e.g. TBI, meningitis)	☐	☐	☐	☐	☐	☐
Sepsis	☐	☐	☐	☐	☐	☐
Congenital heart disease	☐	☐	☐	☐	☐	☐
Cancer	☐	☐	☐	☐	☐	☐
Mechanically ventilated	☐	☐	☐	☐	☐	☐

	Always	Very often	Sometimes	Seldom	Never	Don't know
Mechanically supported (e.g. ECMO)	☐	☐	☐	☐	☐	☐
Respiratory illness (bronchiolitis or pneumonia)	☐	☐	☐	☐	☐	☐
Multi-organ failure	☐	☐	☐	☐	☐	☐
With pre-existing physical co-morbidity	☐	☐	☐	☐	☐	☐
With pre-existing severe developmental delay	☐	☐	☐	☐	☐	☐

Comments:

10. How often do patients, with the following length of stay, receive ERM in your PICU? (Please select an option for each)

	Always	Very often	Sometimes	Seldom	Never	Don't know
Patients in PICU <3 days	☐	☐	☐	☐	☐	☐
Patients in PICU 3-7 days	☐	☐	☐	☐	☐	☐
Patients in PICU >7-28 days	☐	☐	☐	☐	☐	☐
Patients in PICU >28 days	☐	☐	☐	☐	☐	☐

Comments:

11. Who decides if the patient can start to receive ERM? (Please select all that apply)

☐ Medical consultant

☐ Other member of the medical team (e.g. PICU trainee/ advanced nurse practitioner)

https://mc.manuscriptcentral.com/bmjpo

Confidential: For Review Only

- [ ] Bedside nurse
- [ ] Senior nurse on shift
- [ ] Physiotherapist
- [ ] Occupational therapist
- [ ] Parent(s)
- [ ] Don't know
- [ ] Not applicable
- [ ] Other (please specify):

Comments:

12. How does your PICU assess whether patients tolerate and cope with the therapy provided? (eg monitor safety)

13. Do you currently use or have an established guideline or protocol regarding management of ERM in your PICU?

- [ ] No
- [ ] Yes - if you are able to share, please email to b.scholefield@bham.ac.uk
- [ ] Don't know

Comments:

4. Question regarding your established ERM programme or protocol

14. What do you have within your protocolised ERM treatment? (Please select all that apply)

Confidential: For Review Only

- [ ] Physical therapy not requiring additional equipment
- [ ] Physical therapy requiring additional equipment
- [ ] Occupational therapy
- [ ] Speech and language therapy
- [ ] Psychology
- [ ] Delirium screening
- [ ] We do not have a programme or protocol related to ERM activities
- [ ] Other (please specify):

Comments:

15. What are the key factors in successfully implementing your ERM protocol?

5. ERM related processes and equipment in your PICU

The following three questions are related to additional processes or equipment in your PICU related to ERM activities.

16. Does your PICU have a protocol or guidelines for the management of the following?

	Yes	No	Don't know
Sedation	☐	☐	☐
Analgesia	☐	☐	☐
Drug/ opioid withdrawal	☐	☐	☐
Delirium	☐	☐	☐

Confidential: For Review Only

	Yes	No	Don't know
Mobilisation (at any time point)	☐	☐	☐

17. What types of therapies or activities are available in your PICU? (Please select all that apply)

- ☐ Physical therapy - not requiring additional equipment (e.g. passive or active movements)
- ☐ Physical therapy - requiring additional equipment (e.g. in bed cycling)
- ☐ Occupational therapy
- ☐ Speech and language therapy
- ☐ Clinical psychology
- ☐ Delirium screening
- ☐ Patient diaries
- ☐ Play therapy
- ☐ Pet therapy
- ☐ Music therapy
- ☐ Other (please specify):

18. What equipment does your PICU have available for physical therapy/mobilisation? (Please select all that apply)

- ☐ Tilt table
- ☐ Bed with Trendelenburg features
- ☐ Bed with full chair position
- ☐ Bed with chair egress - exit out the foot of the bed
- ☐ Speciality bed with continuous side to side rotation
- ☐ Bed with retractable footboard i.e. flex a foot feature

Confidential: For Review Only

- [ ] Mobile lifts
- [ ] Ceiling lifts
- [ ] Portable ventilators
- [ ] Patient rolling walker
- [ ] Bedside cycle (patient can remain in bed)
- [ ] Transcutaneous electrical stimulation of muscles
- [ ] Specialist wheelchair
- [ ] Specialist static seating i.e. tumble forms
- [ ] Other (please specify):

6. Preferences, barriers and outcomes

For the following questions, we would explore your strength of opinion on ERM as a therapy, potential barriers to using ERM in practice and which are the most important clinical outcomes to consider when assessing if ERM is beneficial.

19. Please select one option regarding your current views of ERM in your PICU? *

- [ ] Crucial, should be the top priority in the care of PICU patients
- [ ] Very important, should be a priority in the care of PICU patients
- [ ] Important, should be a priority in the care of PICU patients
- [ ] Somewhat important, should be considered in the care of PICU patients
- [ ] Not of great importance, clinicians should bear it in mind in the care of PICU patients
- [ ] Of minimal importance to the care of PICU patients
- [ ] Of no importance to the care of PICU patients

Comments:

Confidential: For Review Only

20. Below are potential institutional barriers to the delivery of ERM. Please select to what extent you agree/disagree that each are barriers to delivery of ERM in your PICU. *

	Strongly Agree	Agree	Neutral	Disagree	Strongly Disagree
Instructions that patients should not move in their bed	☐	☐	☐	☐	☐
Consultant's permission or prescription is required prior to mobilisation	☐	☐	☐	☐	☐
Insufficient equipment/ resources	☐	☐	☐	☐	☐
Inadequate funding	☐	☐	☐	☐	☐
Lack of written guidelines/ protocol	☐	☐	☐	☐	☐
Lack of physical space	☐	☐	☐	☐	☐
Absence of champion/ advocate to promote ERM	☐	☐	☐	☐	☐
Absence of frequent (e.g. daily) ERM patient screening	☐	☐	☐	☐	☐

Comments:

21. Below are potential patient barriers to the delivery of ERM. Please select to what extent you agree/disagree that each are barriers to delivery of ERM in your PICU. *

	Strongly Agree	Agree	Neutral	Disagree	Strongly Disagree
Physiological instability ('the patient is just too sick')	☐	☐	☐	☐	☐

Confidential: For Review Only

	Strongly Agree	Agree	Neutral	Disagree	Strongly Disagree
Sedation level	☐	☐	☐	☐	☐
Presence of endotracheal intubation	☐	☐	☐	☐	☐
Risk of device/ line/ catheter dislodgement	☐	☐	☐	☐	☐
Cognitive impairment/ age	☐	☐	☐	☐	☐
Obesity	☐	☐	☐	☐	☐
Patient motivation	☐	☐	☐	☐	☐
Family concerns	☐	☐	☐	☐	☐

Comments:

22. Below are potential provider barriers to the delivery of ERM. Please select to what extent you agree/disagree that each are barriers to delivery of ERM in your PICU. *

	Strongly Agree	Agree	Neutral	Disagree	Strongly Disagree
Lack of prioritisation of ERM within patient care plans	☐	☐	☐	☐	☐
Limited staffing to deliver ERM	☐	☐	☐	☐	☐
Lack of coordination within and between clinician groups	☐	☐	☐	☐	☐
Slow recognition of patient readiness for ERM	☐	☐	☐	☐	☐
Conflicting perceptions concerning patient suitability	☐	☐	☐	☐	☐

Confidential: For Review Only

	Strongly Agree	Agree	Neutral	Disagree	Strongly Disagree
Safety concerns	☐	☐	☐	☐	☐
Lack of specific decision-making authority for ERM initiation	☐	☐	☐	☐	☐
Inadequate training	☐	☐	☐	☐	☐
Prolonging the current working day	☐	☐	☐	☐	☐

Comments:

23. Please rank what you perceive to be the top 5 most important benefits that ERM could provide in PICU. (Please select, in order, the top perceived benefits by selecting a box). *

	1 (most important)	2	3	4	5 (least important)
Reduction in treatment cost	☐	☐	☐	☐	☐
Reduction in patient delirium	☐	☐	☐	☐	☐
Reduction in length of ICU stay	☐	☐	☐	☐	☐
Reduction in days requiring mechanical ventilation	☐	☐	☐	☐	☐
Reduction in the rate of pulmonary complications	☐	☐	☐	☐	☐
Reduction in readmission (hospital and PICU)	☐	☐	☐	☐	☐
Increase in number of patients discharged home	☐	☐	☐	☐	☐
Improved patient satisfaction	☐	☐	☐	☐	☐

Confidential: For Review Only

	1 (most important)	2	3	4	5 (least important)
Improved staff satisfaction	☐	☐	☐	☐	☐
Improved family satisfaction	☐	☐	☐	☐	☐
Improvement in patient sleep quality	☐	☐	☐	☐	☐
Improvement in psychological impact of PICU care	☐	☐	☐	☐	☐
Improvement in daily life participation following discharge	☐	☐	☐	☐	☐

Comments:

7. Final thoughts

24. Would you be willing to support a future clinical trial in the UK and Ireland based on evaluating ERM practice?

☐ Yes

☐ No

☐ Don't Know

Comments:

25. Would you allow us to contact you in the future with information regarding workshops we will be running to discuss ERM practice and trial feasibility, please? *

☐ Yes (please click the link on the next page to provide best contact information, thank you!)

☐ No

Confidential: For Review Only

Funding statement: This study/project is funded by the National Institute for Health Research (NIHR) [Health Technology Assessment Programme (project reference: 17/21)/ HTA]. The views expressed are those of the author(s) and not necessarily those of the NIHR or the Department of Health and Social Care.

Confidential: For Review Only

Supplementary Material File 2

Table 1: Descriptions of ERM

Category	Sub-categories	Examples:
**Activity-focused**<br>ERM is activity-focused, with consideration of seating and equipment, primarily delivered by nursing and allied health care professionals with optimised sedation management to promote patient engagement. There is a lack of consensus on what is routine care compared with purposeful ERM activity.	Activity – mobilising, positioning, stretching	"ERM provided includes - passive movements of limbs, periodic change of position, use of splints on extremities and if stable in the long term patients - sitting up in tumble form chair and mobilising out of bed." (Doctor, 67)<br><br>"We aim to get the children sitting upright, either over the edge of the bed or inappropriate seating as soon as possible." (Occupational Therapist, 001)<br><br>"Nursing staff are taught the importance of regular position changes for patient ... and positioning to maintain ranges of movement and prevent foot drop." (Nurse, 017)
	Core health care professional involvement – physiotherapist, nurse, occupational therapist	"We try and mobilise patients as soon as able when not invasively ventilated - physio led." (Doctor, 058)<br><br>"As physiotherapists, we work with the occupational therapist and nursing staff to either re-position, sit up in bed, sit on the edge of the bed or stand whilst on the ventilators."(Physiotherapist, 069)
	Additional health care professional involvement – psychology, dietician, play therapist, speech and language therapists	"Speech and language therapy become involved usually when nursing or medical staff identify a need for referral." (Doctor, 003)<br><br>"We have a Play Specialist on PICU, who assists with communication tools/toys." (Nurse, 082)
	Seating and equipment	"Specialist seating need – linking in with OT to ensure appropriate seating available." (Physiotherapist, 019)

Confidential: For Review Only

		"We have also in the past asked adult [services] to use their moto-med bike and used this with teenagers, but this can be a challenge as it is very far from PICU and it is often in use." (Physiotherapist, 014)
	Sedation management	"Working with the medics to wean sedation as quickly as possible. This promotes faster ability to mobilise and progress in their rehabilitation." (Nurse, 034)
	'Routine care' vs 'purposeful ERM package'	Some centres define ERM as passive range of movement, repositioning or providing splints. We would deem this as essential core cares rather than ERM; the large majority of our patients will have positioning and movement plan from day 1 of admission." (Physiotherapist, 111)
**Tailored** Following the assessment of needs and patient and family preference, ERM activities are personalised to the individual patient.	Assessment of need	"We have a programme where patients are categorised to one of 3 levels. Each level provides nursing and therapy staff with activities aligned to the acuity of the patient." (Nurse, 040)
	Individual preference	"Parents will tell us what their child enjoys doing and make suggestions, often provide toys/games from home." (Nurse, 076)<br><br>"Discuss with parents what patient enjoys doing, watching etc. Discuss options regarding taking patients 'out' where possible if long term patients." (Nurse, 015)
**Promote recovery** The purpose of ERM is to normalise the PICU environment, to create an environment that addresses holistic needs, sustains or promotes development and supports recovery from critical illness.	Normalising PICU environment	"Provide activities they would use for their enjoyment. This enables them to be more relaxed and less aware of what is going on with / around them." (Play specialist, 102)<br><br>"Being in PICU can be a frightening experience. Not only is a child/young person away from home, but also away from their usual environment, family and friends. There are unfamiliar sounds, smells, equipment and people. The play specialist can help to make the stay much more enjoyable and children/young people to cope and understand." (Play specialist, 055)
	Sustain / promote child development	"Encouraging the patient to regain or further their development in a therapeutic fun manner." (Play therapist,102)

Confidential: For Review Only

	Restoration / recovery	"Interventions aim to promote physical recovery – movement, and ability to engage in activities and psychological recovery – orientation, speech, ability to play and attend school. " (Nurse, 021)
**Timing of ERM**<br>The optimal timing of ERM initiation is challenging for health care professionals to define, although likelihood perceived to increase with increased length of stay. Influenced by the perceived stability of patients and the balance of risk to benefit.	'Early' poorly defined	"I like to think we consider it within 2-3 days we have a better idea of the patients PICU journey and how long they are going to stay. The reality is it's usually longer... usually week 2 of admission if they are still on the unit." (Physiotherapist, 090)<br><br>"There is no formal plan for who decides if a patient should start ERM and when… Currently the delivery of ERM is fairly ad hoc, based on what is highlighted from daily handover each morning." (Physiotherapist, 083)<br><br>"We provide ERM as a structured programme… every patient after 24 hours of admission is considered for ERM." (Doctor, 012)
	Patient stability, risk and benefit	"Members of the PICU team with less experience of early rehab can deem ERM 'unsafe' which can occasionally act as a barrier."(Physiotherapist, 016)<br><br>"Usually ERM activity is not considered until patients can physiologically tolerate movement and are stable with observations." (Nurse, 008)
	'Long-stay'	"ERM is often thought about as a patient comes up to extubation or has failed extubation, and we feel that the reason for failure may be because of critical care weakness." (Physiotherapist, 031)<br><br>"We provide very little ERM on PICU, other than for long term patients, which is often, in my opinion, delayed." (Nurse, 007)

Confidential: For Review Only

Table 2: Reported frequency of receiving or being involved in Early Rehabilitation and Mobilisation by age groups, length of PICU stay, diagnostic category, and health care professional or parental/family role (n=124 respondents)*

By age group	Always (n, %)	Very often (n, %)	Sometimes (n, %)	Seldom (n, %)	Never (n, %)	Don't know (n, %)	Not Applicable[#] n
All PICU Patients (any age)	6 (5)	45 (36)	48 (39)	14 (11)	0 (0)	11 (9)	0
>10-18 years old	18 (15)	40 (32)	43 (35)	8 (6)	1 (1)	14 (11)	0
>4-10 years old	16 (13)	42 (34)	41 (33)	10 (8)	1 (1)	14 (11)	0
1-4 years old	13 (10)	42 (34)	39 (31)	13 (10)	1 (1)	16 (13)	0
Infants & <1 year old	6 (5)	47 (38)	32 (26)	20 (16)	3 (2)	16 (13)	0
**By length of stay in PICU**							
PICU >28 days	43 (35)	48 (39)	14 (11)	4 (3)	0 (0)	15 (12)	0
PICU >7-28 days	27 (22)	46 (37)	26 (21)	8 (6)	0 (0)	17 (14)	0
PICU 3-7 days	12 (10)	32 (26)	40 (32)	19 (15)	5 (4)	16 (13)	0
PICU <3 days	3 (2)	14 (11)	39 (31)	33 (27)	17 (14)	18 (15)	0
**By diagnosistic category**							
Acquired brain injury	36 (29)	39 (31)	16 (13)	11 (9)	4 (3)	18 (15)	0
Severe developmental delay	11 (9)	43 (35)	39 (31)	12 (10)	3 (2)	16 (13)	0
Cancer	11 (9)	24 (19)	36 (29)	14 (11)	4 (3)	35 (28)	0
Pre-existing physical morbidity	9 (7)	38 (31)	44 (35)	12 (10)	4 (3)	17 (14)	0
Mechanically ventilated	9 (7)	38 (31)	39 (31)	18 (15)	5 (4)	15 (12)	0
Congenital heart disease	9 (7)	24 (19)	39 (31)	15 (12)	6 (5)	31 (25)	0
Respiratory illness	7 (6)	35 (28)	39 (31)	22 (18)	3 (2)	18 (15)	0
Sepsis	7 (6)	28 (23)	44 (35)	20 (16)	5 (4)	20 (16)	0
Multi-organ failure	5 (4)	14 (11)	32 (26)	35 (28)	12 (10)	26 (21)	0
Mechanically supported (e.g. extracorporeal life support)	4 (3)	8 (6)	14 (11)	13 (10)	47 (38)	38 (31)	0
**Health Care Professional team members & parent or family member(s) involvement in ERM (when applicable[#])**							
Physiotherapist	86 (70)	27 (22)	4 (3)	2 (2)	0 (0)	4 (3)	1

Confidential: For Review Only

Nurse	54 (44)	49 (40)	9 (7)	5 (4)	0 (0)	6 (5)	1
Parent or family member	36 (29)	56 (46)	19 (15)	7 (6)	1 (1)	4 (3)	1
Occupational therapist	23 (19)	34 (29)	29 (24)	20 (17)	6 (5)	7 (6)	5
Doctor	22 (18)	20 (16)	26 (21)	30 (24)	13 (11)	12 (10)	1
Play therapist	12 (10)	24 (21)	39 (34)	26 (23)	9 (8)	5 (4)	9
Dietician	10 (8)	20 (17)	12 (10)	29 (24)	37 (31)	13 (11)	3
Speech and Language therapist	4 (3)	11 (9)	40 (33)	35 (29)	20 (17)	10 (8)	4
Psychologist	3 (3)	11 (10)	24 (23)	32 (30)	30 (28)	6 (6)	18

* Percentages may not total 100 due to rounding

# Not applicable (e.g. not available in PICU) responses excluded from percent calculation.

Confidential: For Review Only

Table 3: Barriers of ERM delivery within Paediatric Intensive Care Units (n=124 respondents)*

Institutional barriers	Strongly Agree n (%)	Agree n (%)	Neutral n (%)	Disagree n (%)	Strongly Disagree n (%)	Skipped responses[#] (n)
Insufficient equipment/resources	31 (26)	52 (43)	22 (18)	13 (11)	3 (2)	3
Lack of written guidelines/protocol	31 (26)	38 (31)	35 (29)	11 (9)	6 (5)	3
Inadequate funding	30 (25)	43 (36)	28 (23)	16 (13)	4 (3)	3
Absence of champion/advocate to promote ERM	25 (21)	43 (36)	23 (19)	21 (17)	9 (7)	3
Absence of frequent ERM patient screening	25 (23)	38 (35)	24 (22)	14 (13)	8 (7)	14
Lack of physical space	17 (14)	51 (42)	17 (14)	32 (26)	4 (3)	3
Instructions that patients should not move in their bed	10 (8)	25 (21)	24 (20)	48 (40)	14 (12)	3
Consultant's permission or prescription is required prior to mobilisation	5 (4)	39 (32)	32 (26)	33 (27)	12 (10)	3
**Provider barriers**						
Slow recognition of patient readiness for ERM	19 (16)	46 (38)	19 (16)	35 (29)	2 (2)	3
Limited staffing to deliver ERM	42 (35)	54 (45)	11 (9)	13 (11)	1 (1)	3
Lack of prioritisation of ERM within patient care plans	27 (22)	45 (37)	22 (18)	24 (20)	3 (2)	3
Inadequate training	20 (17)	54 (45)	18 (15)	26 (21)	3 (2)	3
Conflicting perceptions concerning patient suitability	17 (14)	59 (49)	22 (18)	21 (17)	2 (2)	3
Lack of specific decision-making authority for ERM initiation	16 (13)	49 (40)	28 (23)	24 (20)	4 (3)	3
Safety concerns	15 (12)	55 (45)	32 (26)	18 (15)	1 (1)	3
Lack of coordination within and between clinician groups	14 (12)	44 (37)	30 (25)	27 (23)	5 (4)	4
Prolonging the current working day	4 (3)	21 (17)	43 (36)	41 (34)	12 (10)	3
**Patients barriers**						
Levels of physiological instability	48 (40)	53 (44)	12 (10)	7 (6)	1 (1)	3
Sedation level	27 (22)	64 (53)	15 (12)	13 (11)	2 (2)	3
Presence of endotracheal intubation levels	8 (7)	36 (30)	32 (26)	39 (32)	6 (5)	3
Risk of device/line/catheter dislodgement levels	8 (7)	42 (35)	32 (26)	35 (29)	4 (3)	3
Cognitive impairment/age levels	1 (1)	32 (26)	29 (24)	46 (38)	13 (11)	3
Obesity levels	6 (5)	14 (12)	35 (29)	52 (43)	14 (12)	3

Confidential: For Review Only

Patient motivation level	2 (2)	38 (31)	31 (26)	43 (36)	7 (6)	3
Family concerns levels	2 (2)	42 (35)	35 (29)	38 (31)	4 (3)	3

* Percentages may not total 100 due to rounding # Skipped responses excluded from percent calculation.

Confidential: For Review Only
